# Evidence-based sustainable business model design for agrifood side-stream biostimulants

**DOI:** 10.1371/journal.pone.0343143

**Published:** 2026-07-08

**Authors:** Federico Zilia, Luca Ferraro, Jacopo Bacenetti, Luigi Orsi

**Affiliations:** Department of Environmental Science and Policy, University of Milan, Milan, Italy; University of Bologna, ITALY

## Abstract

Circular bioeconomy initiatives often valorise food-processing residues into bio-based inputs, yet environmental gains depend on how technologies are implemented and governed across multiple actors. This study evaluates a pathway that converts leek processing residues into a bioactive extract used as a plant biostimulant in greenhouse lettuce production and develops an evidence-based procedure to design a sustainable business model around it. We conducted a cradle-to-gate Life-Cycle Assessment (LCA) of leek extract production and a comparative LCA of lettuce cultivation for a baseline system and an alternative system applying the extract, using the Environmental Footprint (EF) 3.1 midpoint indicators. The alternative scenario improved most indicators, with consistent reductions in acidification, eutrophication and freshwater ecotoxicity, while selected trade-offs were associated with the upstream extract supply chain, particularly in resource-related and process-sensitive impact categories. We then embedded these multi-impact hotspot signals into an ecosystem-oriented Sustainable Business Model Canvas (SBMC) and an experimentation logic, specifying who must act on each hotspot (processors, extract producers, labs, farmers) and how mitigation and verification can be financed through value capture (product-service bundles, monitoring, and conditional sustainability claims). The approach links LCA evidence to actionable business model choices, supporting credible scaling of residue-based agrifood innovations.

## 1. Introduction

Food loss and waste (FLW) is widely recognised as a major sustainability challenge across agri-food systems, with direct implications for climate change, land and water use, eutrophication and biodiversity loss [1–3] At the same time, processing and distribution chains are reconfigured by consumer demand for convenience and safety, expanding fresh-cut and minimally processed ‘ready-to-cook’ products; these chains concentrate residues spatially and temporally, creating both a waste management burden and a circular bioeconomy opportunity [4,5].

In the European Union, circular economy (CE) policy and bioeconomy strategies increasingly encourage higher-value utilisation of side-streams by applying cascading principles and avoiding low-value downcycling [6,7]. However, scholars warn that circular strategies can generate rebound effects or burden shifting if the energy and material intensity of valorisation outweighs avoided burdens, or if environmental improvements are claimed on narrow indicators such as climate while ignoring toxicity, water scarcity or critical materials [8,9]. These concerns underline the need for multi-impact assessment and transparent accounting when designing circular innovations [10,11].

Plant biostimulants represent a fast-growing category of bio-based agricultural inputs. EU Regulation (EU) 2019/1009 provides a harmonised market framework and a functional definition of biostimulants as products that stimulate plant nutrition processes to improve nutrient use efficiency, stress tolerance, quality traits or nutrient availability, independently of nutrient content [12]. Scientific reviews highlight that biostimulants span diverse categories, including microbial inoculants, humic substances, protein hydrolysates and amino acids, and botanical extracts [13–16]. While biostimulants are often positioned as sustainable alternatives, their net environmental performance is contingent on production pathways and on the extent to which they substitute conventional inputs or stabilise yields under stress [16,17].

Against this background, the SOMMELIER project, led by the University of Milan, develops a circular pathway to valorise leek (*Allium porrum L.*) residues generated in “prima gamma evoluta” processing (an Italian term referring to fresh-cut, minimally processed ready-to-cook vegetables). In the focal supply chain, leek cultivation covers roughly 25 ha out of 65 ha managed by the producer organisation; processing generates a substantial non-edible fraction (roots, leaves and green bulb parts). While 10–15% of edible fractions are reused, an estimated 30–40% remains under-valorised, corresponding to approximately 225–300 metric tons per year. The project employs pilot-scale Naviglio extraction equipment to obtain bioactive extracts for agricultural use, initially demonstrated in lettuce production.

Life cycle assessment (LCA) is well established for quantifying environmental impacts of products and technologies [18–20]. In circular bioeconomy contexts, LCA is increasingly used to identify hotspots and to compare design options under transparent assumptions [5,21]. Early applications of LCA to horticultural crops have already highlighted the relevance of leek as a case study for assessing environmental performance in agriculture, comparing organic and conventional production systems and identifying fertilisation and energy use as major environmental hotspots [22]. Despite this tool can provides a relevant support for decision making, notably in waste management [23], the LCA alone does not specify how value is created, delivered and captured across multiple actors. Moreover, it does not represent how costs and benefits should be shared, or how environmental hotspots can be financed and governed during scaling [24,25]. Conversely, sustainable business model frameworks provide conceptual tools for designing multi-dimensional value propositions but are often criticised for lacking quantified environmental evidence to guide design priorities [26–28].

Despite growing research on circular bioeconomy and sustainable business models, the integration of multi-indicator environmental assessment with business model design remains limited. In particular, existing studies often apply LCA to evaluate environmental impacts, while sustainable business model frameworks focus on value creation and governance mechanisms, but the methodological link between these two analytical perspectives remains underdeveloped.

Accordingly, this paper responds to this gap by integrating LCA evidence with sustainable business model innovation for circular side-stream valorisation. This study contributes to the literature in three main ways. First, it provides an empirical demonstration of how multi-indicator LCA results can be translated into actionable business model design choices in circular bioeconomy contexts. Second, it proposes a transparent “hotspot-to-canvas” translation protocol that systematically links environmental hotspot drivers to specific Sustainable Business Model Canvas (SBMC) elements, actor responsibilities, and governance mechanisms. Third, it illustrates how environmental assessment can support ecosystem-level business model experimentation, helping actors design financeable mitigation strategies for environmental trade-offs rather than relying on descriptive sustainability claims. Building on Ecologies of Business Models Experimentation (EBME), we combine this perspective with the Sustainable Business Model Canvas (SBMC) to translate quantified environmental hotspots – identified using the EF 3.1 midpoint method, a harmonised set of multi-indicator impact categories, and refined through contribution analysis – into explicit business model design choices and value capture mechanisms, thereby improving transparency and reducing interpretive inference [29–31]. Moreover, the proposed method is designed to be transferable, as any residue-to-input pathway can be analysed by applying the same steps once a process-based Life Cycle Inventory (LCI) and a clearly defined downstream use scenario are available.

The paper addresses three research questions: *(RQ1)* What are the main environmental hotspots and trade-offs of producing leek extract and applying it in lettuce cultivation compared with a baseline scenario? *(RQ2)* How can LCA evidence be operationalised into sustainable business model design using SBMC in a multi-actor ecosystem? *(RQ3)* What managerial, governance and policy implications follow for scaling circular side-stream valorisation credibly without burden shifting?

The manuscript is organized as follows: Section 2 reviews the literature on food waste valorisation, biostimulants, life cycle assessment and sustainable business models for circular bioeconomy pathways. Section 3 describes the case study, the LCA modelling approach and the sustainable business model design procedure. Section 4 reports the LCA and sustainable business model results. Section 5 discusses implications, limitations and future research directions, and Section 6 concludes.

## 2. Background and related literature

### 2.1. Circular bioeconomy strategies for agri-food side-streams

FLW valorisation is located at the interface of CE, industrial ecology and the bioeconomy [7,32]. While CE is widely promoted in policy and practice [6,33], academic work emphasises that CE is a contested and multi-interpretation concept with diverse definitions and priorities [9,10]. These conceptual ambiguities matter because they shape what organisations measure and legitimise when implementing circular initiatives [11,34].

In agri-food, cascading and biorefinery perspectives argue that side-streams should be directed to applications with higher functionality and longer value retention [4,5]. Reviews of food manufacturing waste valorisation document options ranging from functional ingredients and biomaterials to composting and anaerobic digestion, with feasibility constrained by contamination, seasonality and logistics [4,5]. Industrial symbiosis scholarship highlights that the value of side-streams depends on local actor networks and infrastructural proximity, suggesting that circular pathways are ‘place-based’ and governance-intensive [35,36].

However, CE scholars stress the risk of the ‘circular economy rebound’: closing loops can increase total throughput or shift impacts elsewhere, for example through increased processing or transport intensity [8]. Accordingly, circular strategies should be evaluated through multi-impact assessment rather than single-indicator claims [9]. Moreover, governance and contract design are critical because residue quality, segregation and timing strongly influence downstream process efficiency and environmental performance [37,38].

Plant biostimulants are increasingly framed as enabling more resource-efficient and resilient agriculture, potentially contributing to nutrient management, stress tolerance and yield stability [13–15]. Empirical studies report diverse effects across crops and contexts, including improved root development, enhanced nutrient uptake and stress mitigation [16,17].

Yet biostimulants also raise sustainability questions. First, producing bio-based inputs depend on extraction pathways, solvents, energy intensity and upstream feedstock logistics [5,21]. Second, agronomic efficacy must be robust across conditions to deliver net system-level benefits [16]. Third, regulatory and market legitimacy depend on safety, traceability and claim verification, particularly where complex biological matrices can contain contaminants [12].

Consequently, a sustainability-oriented innovation approach should couple agronomic and technological testing with robust environmental assessment and with business model design that allocates responsibilities, costs and benefits across the actor network [28,29].

### 2.2. LCA approaches

LCA provides a systematic framework to quantify environmental impacts across life cycle stages, typically in line with ISO 14040/14044 [18–20]. In side-stream valorisation, methodological choices are salient: whether residues are treated as wastes carrying no upstream burdens, or as by-products requiring allocation; how co-products are handled; and whether avoided products should be modelled via system expansion or substitution [39,40]. Because these choices can change results and their interpretation, early-stage studies often adopt attributional approaches with explicit assumptions and sensitivity logic [20,41].

The Environmental Footprint (EF) methods offer a harmonised midpoint indicator set widely used in European contexts [42]. EF indicators enable multi-criteria hotspot analysis, including climate change, acidification, eutrophication, particulate matter, photochemical ozone formation, ozone depletion, human toxicity, freshwater ecotoxicity, resource use (fossil; minerals/metals) and water deprivation [42]. For bio-based extraction processes, toxicity and mineral/metal indicators can be decision-relevant because solvents, catalysts and equipment materials may dominate these categories even when climate performance appears favourable [42,43].

The role of LCA in innovation is increasingly conceptualised as decision support: hotspot identification can guide iterative redesign and reduce the risk of ‘environmental lock-in’ as technologies scale [24]. However, translating hotspots into operational changes requires organisational and inter-organisational mechanisms: who invests, who benefits and how improvements are verified [25].

### 2.3. Sustainable business models for circularity

SBM research extends conventional business model thinking by integrating environmental and social value creation alongside economic value capture [44,45]. A business model is commonly defined as the system of activities through which an organisation creates and captures value [46,47], while sustainability-oriented work emphasises that value should be created for a broader set of stakeholders and within ecological limits [28,48,49].

Bocken et al. [29] synthesise SBM archetypes, including ‘create value from waste’, ‘maximise material and energy efficiency’ and ‘substitute with renewables and natural processes’. Circular economy business model pattern research further classifies recurring configurations and highlights barriers for SMEs, including finance, market demand and collaboration challenges [35,40].

Canvases such as the Business Model Canvas proposed by Osterwalder and Pigneur [50] provide an accessible structure for describing business model elements, and sustainability extensions such as the Triple Layered Business Model Canvas (TLBMC) add environmental and social layers [51]. The TLBMC has been valuable for visualising sustainability considerations and supporting ideation, particularly for single organisations or product-service offerings [51–53].

However, circular bioeconomy innovations frequently operate across multiple firms and institutions. Innovation ecosystem research and circular business model studies highlight that successful circularity requires complementary business models, coordination among actors and shared infrastructure [38,54]. The ‘ecologies of business model experimentation’ (EBME) perspective frames business model innovation as experimentation within a system of interacting business models, where synergies and trade-offs must be understood and governed [30,55]. This perspective is particularly suitable for producer-organisation-led projects such as SOMMELIER.

To integrate LCA evidence into business model design, a framework must move beyond listing impacts and instead specify actionable levers, responsibilities and financing mechanisms. While the TLBMC provides a useful layered representation of environmental and social dimensions, applications can remain descriptive and centred on a single focal organisation, with less emphasis on ecosystem coordination and on how environmental improvements are funded through value capture [27,51].

The SBMC proposed by Bocken [56] builds on the logic of value creation, delivery and capture while embedding a Profit-People-Planet value proposition at the core. It is compatible with stakeholder-oriented sustainability value creation [49] and supports an ecosystem interpretation: the canvas can represent a focal proposition that depends on complementary partner models and governance arrangements [30,38].

Critically, SBMC blocks (key activities, resources, cost structure) provide a direct bridge to LCA hotspots because hotspots are typically associated with specific activities and resource uses [24].

A growing literature seeks to operationalise environmental assessment at the business model level. BM-LCA approaches conceptualise the business model as the object of analysis and compare environmental impacts per unit of economic value, enabling decisions about business configurations [25]. Other strands integrate LCA into early-stage experimentation, emphasising that environmental impacts should be measured and forecasted during pilots to avoid lock-in to high-impact designs [54,55].

Despite these advances, there remains a need for case-based demonstrations that use standard product-focused LCA outputs as inputs to ecosystem-oriented SBM design, producing explicit, financeable redesign levers and governance proposals [26,27]. This work addresses this gap by linking EF midpoint hotspot analysis to SBMC blocks and by articulating how value capture can fund mitigation of trade-offs.

## 3. Materials and methods

### 3.1. Study design and system modelling

The environmental performance of a leek-derived biostimulant extract and its application in greenhouse lettuce cultivation was assessed through Life Cycle Assessment (LCA) in accordance with ISO 14040 and ISO 14044 standards [57]. The study was designed as a comparative assessment of two lettuce production systems: (i) a conventional cultivation scenario (baseline) and (ii) an alternative scenario where the leek-based extract was applied during crop management.

System boundaries were defined according to a cradle-to-gate perspective (resource extraction to factory gate [58]). For lettuce cultivation, the model included upstream production of agricultural inputs (fertilisers, plant protection products, seeds, electricity and fuels), crop establishment, fertilisation, irrigation, crop management, harvesting and on-farm transport, as well as direct emissions to air, soil and water associated with agricultural practices. For the leek extract, the system included collection of leek residues, washing, extraction and thermal treatment steps (including hydrodistillation), and the generation of solid and liquid co-products. Infrastructure, packaging and end-of-life stages were excluded from the analysis.

The environmental burdens associated with extract production were integrated into the alternative lettuce scenario using the application rate adopted in the experimental trials. Impacts related to the production of 1 L of extract were linked to lettuce cultivation by accounting for cultivated area and corresponding crop yield, allowing results to be expressed consistently per kilogram of fresh lettuce.

### 3.2. Functional units, inventory data and impact assessment

Two functional units (FUs) were defined to reflect the dual objective of the study. The first FU (FU1) was defined as 1 L of leek extract to quantify impacts associated with the production of the biostimulant. The second FU (FU2) was defined as 1 kg of fresh lettuce at the farm gate and represents the reference unit for comparing baseline and alternative cultivation systems.

The Life Cycle Inventory was developed using a combination of primary and secondary data. Primary data were collected through direct interviews with farmers using specifically designed questionnaires and from experimental activities, capturing farming operations and the production factors used. The extract application rate was 1 L m ⁻ ², and lettuce yields were 1.16 kg m ⁻ ² for the baseline scenario and 1.33 kg m ⁻ ² for the alternative scenario with leek extract. The application rate of 1 L m ⁻ ² was defined to ensure a uniform distribution of the extract across the cultivated area, following standard agronomic practices for soil-applied liquid biostimulants in greenhouse conditions. This volume was calibrated to achieve sufficient soil moisture for bioactive compound uptake without causing leaching or runoff.

Secondary data were used to model upstream production of agricultural inputs, energy supply, and emissions related to fertiliser use and nutrient cycles, based on established modelling approaches and available databases (e.g., Ecoinvent).

The analysis of impact assessment was performed using the characterisation factors provided by the Environmental Footprint (EF) 3.1 method, the impact assessment method endorsed by the European Commission [59]. The impact following impact categories were considered: climate change, acidification, particulate matter formation, freshwater ecotoxicity, freshwater, terrestrial and marine eutrophication, human toxicity (cancer and non-cancer), ozone depletion, photochemical ozone formation, fossil resource use, and mineral and metal resource use. LCA modelling was carried out using SimaPro software.

### 3.3. Sustainable business model design

**Fig 1:**
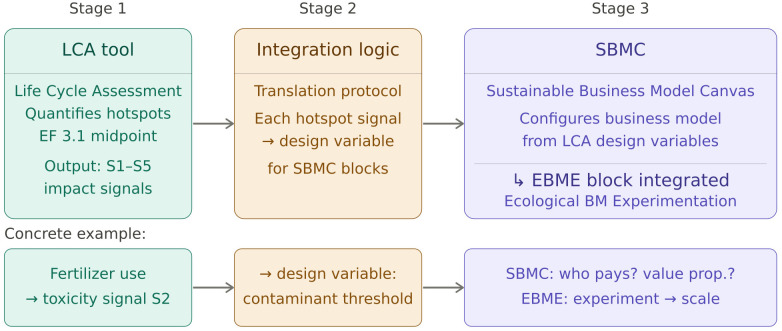
Integrated LCA-SBM framework.

We combine the EBME perspective with the SBMC to structure business model innovation around sustainability aims, actor interdependencies, trade-offs and experimentation priorities [27,28]. As illustrated in Fig 1, the methodological approach follows an integrated modelling logic that links environmental assessment and business model design through a structured translation process. Environmental hotspot signals generated by the LCA analysis (EF 3.1 midpoint indicators and contribution analysis) are used as decision-relevant inputs and progressively translated into operational variables, intervention levers and governance mechanisms within the SBMC. In this way, environmental impacts are directly connected to business model elements such as key activities, resources, cost structure and value capture, enabling the identification of actionable and financeable mitigation strategies across the actor ecosystem. This integration provides a transparent and replicable way to move from multi-indicator environmental evidence to concrete business model design choices, while allowing specific levers and responsibilities to adapt to different residue valorisation contexts. The three stages represented in Fig 1 reflect this logic, moving from environmental quantification (Stage 1), to hotspot-to-variable translation (Stage 2), and finally to strategic business model configuration (Stage 3).

## 4. Results and discussion

### 4.1. LCA results

#### 4.1.1. Environmental impacts of leek extract production (FU: 1 L of extract).

The production of 1 L of leek extract was associated with measurable environmental impacts across all the analysed categories, reflecting the experimental and pilot-scale nature of the process. Impact values were particularly influenced by energy consumption and processing steps required for extraction and thermal treatment.

Table 1 reports the absolute environmental impacts associated with the production of leek extract.

**Table 1:** Absolute impacts for the functional unit (1 L of leek-extract).

Impact Categories	Unit of measure	Impact
Acidification	mol H+ eq x10^-4^	0.86
Climate change	kg CO2 eq x 10^−1^	0.11
Ecotoxicity, freshwater	CTUe	0.11
Particulate matter	disease inc. x10^-9^	0.79
Eutrophication, marine	kg N eq x10^-4^	0.13
Eutrophication, freshwater	kg P eq x10^-5^	0.70
Eutrophication, terrestrial	mol N eq x10^-3^	0.13
Human toxicity, cancer	CTUh x10^-10^	0.15
Human toxicity, non-cancer	CTUh x10^-9^	0.67
Ozone depletion	kg CFC11 eq x10^-9^	0.82
Photochemical ozone formation	kg NMVOC eq x10^-4^	0.49
Resource use, fossils	MJ	0.14
Resource use, minerals and metals	kg Sb eq x10^-6^	0.77
Water use	m3 depriv.	0.500

Overall, the extraction phase contributes across all impact categories, indicating a relatively impact-intensive process at this pilot stage. The impact profile is characterised by relatively higher burdens in acidification and ozone depletion. By contrast, climate change and fossil resource use remain comparatively limited, suggesting that the environmental profile is not primarily driven by carbon-intensive energy use but rather by process-specific inputs and emissions. Eutrophication and particulate matter formation show intermediate contributions, indicating a more distributed impact pattern across categories. Resource-related impacts are mainly associated with mineral and metal use, whereas water use is entirely linked to direct consumption and expressed in terms of resource deprivation.

#### 4.1.2. Environmental impacts of lettuce cultivation (FU: 1 kg of lettuce).

When impacts are expressed per kilogram of lettuce, the alternative cultivation system shows a general reduction across most impact categories compared with the baseline (Table 2).

**Table 2:** Absolute values related to the functional unit of 1 kg of fresh lettuce.

Impact Categories	Unit of measure	Baseline	Alternative	Δ %
Acidification	mol H+ eq x10^-2^	1.089	0.954	−12.4%
Climate Change	kg CO2 eq x10^-1^	0.609	0.603	−1.0%
Ecotoxicity, freshwater	CTUe	0.817	0.701	−14.2%
Particulate Matter	disease inc. x10^-7^	0.749	0.658	−12.1%
Eutrophication, marine	kg N eq x10^-2^	0.372	0.325	−12.6%
Eutrophication, freshwater	kg P eq x10^-4^	0.456	0.428	−6.1%
Eutrophication, terrestrial	mol N eq x10^-1^	0.482	0.421	−12.7%
Human Toxicity, cancer	CTUh x10^-10^	0.202	0.259	+28.2%
Human Toxicity, non-cancer	CTUh x10^-9^	0.496	0.729	+47.0%
Ozone Depletion	kg CFC11 eq x10^-8^	0.179	0.146	−18.4%
Photochemical Ozone Formation	kg NMVOC eq x10^-3^	0.291	0.284	−2.4%
Resource Use, fossil	MJ	0.753	0.744	−1.2%
Resource Use, mineral and metal	kg Sb eq x10^-6^	0.309	0.663	+114.6%
Water Use	m3 depriv.	2.988	2.951	−1.2%

Improvements are particularly evident for acidification and eutrophication indicators, which are among the most relevant environmental pressures in agricultural systems. Climate change and fossil resource use also show slight reductions, although differences remain limited in absolute terms.

At the same time, the results reveal the presence of trade-offs across impact categories. In particular, resource use related to minerals and metals increases in the alternative scenario, reflecting the additional material and process requirements associated with the extract supply chain. Other impact categories show more heterogeneous patterns, indicating that the environmental benefits of the alternative system are not uniformly distributed.

The comparison suggests that the use of the leek-derived extract can deliver environmental improvements in key agricultural impact categories, while introducing additional burdens in others. This confirms the importance of adopting a multi-indicator perspective when evaluating circular bioeconomy solutions, as no single scenario clearly dominates across all impact dimensions.

Although direct comparison with previous studies is not possible due to the novelty of the system, the results provide a robust basis for identifying environmental hotspots and guiding further optimisation of both the extraction process and its integration into the cropping system.

#### 4.1.3. Relative contribution analysis (baseline vs alternative).

**Fig 2:**
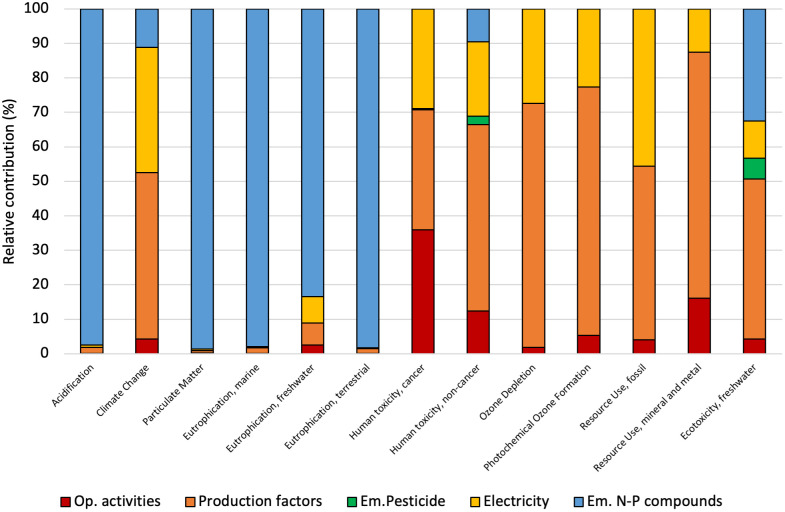
Baseline scenario related to the functional unit of 1 kg of fresh lettuce.

**Fig 3:**
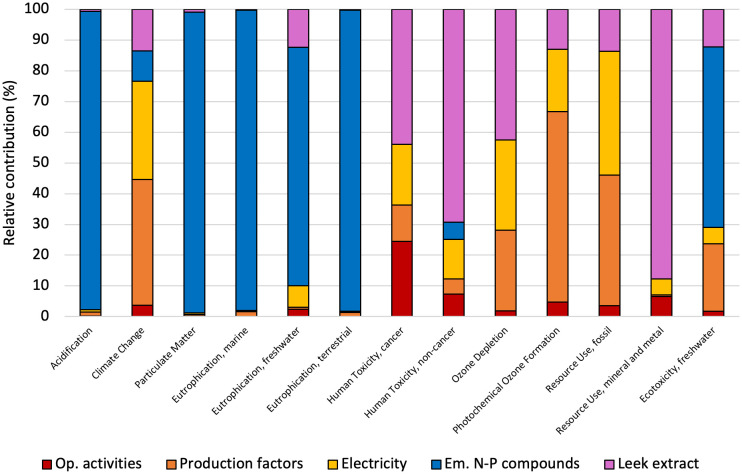
Alternative scenario related to the functional unit of 1 kg of fresh lettuce.

Fig 2 and Fig 3 provide a clearer understanding of the processes driving the impact profiles of the baseline and alternative lettuce systems. In the baseline scenario (Fig 2), the contribution structure is strongly shaped by conventional agricultural emissions. Emissions from nitrogen and phosphorus compounds account for almost the entire burden in acidification, particulate matter formation and eutrophication-related categories, reaching approximately 97–98% in several indicators. This confirms that, in the reference system, the main environmental pressure is associated with nutrient-related field emissions rather than with the upstream production of the biostimulant input.

Other impact categories show a less concentrated pattern. Climate change, for example, is mainly driven by production factors and electricity use, which together account for most of the impact. A similar pattern appears for fossil resource use and ozone-related indicators, where electricity and production factors remain central drivers. This distinction is relevant because it shows that not all impacts can be reduced through the same intervention: nutrient-related categories require agronomic and field-emission strategies, while energy- and input-related categories require changes in supply, process efficiency and procurement choices.

The alternative scenario (Fig 3) partly preserves this structure, since nitrogen and phosphorus emissions still dominate acidification and eutrophication-related indicators. The introduction of leek extract, however, adds a new upstream driver to the system. Its contribution is limited in some categories, such as climate change, where it accounts for about 14%, but becomes much more relevant in selected resource- and toxicity-related indicators. The clearest example is mineral and metal resource use, where the leek extract accounts for nearly 88% of the total contribution. This result explains why the alternative scenario can reduce several agriculture-related impacts while still generating trade-offs in categories linked to the extract supply chain.

The comparison between Fig 2 and Fig 3 therefore suggests that the environmental performance of the alternative system depends on two conditions. First, the agronomic benefit of the extract must be sufficient to maintain reductions in categories linked to conventional crop production. Second, the upstream burdens of extract production must be progressively reduced through process optimisation, electricity sourcing, auxiliary input selection and material-efficient equipment choices. This interpretation is consistent with the need for multi-indicator assessment in circular bioeconomy pathways, where improvements in one set of categories may coexist with burden shifting in others [9,42].

These contribution results provide the analytical basis for the business model design developed in the following section. In particular, they identify which actors and activities should be targeted by mitigation strategies: farmers and agronomic advisors for nutrient-related field emissions, and extract producers, technology providers and laboratories for energy use, material requirements, quality control and verification. The contribution analysis therefore acts as the bridge between the LCA results and the LCA-informed SBMC, translating environmental hotspots into specific operational and governance priorities [24].

### 4.2. Sustainable business model results

**Fig 4:**
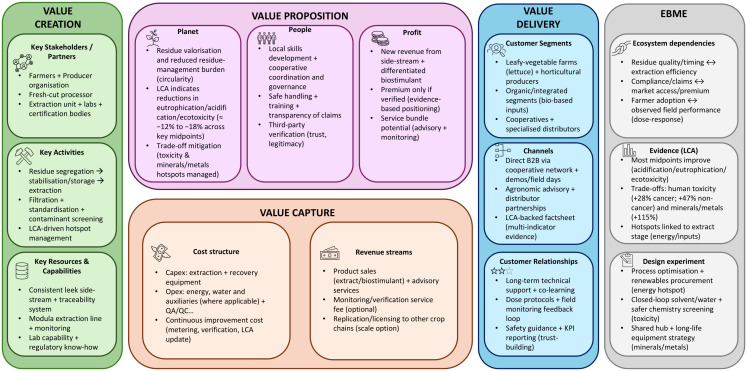
SBMC-EBME canvas for SOMMELIER.

The integrated SBMC and EBME representation is shown in Fig 4, while Table 3 reports the resulting business model configuration and the LCA-informed design levers. The purpose of this section is not to present a final commercial model, but to show how the environmental evidence generated by the LCA can inform the design of a business model for the SOMMELIER pathway. In this case, the LCA results point to a double condition: the leek extract can contribute to lower impacts in several agronomic categories per kilogram of lettuce, but its production also introduces upstream burdens that need to be managed before scaling.

**Table 3:** Sustainable Business Model Canvas applied to SOMMELIER: case configuration and LCA-informed environmental design levers.

SBMC building block	SOMMELIER configuration (what the BM is)	LCA-informed environmental levers (readable, measurable)
Key stakeholders/ partners	Producer organisation + farmers; fresh-cut processor; extraction operator; technology provider; analytical labs; agronomic trial partners; conformity bodies; horticultural farms; logistics; public innovation programme.	Partner selection with minimum sustainability conditions: *(i)* extraction site powered mainly by renewables (high renewable share); *(ii)* residue supply agreements specifying quality and timing to avoid extra processing; *(iii)* annual third-party verification of traceability and environmental claims.
Key activities	Residue segregation; storage/stabilisation; extraction (water/ethanol); filtration/standardisation; QA/QC; agronomic formulation & guidance; compliance; marketing & support.	Focus on the LCA hotspots of extraction: *(i)* reduce energy use per litre of extract vs the pilot baseline; *(ii)* adopt water/solvent recirculation where feasible; *(iii)* introduce a safer-chemistry check for auxiliaries to address the toxicity trade-off; *(iv)* update LCA after major process changes.
Key resources & capabilities	Stable residue stream; extraction equipment; lab for bioactives/contaminants; agronomic expertise; regulatory capability; traceability/data; logistics.	Capability built around evidence: *(i)* batch-level metering for energy and water; *(ii)* traceability to support multi-indicator environmental communication; *(iii)* equipment strategy prioritising long lifetime and shared use (hub model) to reduce resource-related burdens over time.
Customer segments	Leafy vegetable farms; organic/integrated; distributors for bio-based inputs; cooperatives seeking circular inputs.	Target customers valuing verified impacts: offer a “verified sustainability” tier only when LCA shows net improvements across multiple indicators and no significant increase in toxicity/resource use; maintain a conservative claim strategy to prevent burden shifting.
Channels	Direct B2B via cooperative; demo farms; agronomic advisory; distributor partnerships; innovation showcases.	Evidence-based go-to-market: provide customers with a short environmental factsheet reporting a small set of indicators (e.g., climate change, eutrophication/acidification, toxicity/resource-use flags) and the date of the latest LCA update.
Customer relationships	Technical support & co-learning; performance monitoring; long-term supply; feedback loops; training for safe handling.	Monitoring to protect environmental performance: *(i)* guidance to keep application within recommended dose (“minimum effective dose” principle); *(ii)* seasonal feedback from farms to update performance assumptions; *(iii)* safe handling training to address concerns linked to toxicity categories.
Value proposition – Planet	Valorise leek residues; reduce disposal; improve lettuce performance across multiple EF categories; reduce reliance on conventional inputs where feasible.	Two-part environmental promise: *(i)* preserve improvements in key categories (e.g., eutrophication/acidification/ecotoxicity) shown by the LCA; *(ii)* explicitly manage trade-offs by committing to reduce the toxicity and mineral/metal resource signals through process redesign (energy source, solvent choice, recovery loops), with annual verification.
Value proposition – People	Jobs/skills; cooperative governance; farmer income diversification; compliance/transparency under EU fertilising products regulation.	Measurable co-benefits: training delivered annually; transparent reporting of compliance steps; stakeholder inclusion and fair value sharing, supported by traceability and third-party checks.
Value proposition – Profit	New revenue stream; lower waste handling cost; differentiated biostimulant; sustainability premium; leverage public funding for early investment.	Profit aligned with mitigation: pricing includes a small “verification and mitigation” component covering monitoring, recovery and testing; premiums are conditional on maintaining verified multi-indicator improvements (avoid greenwashing risk).
Cost structure	Capex extraction/recovery; Opex energy/water/solvents/labour; testing/compliance; logistics; advisory.	Cost priorities guided by hotspots: invest first in options that reduce the main burdens (energy, water/solvent use, toxicity-related auxiliaries); treat monitoring and recovery as enabling costs required for verified claims. To make feasibility transparent, key cost items are tracked in allocable units (e.g., € per batch for QA/QC and third-party verification; kWh per litre and €/kWh for energy; CAPEX amortisation per litre in hub configurations).
Revenue streams/ value capture	Product sales; advisory & monitoring services; licensing/replication; shared savings with processor & farms.	Value capture linked to evidence: additional revenues (premium/service fees) depend on verified environmental performance; shared-savings contracts align residue quality and process efficiency incentives across the chain. Operationally, the verification-and-mitigation component can be implemented as a per-litre surcharge equal to (QA/QC + monitoring + verification cost per batch)/(litres per batch) plus an energy term based on measured kWh L ⁻ ¹, and applied only to the ‘verified’ tier.

At the current pilot stage, precise price estimates would not be robust. For this reason, the economic linkage is framed as an accounting logic that can be updated once operational data become available. The main enabling costs are expected to arise from quality assurance and quality control testing, contaminant screening, traceability, third-party verification and electricity use during extraction. These costs can be assigned first to each production batch and then to each litre of extract, for example by allocating laboratory and verification costs over batch volume and adding the measured energy cost per litre. This provides a practical way to connect hotspot mitigation to the future price structure without overstating the level of economic precision currently available.

The resulting business model depends on coordination across the local value chain. Farmers and the producer organisation secure the residue stream and test the agronomic use of the extract; the fresh-cut processor is central to residue segregation and preservation; the extraction operator and technology provider control energy use, process yield and recovery options; laboratories support safety testing, bioactive profiling and compliance under the EU fertilising products regulation; distributors and advisory networks influence adoption by horticultural farms. This interdependence is consistent with circular innovation ecosystem research, where environmental performance depends on coordinated routines, standards and shared infrastructure rather than on a single firm-level decision [35,38,54].

The LCA evidence shapes the canvas in a specific way. The positive signal from the downstream crop assessment supports the value proposition of transforming a local side-stream into a bio-based agricultural input, in line with sustainable business model archetypes such as creating value from waste and substituting with renewable or natural processes [29,60]. At the same time, the contribution analysis prevents the business model from relying on a generic circularity claim. The categories in which the extract supply chain becomes relevant require explicit design commitments, particularly extraction efficiency, low-carbon electricity, careful selection and recovery of auxiliary inputs, quality testing and material-aware equipment choices. This is important because circular solutions can still shift burdens across impact categories if they are evaluated only through narrow environmental claims [9,42].

In practical terms, the key managerial issue is to reduce the extraction burden per unit of agronomic function delivered. This means improving energy use per litre, identifying the minimum effective dose in the field, and verifying whether the crop-level benefits are sufficient to justify the impacts of producing the extract. The SBMC translates these points into business model components: key activities include extraction optimisation and application guidance; key resources include testing capacity and traceability data; the cost structure includes energy, laboratory analysis and compliance; and revenue streams may combine product sales with advisory, monitoring or verification services. In this way, the LCA results become inputs for managerial and governance decisions rather than remaining only environmental indicators [24,27,28].

Table 3 should therefore be read as an ecosystem-level business model hypothesis for SOMMELIER. It identifies the decisions that must be tested before scaling: who controls each hotspot, which operational variables should be monitored, and how the costs of mitigation and verification can be covered. The central implication is that environmental credibility has to be built into the operating model through measured process data, safety checks and conditional sustainability claims, while value capture mechanisms must be able to support these activities. This links the EBME logic of iterative experimentation to the SBMC representation and clarifies how hotspot mitigation, actor responsibility and economic incentives are connected [29,49,55].

While Table 3 details the operational translation of LCA hotspots into SBMC blocks, Fig 4 provides a visual synthesis of the resulting ecosystem-level business model and of the connections between actors, mitigation levers and value capture mechanisms.

### 4.3. Implementation and scaling logic

Implementing the proposed sustainable business model requires explicit decisions on (i) the location and scale of extraction capacity, (ii) residue stabilisation and quality management, and (iii) the governance arrangements that coordinate processors, producers and downstream customers. These choices affect not only economic feasibility but also the system-wide environmental profile, because the LCA results indicate that benefits at crop level coexist with non-negligible trade-offs driven by the extract supply chain and its auxiliary inputs. To test the sensitivity of the proposed business model and its environmental hotspots to different logistical and scale configurations, two stylised implementation scenarios are therefore discussed as alternative pathways for operationalising the SBMC hypothesis (Table 3) and for interpreting the ecosystem-level design choices represented in Fig 4.

*Scenario S1 (decentralised, near-source extraction):* This scenario places small modular units close to the fresh-cut processor, thereby reducing residue transport distances and allowing rapid processing of a time-sensitive biomass stream. This configuration can lower logistics burdens and mitigate quality deterioration, potentially reducing the need for stabilisation steps. However, decentralised systems may show higher unit costs and may struggle to achieve high recovery efficiency and robust monitoring at small scale, particularly when advanced solvent/water recirculation or energy-efficiency upgrades require capital-intensive equipment. From a hotspot-mitigation perspective, S1 performs best when paired with operational controls that prevent burden shifting, including stringent residue segregation protocols, minimum effective dose strategies, and performance monitoring that links application guidance to field conditions.

*Scenario S2 (regional extraction hub):* This scenario aggregates residues from multiple processors into a shared facility, enabling economies of scale in extraction, quality control and utilities management. A hub can justify investments in best-available recovery systems (e.g., closed-loop water/solvent management) and in contracted low-carbon electricity supply, and it can institutionalise third-party verification and traceability as part of routine operations [38,54]. These features align directly with the LCA-informed design levers in Table 3 and can be particularly relevant for managing toxicity- and mineral/metal-related trade-offs through standardised procurement, safer-chemistry screening and longer-lived equipment strategies. The main drawbacks are coordination complexity and the need for governance mechanisms that define residue quality standards, scheduling rules, and transparent benefit sharing among participating actors [30].

Across both scenarios, the SBMC emphasises that environmental performance is a joint outcome of process design and ecosystem governance. Accordingly, Table 3 and Fig 4 should be interpreted as a modular blueprint: the business model building blocks remain stable, while the configuration of stakeholders, cost structure and value capture mechanisms can be adapted to the chosen scaling pathway. In practical terms, S1 prioritises proximity and flexibility, whereas S2 prioritises infrastructure efficiency and measurable hotspot mitigation. These scenario logics provide a decision frame for implementation and for future empirical testing of the business model hypothesis under real-world operating conditions

## 5. Discussion and managerial implications

SOMMELIER illustrates an empirically common pattern in circular bioeconomy innovations: the potential for improvements in multiple impact categories downstream coupled with trade-offs driven by upstream upgrading [5,8]. The alternative lettuce scenario indicates reductions in eutrophication and acidification-related indicators-highly salient in agriculture-yet significant increases in toxicity and mineral/metal resource use. These results caution against ‘circular-equals-sustainable’ narratives and reinforce calls for multi-indicator evaluation to avoid burden shifting [9,42]. Transferability beyond the focal case depends primarily on the availability of two key conditions. First, it requires access to a process-based inventory describing the residue upgrading step, since the environmental assessment relies on detailed information about material and energy flows associated with the transformation of waste into a usable input. Second, it requires a clear definition of the downstream function through which environmental and economic benefits are generated, such as reductions in fertiliser or pesticide use, or improvements in yield stability. When these elements are specified, the analytical approach developed in this study can be applied in other contexts following a structured but adaptable logic. In practice, the process involves conducting an LCA using a harmonised methodology (here the EF 3.1 framework) to identify the environmental hotspots associated with the production pathway. These quantified hotspot drivers can then be systematically translated into specific business model design choices within the Sustainable Business Model Canvas, linking environmental performance to operational decisions, actor responsibilities, and governance arrangements.

Importantly, the approach also emphasises the need to progressively integrate operational data as the pathway moves from experimental to scaled implementation. For this reason, the methodological section clarifies which variables should be systematically monitored in future applications, including energy requirements per unit of output, material recovery rates, the minimum effective agronomic dose, and quality assurance thresholds. Tracking these parameters over time allows both the LCA results and the associated business model assumptions to be updated as empirical evidence accumulates. In this way, the proposed LCA-business model integration is not limited to the specific case analysed here, but offers a replicable framework that can support the design and evaluation of similar residue-to-input valorisation pathways in other agri-food contexts.

For biostimulants specifically, toxicity hotspots are governance-relevant because legitimacy and compliance depend on safety and traceability [12,13]. As such, the business model must not only aim to valorise residues but also embed protocols, monitoring and third-party verification that address these hotspots [47]. For example, this may include batch-level contaminant screening performed by accredited laboratories and digital traceability systems combined with third-party certification of environmental performance claims.

From a theoretical perspective, this study contributes to the emerging interface between environmental assessment and SBM research by demonstrating how quantified LCA outputs can be operationalised within an ecosystem-oriented business model design process. Rather than treating environmental assessment as a separate analytical exercise, the proposed approach embeds LCA hotspot information directly into business model configuration and experimentation. The SBMC structure ensures that hotspots are mapped to specific activities, resources and cost drivers, and that responsibilities and financing mechanisms are made explicit [30,56].

In the SOMMELIER case, EBME is useful because the sustainable business model cannot be implemented by a single actor. The producer organisation can coordinate the pathway, but environmental performance depends on complementary activities carried out by different actors: the processor must keep residues clean and available at the right time, the extraction operator must control energy use and process efficiency, laboratories must verify safety and bioactive quality, advisors must translate the extract into application protocols, and farmers must adopt the product at an effective dose. EBME therefore helps explain how the SBMC depends on the alignment of these interdependent roles, rather than on the optimisation of one focal firm alone [30,38,55].

Compared with the TLBMC, which separates economic, environmental and social layers, the SBMC integrates these dimensions and keeps value capture central [56]. This integration matters when environmental improvements require investment: specifying how value is captured to finance hotspot mitigation reduces the risk that sustainability remains aspirational or underfunded [27].

Three managerial implications follow. First, extraction-stage optimisation should be prioritised. Energy efficiency and renewable electricity procurement can influence multiple impact categories and stabilise operating costs, consistent with SBM archetypes focusing on efficiency and renewable [29].

Second, toxicity and critical resource hotspots require proactive management through safer chemistry screening, closed-loop handling, supplier requirements and monitoring. These actions should be embedded as capabilities (resources) rather than treated as compliance overhead, because they enable legitimacy and premium positioning [49].

Third, value capture mechanisms should be designed to fund mitigation. From an implementation standpoint, this implies reporting at least a small set of economic parameters alongside hotspot metrics – such as testing/verification cost per batch, litres per batch, and energy cost per litre – so that the feasibility of mitigation can be assessed as the pathway scales and business model assumptions are updated with operational data.

Hybrid models combining product sales with services (advisory, monitoring) and performance-based contracts can align incentives and finance continuous improvement [27,28]. For cooperatives, residue contracts should specify quality and timing and include incentives for segregation and freshness to reduce processing intensity and losses [54].

Scaling decisions (decentralised vs hub) should consider not only cost but also the capacity to mitigate hotspots. Shared hubs may justify investments in recovery infrastructure, whereas decentralised units may reduce transport and spoilage. These choices reflect the broader circular supply chain challenge of aligning technical efficiency with governance complexity [35,54].

Innovation programmes and policymakers can increase the credibility of circular bioeconomy support by requiring LCA-informed business model experimentation deliverables [24]. Funding criteria could include hotspot mitigation plans, verification strategies and periodic LCA updates using operational data, rather than prototype demonstrations alone [25].

Public support can also target enabling infrastructures (renewable energy access, water services, shared extraction hubs, accredited testing laboratories) that lower the cost of hotspot mitigation for SMEs and cooperatives [7,35]. In parallel, regulatory frameworks such as Regulation (EU) 2019/1009 can reduce market uncertainty but impose compliance costs; targeted support for conformity assessment may accelerate adoption while safeguarding safety and transparency [12].

## 6. Conclusions

This paper developed an LCA-informed procedure for sustainable business model design in circular bioeconomy pathways and applied it to the SOMMELIER case, where leek processing residues are valorised into bioactive extracts for use in lettuce cultivation. The comparative LCA shows that the alternative scenario can reduce several EF midpoint indicators, especially those related to acidification and eutrophication. At the same time, the results reveal trade-offs in toxicity-related indicators and mineral and metal resource use, confirming that residue valorisation cannot be assumed to be environmentally beneficial without a multi-indicator assessment.

The study contributes by showing how quantified LCA hotspots can be translated into business model design choices. By combining EBME and the SBMC, the analysis links environmental hotspots to operational levers, actor responsibilities and value capture mechanisms. In the SOMMELIER case, this means that extraction efficiency, renewable electricity sourcing, safer input management, traceability and laboratory verification are not ancillary activities, but core elements of the business model required to make the circular pathway credible and scalable.

For managers, cooperatives and processors, the main implication is that environmental assessment should be used early in the innovation process, not only as an ex-post evaluation tool. Hotspot information can guide investment priorities, supplier requirements, residue management protocols and customer-facing claims. In practical terms, circular bioeconomy initiatives should be designed around measurable variables such as energy use per litre of extract, minimum effective agronomic dose, batch-level testing requirements and verification costs. Linking these variables to pricing, contracts and service components can help ensure that environmental mitigation is financially supported rather than treated as an unfunded aspiration.

The study also provides indications for policymakers and innovation programmes. Pilot projects should not be assessed only on technical feasibility or the existence of a circular output, but also on whether they include hotspot mitigation plans, monitoring protocols and mechanisms for updating the LCA as the process scales. Support for shared infrastructure, accredited testing, renewable energy access and conformity assessment can reduce the cost of credible implementation for SMEs and producer organisations.

The findings should be interpreted in light of the early-stage nature of the case. The LCA relies on pilot-scale data, with limited inventory detail for some process steps and system boundaries that exclude transport, packaging and end-of-life stages. Future work should test alternative allocation and substitution assumptions, quantify uncertainty, and model industrial-scale extraction under different energy mixes and recovery efficiencies. Additional agronomic trials are also needed to confirm dose-response relationships across seasons and production contexts. Finally, integrating techno-economic assessment, business model LCA and stakeholder research would help evaluate whether the proposed model remains viable when moving from pilot experimentation to commercial implementation.
