# Supplementary material for: Evidence-based sustainable business model design for agrifood side-stream biostimulants
Source: PLoS One. 2026 Jul 8;21(7):e0343143. doi: 10.1371/journal.pone.0343143 (PMC13345246; doi:10.1371/journal.pone.0343143)
Supplement: Attachment — Submitted filename: Plus ONE - Business model of Biostimulants R1.pdf [file pone.0343143.s002.pdf]

PLOS One

Evidence-based sustainable business model design for agrifood side-stream biostimulants

--Manuscript Draft--

**Manuscript Number:**	PONE-D-26-03978R1
**Article Type:**	Research Article
**Full Title:**	Evidence-based sustainable business model design for agrifood side-stream biostimulants
**Short Title:**	Evidence-based business model design for agrifood biostimulants
**Corresponding Author:**	Federico Zilia<br>Universita degli Studi di Milano<br>Milano, ITALY
**Keywords:**	food waste; circular bioeconomy; life cycle assessment; sustainable business model
**Abstract:**	Circular bioeconomy initiatives often valorise food-processing residues into bio-based inputs, yet environmental gains depend on how technologies are implemented and governed across multiple actors. This study evaluates a pathway that converts leek processing residues into a bioactive extract used as a plant biostimulant in greenhouse lettuce production and develops an evidence-based procedure to design a sustainable business model around it. We conducted a cradle-to-gate LCA of leek extract production and a comparative LCA of lettuce cultivation for a baseline system and an alternative system applying the extract, using the Environmental Footprint (EF) 3.1 midpoint indicators. The alternative scenario improved most indicators, with consistent reductions in acidification, eutrophication and freshwater ecotoxicity, but trade-offs emerged for toxicity-related indicators and mineral/metal resource use driven by the extract supply chain. We then embedded these multi-impact hotspot signals into an ecosystem-oriented Sustainable Business Model Canvas (SBMC) and an experimentation logic, specifying who must act on each hotspot (processors, extract producers, labs, farmers) and how mitigation and verification can be financed through value capture (product-service bundles, monitoring, and conditional sustainability claims). The approach links LCA evidence to actionable business model choices, supporting credible scaling of residue-based agrifood innovations.
**Order of Authors:**	Federico Zilia
	Luca Ferraro
	Jacopo Bacenetti
	Luigi Orsi
**Response to Reviewers:**	Dear Editor, Dear Reviewers,<br>We would like to express our sincere gratitude for the time and effort you dedicated to reviewing our manuscript. Your insightful comments and constructive suggestions have been extremely valuable in improving the quality, clarity, and methodological rigor of our work. We have carefully addressed all the points raised by the Reviewers. To facilitate the reading of our revisions, we have adopted the following formatting:<br>•Our detailed responses to each comment are written in blue.<br>•The modified or added parts within the manuscript are highlighted in red (and are also clearly indicated in the "tracked changes" version of the revised manuscript).<br>We hope that these improvements meet your expectations and that the manuscript is now suitable for publication in your prestigious journal.<br>Best regards.<br><br>Reviewer 1<br><br>Dear Editor and Authors,<br>I found the manuscript timely and well aligned with PLOS ONE, especially in how it links LCA evidence to an ecosystem-oriented sustainable business model design logic. In general, the contribution is clear and well structured, but I recommend some

revisions to improve transparency and the quality of the work in general.

More precisely, I suggest to:
•Clarify and justify the extract application rate (reported as 1 L m^{-2})

Dear reviewer, thank you for your suggestion. We have updated Section 3.2 to clarify the rationale behind the chosen application rate. The dose of 1 L m^{-2} was selected as it represents a standard volume for soil-drench applications in greenhouse lettuce, ensuring an even distribution of the biostimulant within the root zone while maintaining optimal soil moisture levels for nutrient uptake.
The application rate of 1 L m^{-2} was defined to ensure a uniform distribution of the extract across the cultivated area, following standard agronomic practices for soil-applied liquid biostimulants in greenhouse conditions. This volume was calibrated to achieve sufficient soil moisture for bioactive compound uptake without causing leaching or runoff.

•Add 2 to 3 sentences specifying key background modelling choices (for example, the database version, electricity mix, main emission models) to support reproducibility.

Dear reviewer, thank you for your suggestions. We better specified these aspects in Section 3.2 "Functional units, inventory data and impact assessment"
Life Cycle Impact Assessment (LCIA) was performed using the characterisation factors provided by the Environmental Footprint (EF) 3.1 method, the impact assessment method endorsed by the European Commission [59]. The impact following impact categories were considered: climate change, acidification, particulate matter formation, freshwater ecotoxicity, freshwater, terrestrial and marine eutrophication, human toxicity (cancer and non-cancer), ozone depletion, photochemical ozone formation, fossil resource use, and mineral and metal resource use. LCA modelling was carried out using SimaPro software.
The added reference [59] is the following: Fazio S, Biganzoli F, De Laurentiis V, Zampori L, Sala S, Diaconu E. Supporting information to the characterisation factors of recommended EF Life Cycle Impact Assessment methods – Version 2, from ILCD to EF 3.0. Luxembourg: Publications Office of the European Union; 2018. https://doi.org/doi/10.2760/002447.

•In the comparative results, toxicity and mineral/metal resource use increase in the alternative scenario. You should add a short explicit explanation on it.

We agree with your point. The increase in these specific categories is linked to the "upstream" impacts of the biostimulant production (energy for extraction and lab consumables), which are absent in the baseline. We have added an explicit explanation in Section 4.1.2 to clarify this trade-off.
This increase in human toxicity (cancer) and mineral resource use is primarily due to the additional energy-intensive steps and the specific laboratory materials required for the production of the leek extract, which are not required in the traditional baseline scenario.

•Tables would benefit from quick consistency edits. I suggest you harmonise decimal separators (e.g., 0.11 vs 0,11) and scientific notation formatting across Table 1 and Table 2.

We apologize for these inconsistencies, which resulted from a formatting error during the final assembly of the document. We have now fully revised Table 1 and Table 2 to ensure that decimal separators (using the point "." as the standard) and scientific notation formatting are consistent throughout the manuscript.

•I suggest you to better explain the sentence in lines 515-517 when you said: "model should "embed protocols, monitoring and third-party verification". Please add one sentence that names two concrete instruments (so readers immediately see what you mean in practice).

Thank you for the suggestion. We have clarified the sentence by adding concrete examples of operational instruments, such as batch-level contaminant screening conducted by accredited laboratories and digital traceability protocols with third-party

certification of environmental claims.

•I recommend you a careful spell-check of the manuscript. Indeed, there are some minor typos and grammatical phrasing that could be smoothed out.

Dear reviewer, thank you for this reminder. We corrected some minor typos.

•I would suggest expanding the Conclusions to more explicitly address future perspectives, particularly regarding the managerial implications derived from the study.

Dear reviewer, thank you for your comment. Following your suggestion, we expanded the Conclusions section to more clearly articulate the managerial implications and future perspectives of the proposed LCA-business model integration framework, particularly regarding its role as a decision-support tool for scaling circular bioeconomy innovations.

•With regard to LCA the selection of the LCIA method should be justified and, if possible, a comparison with previously carried out studies should be introduced into the discussion. If the comparison is not possible due to the lack of studies this aspect should be better underline.

Dear reviewer, thanks for the suggestion. Regarding the LCIA method we have specified in the manuscript that it was selected because it is the one endorsed at European level by the European Commission. About the comparison with previously carried out studies, following your suggestion we have specified that a comparison cannot be drawn because no other LCA studies were carried out on the same topic. In detail, the following sentences were introduced:
The results of LCA study, for both the considered functional units, cannot be compared with other literature data because no previous LCA studies were carried out on the production of leek extract and its application during lettuce cultivation. Despite this, the achieved results allow the identification of the main contributors to the environmental impact and represent a starting point for a further optimisation of the process.

With these moderate revisions and formatting fixes, the paper will be stronger and easier to interpret.
Thank you!

Reviewer 2

•Writing style is acceptable.

Dear reviewer, thank you.
In any case, we have proofread the English to make stylistic improvements, minor corrections, and to fix some typos.

•However, there must be a clearer link between LCA and the Business model. Currently, the relation is interpretive. The article is presented as a practice management reflection. It requires more discussion on broader areas (how is this method transferable? Is this replicable beyond this case?).

Dear reviewer, we agree with your comments and have strengthened both the methodological bridge and the generalisability discussion. In the revised manuscript we now formalise a transparent “hotspot-to-canvas” protocol that translates quantified EF 3.1 LCA hotspot drivers into specific SBMC design levers, actor responsibilities, measurable decision variables, and value-capture/governance mechanisms. Moreover, we explicitly described the step-by-step procedure in Section 3.3 (S1-S5) to reduce interpretive inference and we added a dedicated paragraph in the Discussion clarifying transferability and replicability conditions (what data are needed, which variables should be measured and updated when scaling, and how the protocol can be repeated for other residue-to-input pathways). These additions make the LCA-business model linkage operational rather than reflective and clarify how the approach can be replicated beyond this single case.

•The contribution must be also clearer stated. Be sure to mention what any foreign

phrase means, there were a couple of Italian words without their equivalent translation in English.

Dear reviewer, thank you for this helpful suggestion. We have strengthened the contribution statement in the Introduction and clarified the theoretical contribution of the study. In particular, we now explicitly articulate three contributions: (i) demonstrating how multi-indicator LCA results can inform sustainable business model design in circular bioeconomy contexts; (ii) proposing a transparent “hotspot-to-canvas” protocol linking LCA hotspot drivers to SBMC elements and actor responsibilities; and (iii) illustrating how environmental evidence can support ecosystem-level business model experimentation and governance.
In addition, we revised the manuscript to ensure that all foreign expressions are clearly explained. Specifically, the Italian expression “prima gamma evoluta” is now translated as “fresh-cut, minimally processed ready-to-cook vegetables”. These changes improve clarity and accessibility for an international readership.

•Explain further the sensitivity analysis in Section 4.

We appreciate the comment. In Section 4.3, we performed a scenario-based sensitivity analysis by comparing two different implementation pathways: Scenario S1 (decentralized, near-source extraction) and Scenario S2 (centralized, shared-hub model). These scenarios serve to test how the environmental hotspots and the business model’s viability are sensitive to changes in scale, logistics, and infrastructure efficiency. We have now added a sentence at the beginning of Section 4.3 to explicitly define this comparison as a scenario-based sensitivity assessment.

•Report Toxicities using a benchmark and use other metrics.

Thank you for the comment and suggestion. Although the alternative scenario increases toxicity indicators, due to the additional process of the system, the absolute values remain in the range usually reported for greenhouse vegetable productions, also the contribution analysis indicates that the contribution of these impacts represents a small fraction of the environmental burden per kg of lettuce.
The Alternative tends to show lower environmental burdens across most impact categories, with the exception of cancer-related toxicity and mineral resource use, where the baseline scenario performs better. This increase in human toxicity (cancer) and mineral resource use is primarily due to the additional energy-intensive steps and the specific laboratory materials required for the production of the leek extract, which are not required in the traditional baseline scenario. These findings highlight trade-offs between different impact categories, with neither scenario showing a clear overall dominance.

•If they claim to bridge a gap or address a need, they need to make what they are doing more formally stated. Or tone down the claims.

Thank you for this suggestion. We have revised the Introduction to more clearly and formally articulate the research gap addressed by the study. In particular, we clarify that while Life Cycle Assessment is widely used to evaluate environmental impacts of circular bioeconomy innovations, and sustainable business model frameworks provide tools to analyse value creation and governance mechanisms, the methodological integration between these two perspectives remains underdeveloped. The revised manuscript now explicitly states this gap and clarifies how the proposed “hotspot-to-canvas” protocol contributes to bridging it. In addition, we refined the contribution statement in the Discussion section to more clearly position the study within the literature on sustainable business model innovation and environmental assessment.

•The environmental comparison implicitly assumes substitution effects, but the manuscript does not quantify changes in input use. Note: Net Welfare and Environmental performance depend on substitution rather than addition.

We agree with your observation. The environmental benefit of any bio-based input is indeed dependent on its ability to substitute conventional burdens. We have added a clarifying statement at the end of Section 4.1.3 to explicitly address this. We highlight that the “Alternative Scenario” is modelled on a nutrient-efficiency logic where the

biostimulant acts as a substitute for potential environmental impacts of traditional intensive practices, and that a net gain is only achieved if this substitution effect is realized.
In this context, it should be noted that the environmental comparison implicitly assumes a substitution logic. The application of the leek extract is intended to improve nutrient use efficiency, theoretically allowing for a reduction in conventional inputs. The net environmental performance of the alternative scenario is therefore contingent on this substitution effect; without a corresponding gain in system efficiency or a reduction in synthetic fertilizers, the biostimulant would represent an additional environmental load rather than a sustainable transition.

•The business model section mentions revenue streams and mitigation investments but doesn't include price estimates. Without a basic economic analysis it is difficult to asses whether the proposed mitigation strategy is economically feasible. Some quantitative linkage between environmental hotspots and economic incentives would strengthen the analysis.

We agree that the previous version discussed mitigation investments and revenue streams without providing a sufficiently explicit economic linkage. In the revised manuscript we add a short, narrative "economic feasibility check" in the business model results section, clarifying how hotspot mitigation can be operationalised through allocable cost units (e.g., € per batch for QA/QC and verification; kWh per litre for energy) and how these can be transparently translated into a per-litre verification-and-mitigation component for the verified sustainability tier. We also refine Table 3 to include this operational pricing logic and to clarify which measurable economic parameters should be tracked during scaling. Because the SOMMELIER pathway is currently implemented at a pilot stage, providing precise price estimates or a full techno-economic assessment would be premature. For this reason, the revised manuscript focuses on defining the cost drivers and the accounting logic through which mitigation efforts could be financed, rather than presenting definitive market prices. While this is not a full techno-economic assessment, it provides a transparent quantitative bridge between environmental hotspots and economic incentives.

•Very importantly, for a modeling paper: there is no model. I would prefer to have a visualization of what the multiple models being employed look like. And more importantly how the outputs and variables of one section are influencing the others as well as the interaction between them.

Dear reviewer, thank you for this important observation. We agree that, in the previous version, the modelling logic underlying the paper was described textually but not sufficiently visualised, which made the interaction between the analytical components less explicit than intended. To address this point, we have added a new figure in the Methods section (new Figure 1) that presents the integrated modelling framework adopted in the study.
The new figure makes explicit the sequential and translational logic connecting the different components of the paper. In particular, it shows how the LCA stage provides quantified environmental outputs in the form of EF 3.1 hotspot indicators and contribution signals; how these outputs are then processed through a hotspot-to-variable translation protocol; and how the resulting decision-relevant variables are subsequently incorporated into the sustainable business model design stage through the SBMC. In this way, the figure clarifies not only what the different modelling components are, but also how the outputs of one stage become inputs for the next one.
We have also revised Section 3.3 to explain this logic more explicitly in the text. The revised manuscript now clarifies that the study combines two complementary modelling perspectives - environmental modelling through LCA and strategic design through the SBMC - and that their interaction is operationalised through a structured five-step translation procedure. This addition was intended precisely to address the reviewer's concern regarding how variables and outputs from one section influence the others, and to make the overall architecture of the paper more transparent and easier to interpret.
Importantly, our intention is not to claim that the LCA and the business model framework are merged into a single pre-existing formal model. Rather, the contribution of the paper lies in proposing and visualising an integrated modelling framework in

Powered by Editorial Manager® and ProduXion Manager® from Aries Systems Corporation

	which quantified LCA hotspot signals are systematically translated into business model design variables, actor responsibilities, and value capture mechanisms. We believe that this addition substantially strengthens the methodological clarity of the paper.
Additional Information:	
**Question**	**Response**
**Financial Disclosure**<br><br>Enter a financial disclosure statement that describes the sources of funding for the work included in this submission. Review the submission guidelines for detailed requirements. View published research articles from *PLOS ONE* for specific examples.<br><br>This statement is required for submission and **will appear in the published article** if the submission is accepted. Please make sure it is accurate.<br><br>**Funded studies**<br>Enter a statement with the following details:<br>• Initials of the authors who received each award<br>• Grant numbers awarded to each author<br>• The full name of each funder<br>• URL of each funder website<br>• Did the sponsors or funders play any role in the study design, data collection and analysis, decision to publish, or preparation of the manuscript?<br><br>Did you receive funding for this work?	Yes
Please add funding details.<br>as follow-up to "**Financial Disclosure**<br><br>Enter a financial disclosure statement that describes the sources of funding for the work included in this submission. Review the submission guidelines for detailed requirements. View published research articles from *PLOS ONE* for specific examples.<br><br>This statement is required for submission and **will appear in the published article** if the submission is accepted. Please make	This work was carried out within the SOMMELIER (Scarti pOrro priMa gaMma EvoLuta bIoattivi pEr l'agRicoltura) project, co-funded by the European Agricultural Fund for Rural Development (EAFRD/FEASR) under Lombardy Region Rural Development Programme (PSR) 2014-2020, Operation 16.2.01 "Pilot projects and development of innovation". The project was coordinated by AOP UNOLOMBARDIA, in collaboration with the University of Milan and Ortonatura S.A.C.A. R.L., and supported by Regione Lombardia (DG Agriculture).

sure it is accurate.<br><br>**Funded studies**<br>Enter a statement with the following details:<br>• Initials of the authors who received each award<br>• Grant numbers awarded to each author<br>• The full name of each funder<br>• URL of each funder website<br>• Did the sponsors or funders play any role in the study design, data collection and analysis, decision to publish, or preparation of the manuscript?<br><br>Did you receive funding for this work?"	
Please select the country of your main research funder (please select carefully as in some cases this is used in fee calculation).<br>as follow-up to "**Financial Disclosure**<br><br>Enter a financial disclosure statement that describes the sources of funding for the work included in this submission. Review the submission guidelines for detailed requirements. View published research articles from *PLOS ONE* for specific examples.<br><br>This statement is required for submission and **will appear in the published article** if the submission is accepted. Please make sure it is accurate.<br><br>**Funded studies**<br>Enter a statement with the following details:<br>• Initials of the authors who received each award<br>• Grant numbers awarded to each author<br>• The full name of each funder<br>• URL of each funder website<br>• Did the sponsors or funders play any role in the study design, data collection and analysis, decision to publish, or preparation of the manuscript?<br><br>Did you receive funding for this work?"	ITALY - IT

Powered by Editorial Manager® and ProduXion Manager® from Aries Systems Corporation

**Competing Interests**<br><br>Use the instructions below to enter a competing interest statement for this submission. On behalf of all authors, disclose any competing interests that could be perceived to bias this work—acknowledging all financial support and any other relevant financial or non-financial competing interests.<br><br>This statement is required for submission and **will appear in the published article** if the submission is accepted. Please make sure it is accurate and that any funding sources listed in your Funding Information later in the submission form are also declared in your Financial Disclosure statement.<br><br>View published research articles from *PLOS ONE* for specific examples.<br><br>**NO authors have competing interests**<br><br>Enter: *The authors have declared that no competing interests exist.*<br><br>**Authors with competing interests**<br><br>Enter competing interest details beginning with this statement:<br><br>*I have read the journal's policy and the authors of this manuscript have the following competing interests: [insert competing interests here]*<br><br>* typeset	Federico Zilia serves as an Academic Editor for PLOS ONE. To avoid any potential conflict of interest, we request that he is not involved in the handling of this submission. This does not alter our adherence to PLOS ONE policies on sharing data and materials.
**Ethics Statement**<br><br>Enter an ethics statement for this submission. This statement is required if the study involved:<br><br>• Human participants<br>• Human specimens or tissue<br>• Vertebrate animals or cephalopods	Primary data were collected through voluntary interviews with farm operators using a structured questionnaire for the purpose of compiling life cycle assessment (LCA) inventory data. The interviews were conducted solely to collect descriptive and contextual information on production practices and input use. Participation was entirely voluntary and involved no interventions, treatments, or manipulation of participants or their activities. Prior to participation, adult respondents received clear information about the objectives of the study, the nature of the questions, and the intended use of the data for research purposes, and they provided formal informed consent to answer the questionnaire. No minors were involved. No directly identifying personal data were

Powered by Editorial Manager® and ProduXion Manager® from Aries Systems Corporation

• Vertebrate embryos or tissues<br>• Field research<br><br>Write "N/A" if the submission does not require an ethics statement.<br><br>General guidance is provided below. Consult the submission guidelines for detailed instructions. **Make sure that all information entered here is included in the Methods section of the manuscript.**	collected, and all responses were processed and reported exclusively in anonymized and/or aggregated form. The study protocol was deemed not to require formal ethics committee approval in accordance with applicable institutional and national regulations governing anonymous, non-interventional survey-based research.

Powered by Editorial Manager® and ProduXion Manager® from Aries Systems Corporation

**Format for specific study types**<br><br>**Human Subject Research (involving human participants and/or tissue)**<br>• Give the name of the institutional review board or ethics committee that approved the study<br>• Include the approval number and/or a statement indicating approval of this research<br>• Indicate the form of consent obtained (written/oral) or the reason that consent was not obtained (e.g. the data were analyzed anonymously)<br><br>**Animal Research (involving vertebrate animals, embryos or tissues)**<br>• Provide the name of the Institutional Animal Care and Use Committee (IACUC) or other relevant ethics board that reviewed the study protocol, and indicate whether they approved this research or granted a formal waiver of ethical approval<br>• Include an approval number if one was obtained<br>• If the study involved *non-human primates*, add *additional details* about animal welfare and steps taken to ameliorate suffering<br>• If anesthesia, euthanasia, or any kind of animal sacrifice is part of the study, include briefly which substances and/or methods were applied<br><br>**Field Research**<br><br>Include the following details if this study involves the collection of plant, animal, or other materials from a natural setting:<br>• Field permit number<br>• Name of the institution or relevant body that granted permission	
**Data Availability**<br><br>Authors are required to make all data underlying the findings described fully available, without restriction, and from the time of publication. PLOS allows rare exceptions to address legal and ethical concerns. See the PLOS Data Policy and FAQ for detailed information.	Yes - all data are fully available without restriction

A Data Availability Statement describing where the data can be found is required at submission. Your answers to this question constitute the Data Availability Statement and **will be published in the article**, if accepted.<br><br>**Important:** Stating 'data available on request from the author' is not sufficient. If your data are only available upon request, select 'No' for the first question and explain your exceptional situation in the text box.<br><br>Do the authors confirm that all data underlying the findings described in their manuscript are fully available without restriction?	
**Describe where the data may be found in full sentences. If you are copying our sample text, replace any instances of XXX with the appropriate details.**<br><br>• If the data are **held or will be held in a public repository**, include URLs, accession numbers or DOIs. If this information will only be available after acceptance, indicate this by ticking the box below. For example: *All XXX files are available from the XXX database (accession number(s) XXX, XXX.).*<br>• If the data are all contained **within the manuscript and/or Supporting Information files**, enter the following: *All relevant data are within the manuscript and its Supporting Information files.*<br>• If neither of these applies but you are able to provide **details of access elsewhere**, with or without limitations, please do so. For example:<br><br>*Data cannot be shared publicly because of [XXX]. Data are available from the XXX Institutional Data Access / Ethics Committee (contact via XXX) for researchers who meet the criteria for access to confidential data.*<br><br>*The data underlying the results presented in the study are available from (include the name of the third party*	The data supporting the findings of this study are available from the corresponding author upon reasonable request.

and contact information or URL).<br>• This text is appropriate if the data are owned by a third party and authors do not have permission to share the data.<br><br>* typeset	
Additional data availability information:	Tick here if your circumstances are not covered by the questions above and you need the journal's help to make your data available.

Powered by Editorial Manager® and ProduXion Manager® from Aries Systems Corporation

Manuscript_revised
Click here to access/download;Manuscript;Manuscript_revised.docx

Evidence-based sustainable business model design for agrifood side-stream biostimulants

Federico Zilia[1,*], Luca Ferraro[1], Jacopo Bacenetti[1], Luigi Orsi[1]

[1] Department of Environmental Science and Policy, University of Milan, Milan, Italy

*Corresponding author: federico.zilia@unimi.it

Abstract

Circular bioeconomy initiatives often valorise food-processing residues into bio-based inputs, yet environmental gains depend on how technologies are implemented and governed across multiple actors. This study evaluates a pathway that converts leek processing residues into a bioactive extract used as a plant biostimulant in greenhouse lettuce production and develops an evidence-based procedure to design a sustainable business model around it. We conducted a cradle-to-gate LCA of leek extract production and a comparative LCA of lettuce cultivation for a baseline system and an alternative system applying the extract, using the Environmental Footprint (EF) 3.1 midpoint indicators. The alternative scenario improved most indicators, with consistent reductions in acidification, eutrophication and freshwater ecotoxicity, but trade-offs emerged for toxicity-related indicators and mineral/metal resource use driven by the extract supply chain. We then embedded these multi-impact hotspot signals into an ecosystem-oriented Sustainable Business Model Canvas (SBMC) and an experimentation logic, specifying who must act on each hotspot (processors, extract producers, labs, farmers) and how mitigation and verification can be financed through value capture (product-service bundles, monitoring, and conditional sustainability claims). The approach links LCA evidence to actionable business model choices, supporting credible scaling of residue-based agrifood innovations.

Keywords: food waste; circular bioeconomy; life cycle assessment; sustainable business model.

1. Introduction

Food loss and waste (FLW) is widely recognised as a major sustainability challenge across agri-food systems, with direct implications for climate change, land and water use, eutrophication and biodiversity loss [1–3] At the same time, processing and distribution chains are reconfigured by consumer demand for convenience and safety, expanding fresh-cut and minimally processed 'ready-to-cook' products; these chains concentrate residues spatially and temporally, creating both a waste management burden and a circular bioeconomy opportunity [4,5].

In the European Union, circular economy (CE) policy and bioeconomy strategies increasingly encourage higher-value utilisation of side-streams by applying cascading principles and avoiding low-value downcycling [6,7]. However, scholars warn that circular strategies can generate rebound effects or burden shifting if the energy and material intensity of valorisation outweighs avoided burdens, or if environmental improvements are claimed on narrow indicators such as climate while ignoring toxicity, water scarcity or critical materials [8,9]. These concerns underline the need for multi-impact assessment and transparent accounting when designing circular innovations [10,11].

Plant biostimulants represent a fast-growing category of bio-based agricultural inputs. EU Regulation (EU) 2019/1009 provides a harmonised market framework and a functional definition of biostimulants as products that stimulate plant nutrition processes to improve nutrient use efficiency, stress tolerance, quality traits or nutrient availability, independently of nutrient content [12]. Scientific reviews highlight that biostimulants span diverse categories, including microbial inoculants, humic substances, protein hydrolysates and amino acids, and botanical extracts [13–16]. While biostimulants are often positioned as sustainable alternatives, their net environmental performance is contingent on production pathways and on the extent to which they substitute conventional inputs or stabilise yields under stress [16,17].

Against this background, the SOMMELIER project, led by the University of Milan, develops a circular pathway to valorise leek (*Allium porrum L.*) residues generated in "prima gamma evoluta" processing (an Italian term referring to fresh-cut, minimally processed ready-to-cook vegetables).

In the focal supply chain, leek cultivation covers roughly 25 ha out of 65 ha managed by the producer organisation; processing generates a substantial non-edible fraction (roots, leaves and green bulb parts). While 10-15% of edible fractions are reused, an estimated 30-40% remains under-valorised, corresponding to approximately 225-300 metric tons per year. The project employs pilot-scale Naviglio extraction equipment to obtain bioactive extracts for agricultural use, initially demonstrated in lettuce production.

Life cycle assessment (LCA) is well established for quantifying environmental impacts of products and technologies [18–20]. In circular bioeconomy contexts, LCA is increasingly used to identify hotspots and to compare design options under transparent assumptions [5,21]. Early applications of LCA to horticultural crops have already highlighted the relevance of leek as a case study for assessing environmental performance in agriculture, comparing organic and conventional production systems and identifying fertilisation and energy use as major environmental hotspots [22]. Despite this tool can provides a relevant support for decision making, notably in waste management [23], the LCA alone does not specify how value is created, delivered and captured across multiple actors. Moreover, it does not represent how costs and benefits should be shared, or how environmental hotspots can be financed and governed during scaling [24,25]. Conversely, sustainable business model frameworks provide conceptual tools for designing multi-dimensional value propositions but are often criticised for lacking quantified environmental evidence to guide design priorities [26–28].

~~This paper primarily targets sustainability and innovation scholars interested in business model design for circular bioeconomy pathways, while using LCA as an enabling decision-support tool rather than as an end in itself.~~

Despite growing research on circular bioeconomy and sustainable business models, the integration of multi-indicator environmental assessment with business model design remains limited. In particular, existing studies often apply LCA to evaluate environmental impacts, while

sustainable business model frameworks focus on value creation and governance mechanisms, but the methodological link between these two analytical perspectives remains underdeveloped.

Accordingly, this paper responds to this gap by integrating LCA evidence with sustainable business model innovation for circular side-stream valorisation. This study contributes to the literature in three main ways. First, it provides an empirical demonstration of how multi-indicator LCA results can be translated into actionable business model design choices in circular bioeconomy contexts. Second, it proposes a transparent "hotspot-to-canvas" translation protocol that systematically links environmental hotspot drivers to specific Sustainable Business Model Canvas (SBMC) elements, actor responsibilities, and governance mechanisms. Third, it illustrates how environmental assessment can support ecosystem-level business model experimentation, helping actors design financeable mitigation strategies for environmental trade-offs rather than relying on descriptive sustainability claims. Indeed, building on recent work on business model experimentation and Ecologies of Business Models (EBME), we combine this tool with the Sustainable Business Model Canvas (SBMC) to translate quantified LCA hotspots into explicit business model design choices and value capture mechanisms [29–31]. To reduce interpretive inference and make the integration replicable, we apply a transparent "hotspot-to-canvas" translation protocol. Specifically, we *(i)* extract hotspot signals from the EF 3.1 midpoint results and contribution analysis (i.e., impact categories with material differences and their driving processes), *(ii)* assign each hotspot to the responsible SBMC block(s) (activities/resources/costs/partners) and to a concrete intervention hypothesis, *(iii)* specify a measurable implementation variable for each intervention (e.g., kWh L^{-1}, renewable electricity share, solvent/water recirculation rate, contaminant thresholds), and *(iv)* link these variables to governance and value-capture mechanisms (contracts, monitoring services, conditional sustainability claims) so that mitigation is financeable. This protocol is intended to be transferable: any residue-to-input pathway can be analysed by repeating steps once a process-based Life Cycle Inventory (LCI) is available and the downstream use scenario is defined.

The paper addresses three research questions: *(RQ1)* What are the main environmental hotspots and trade-offs of producing leek extract and applying it in lettuce cultivation compared with a baseline scenario? *(RQ2)* How can LCA evidence be operationalised into sustainable business model design using SBMC in a multi-actor ecosystem? *(RQ3)* What managerial, governance and policy implications follow for scaling circular side-stream valorisation credibly without burden shifting?

The manuscript is organized as follows: Section 2 reviews the literature on food waste valorisation, biostimulants, life cycle assessment and sustainable business models for circular bioeconomy pathways. Section 3 describes the case study, the LCA modelling approach and the sustainable business model design procedure. Section 4 reports the LCA and sustainable business model results. Section 5 discusses implications, limitations and future research directions, and Section 6 concludes.

2. Background and related literature

2.1 Circular bioeconomy strategies for agri-food side-streams

FLW valorisation is located at the interface of CE, industrial ecology and the bioeconomy [7,32]. While CE is widely promoted in policy and practice [6,33], academic work emphasises that CE is a contested and multi-interpretation concept with diverse definitions and priorities [9,10]. These conceptual ambiguities matter because they shape what organisations measure and legitimise when implementing circular initiatives [11,34].

In agri-food, cascading and biorefinery perspectives argue that side-streams should be directed to applications with higher functionality and longer value retention, before considering energy recovery [4,5]. Reviews of food manufacturing waste valorisation document options ranging from functional ingredients and biomaterials to composting and anaerobic digestion, with feasibility constrained by contamination, seasonality and logistics [4,5]. Industrial symbiosis scholarship

highlights that the value of side-streams depends on local actor networks and infrastructural proximity, suggesting that circular pathways are ‘place-based’ and governance-intensive [35,36].

However, CE scholars stress the risk of the ‘circular economy rebound’: closing loops can increase total throughput or shift impacts elsewhere, for example through increased processing or transport intensity [8]. Accordingly, circular strategies should be evaluated through multi-impact assessment rather than single-indicator claims [9]. Moreover, governance and contract design are critical because residue quality, segregation and timing strongly influence downstream process efficiency and environmental performance [37,38].

Plant biostimulants are increasingly framed as enabling more resource-efficient and resilient agriculture, potentially contributing to nutrient management, stress tolerance and yield stability [13–15]. The literature emphasises that biostimulants should be defined by their functions rather than solely by nutrient content, recognising that complex mixtures can modulate plant physiological processes [13,16]. Empirical studies report diverse effects across crops and contexts, including improved root development, enhanced nutrient uptake and stress mitigation [16,17].

Yet biostimulants also raise sustainability questions. First, the environmental impacts of producing bio-based inputs depend on extraction pathways, solvents, energy intensity and upstream feedstock logistics [5,21]. Second, agronomic efficacy must be robust across conditions to deliver net system-level benefits; otherwise, additional inputs may add impacts without sufficient yield or substitution gains [16]. Third, regulatory and market legitimacy depend on safety, traceability and claim verification, particularly where complex biological matrices can contain contaminants [12].

Consequently, a sustainability-oriented innovation approach should couple agronomic and technological testing with robust environmental assessment and with business model design that allocates responsibilities, costs and benefits across the actor network [28,29].

2.2 LCA approaches

LCA provides a systematic framework to quantify environmental impacts across life cycle stages, typically in line with ISO 14040/14044 [18–20]. In side-stream valorisation, methodological choices are salient: whether residues are treated as wastes carrying no upstream burdens, or as by-products requiring allocation; how co-products are handled; and whether avoided products should be modelled via system expansion or substitution [39,40]. Because these choices can change results and their interpretation, early-stage studies often adopt attributional approaches with explicit assumptions and sensitivity logic [20,41].

The Environmental Footprint (EF) methods offer a harmonised midpoint indicator set widely used in European contexts [42]. EF indicators enable multi-criteria hotspot analysis, including climate change, acidification, eutrophication, particulate matter, photochemical ozone formation, ozone depletion, human toxicity, freshwater ecotoxicity, resource use (fossil; minerals/metals) and water deprivation [42]. For bio-based extraction processes, toxicity and mineral/metal indicators can be decision-relevant because solvents, catalysts and equipment materials may dominate these categories even when climate performance appears favourable [42,43].

The role of LCA in innovation is increasingly conceptualised as decision support: hotspot identification can guide iterative redesign and reduce the risk of 'environmental lock-in' as technologies scale [24]. However, translating hotspots into operational changes requires organisational and inter-organisational mechanisms: who invests, who benefits and how improvements are verified [25].

2.3 Sustainable business models for circularity

SBM research extends conventional business model thinking by integrating environmental and social value creation alongside economic value capture [44,45]. A business model is commonly defined as the system of activities through which an organisation creates and captures value [46,47],

while sustainability-oriented work emphasises that value should be created for a broader set of stakeholders and within ecological limits [28,48,49].

Bocken et al. [29] synthesise SBM archetypes, including ‘create value from waste’, ‘maximise material and energy efficiency’ and ‘substitute with renewables and natural processes’. Circular economy business model pattern research further classifies recurring configurations and highlights barriers for SMEs, including finance, market demand and collaboration challenges [35,40].

Canvases such as the Business Model Canvas proposed by Osterwalder and Pigneur [50] provide an accessible structure for describing business model elements, and sustainability extensions such as the Triple Layered Business Model Canvas (TLBMC) add environmental and social layers [51]. The TLBMC has been valuable for visualising sustainability considerations and supporting ideation, particularly for single organisations or product-service offerings [51–53].

However, circular bioeconomy innovations frequently operate across multiple firms and institutions. Innovation ecosystem research and circular business model studies highlight that successful circularity requires complementary business models, coordination among actors and shared infrastructure [38,54]. The ‘ecologies of business model experimentation’ (EBME) perspective frames business model innovation as experimentation within a system of interacting business models, where synergies and trade-offs must be understood and governed [30,55]. This perspective is particularly suitable for producer-organisation-led projects such as SOMMELIER.

To integrate LCA evidence into business model design, a framework must move beyond listing impacts and instead specify actionable levers, responsibilities and financing mechanisms. While the TLBMC provides a useful layered representation of environmental and social dimensions, applications can remain descriptive and centred on a single focal organisation, with less emphasis on ecosystem coordination and on how environmental improvements are funded through value capture [27,51].

The SBMC proposed by Bocken [56] builds on the logic of value creation, delivery and capture while embedding a Profit-People-Planet value proposition at the core. It is compatible with

stakeholder-oriented sustainability value creation [49] and supports an ecosystem interpretation: the canvas can represent a focal proposition that depends on complementary partner models and governance arrangements [30,38].

Critically, SBMC blocks (key activities, resources, cost structure) provide a direct bridge to LCA hotspots because hotspots are typically associated with specific activities and resource uses [24]. Therefore, in this paper the SBMC is used as the primary business model representation, while the TLBMC is treated as a comparator motivating a move from layered description to ecosystem- and experimentation-oriented design [30,51].

A growing literature seeks to operationalise environmental assessment at the business model level. BM-LCA approaches conceptualise the business model as the object of analysis and compare environmental impacts per unit of economic value, enabling decisions about business configurations [25]. Other strands integrate LCA into early-stage experimentation, emphasising that environmental impacts should be measured and forecasted during pilots to avoid lock-in to high-impact designs [54,55].

Despite these advances, there remains a need for case-based demonstrations that use standard product-focused LCA outputs as inputs to ecosystem-oriented SBM design, producing explicit, financeable redesign levers and governance proposals [26,27]. This work addresses this gap by linking EF midpoint hotspot analysis to SBMC blocks and by articulating how value capture can fund mitigation of trade-offs.

3. Materials and methods

3.1 Study design and system modelling

The environmental performance of a leek-derived biostimulant extract and its application in greenhouse lettuce cultivation was assessed through Life Cycle Assessment (LCA) in accordance with ISO 14040 and ISO 14044 standards [57]. The study was designed as a comparative

assessment of two lettuce production systems: (i) a conventional cultivation scenario (baseline) and (ii) an alternative scenario where the leek-based extract was applied during crop management.

System boundaries were defined according to a cradle-to-gate perspective (resource extraction to factory gate [58]). For lettuce cultivation, the model included upstream production of agricultural inputs (fertilisers, plant protection products, seeds, electricity and fuels), crop establishment, fertilisation, irrigation, crop management, harvesting and on-farm transport, as well as direct emissions to air, soil and water associated with agricultural practices. For the leek extract, the system included collection of leek residues, washing, extraction and thermal treatment steps (including hydrodistillation), and the generation of solid and liquid co-products. Infrastructure, packaging and end-of-life stages were excluded from the analysis.

The environmental burdens associated with extract production were integrated into the alternative lettuce scenario using the application rate adopted in the experimental trials. Impacts related to the production of 1 L of extract were linked to lettuce cultivation by accounting for cultivated area and corresponding crop yield, allowing results to be expressed consistently per kilogram of fresh lettuce.

3.2 Functional units, inventory data and impact assessment

Two functional units (FUs) were defined to reflect the dual objective of the study. The first FU (FU1) was defined as 1 L of leek extract to quantify impacts associated with the production of the biostimulant. The second FU (FU2) was defined as 1 kg of fresh lettuce at the farm gate and represents the reference unit for comparing baseline and alternative cultivation systems.

The LCI was developed using a combination of primary and secondary data. Primary data were collected through direct interviews with farmers using specifically designed questionnaires and from experimental activities, capturing farming operations and the production factors used. The extract application rate was 1 L m^{-2}, and lettuce yields were 1.16 kg m^{-2} for the baseline scenario and 1.33 kg m^{-2} for the alternative scenario with leek extract. The application rate of 1 L m^{-2} was

defined to ensure a uniform distribution of the extract across the cultivated area, following standard agronomic practices for soil-applied liquid biostimulants in greenhouse conditions. This volume was calibrated to achieve sufficient soil moisture for bioactive compound uptake without causing leaching or runoff.

Secondary data were used to model upstream production of agricultural inputs, energy supply, and emissions related to fertiliser use and nutrient cycles, based on established modelling approaches and available databases (e.g., Ecoinvent).

Life Cycle Impact Assessment (LCIA) was performed using the characterisation factors provided by the Environmental Footprint (EF) 3.1 method, the impact assessment method endorsed by the European Commission [59]. The impact following impact categories were considered: climate change, acidification, particulate matter formation, freshwater ecotoxicity, freshwater, terrestrial and marine eutrophication, human toxicity (cancer and non-cancer), ozone depletion, photochemical ozone formation, fossil resource use, and mineral and metal resource use. LCA modelling was carried out using SimaPro software.

3.3 Sustainable business model design

We combine the Ecologies of Business Model Experimentation perspective with Sustainable Business Model Canvas. EBME frames business model innovation as experimentation within a system of interacting business models and is structured here as: (1) sustainability aims; (2) key dependencies and actor mapping; (3) synergies, trade-offs and risks; and (4) design and experimentation priorities.

The SBMC is then used to represent the business model hypothesis in a structured way, integrating value creation, delivery and capture with a Profit-People-Planet value proposition [29,56]. ~~The SBMC is selected over the Triple Layered Business Model Canvas (TLBMC) because *(i)* it supports an ecosystem interpretation suitable for circular supply chains, *(ii)* it ties~~

~~environmental levers to activities, resources and value capture (i.e., financing), and *(iii)* it aligns with stakeholder-oriented sustainability value creation [38,49,51].~~

To clarify the methodological architecture and the interaction between the analytical components used in this study, Fig 1 presents the integrated modelling framework adopted in the research. The framework links environmental modelling and business model design through a structured translation protocol. In this approach, environmental hotspot signals generated by the LCA analysis are translated into operational design variables that inform sustainable business model configuration.

To operationalise integration, we map LCA hotspots to SBMC blocks using a transparent translation logic: each hotspot is associated with one or more intervention hypotheses (e.g., renewable electricity procurement to reduce fossil-related impacts; safer chemistry and closed-loop handling to mitigate toxicity; procurement and equipment durability strategies to address mineral/metal depletion). These hypotheses are incorporated into SBMC blocks and linked to value capture mechanisms that can fund mitigation, consistent with calls to align sustainability performance with economic incentives [27,28].

Operationally, the mapping was performed in five steps: (S1) select a hotspot set by screening EF categories for (a) relevant absolute magnitude for the functional unit and/or (b) meaningful baseline-alternative differences, and confirm drivers via contribution analysis; (S2) trace each hotspot back to the life-cycle stage and inventory items/processes responsible (e.g., electricity, auxiliary materials, field emissions); (S3) translate each driver into one or more business model design levers, and allocate responsibility to the actor(s) controlling that driver (processor, extractor, lab, farm, distributor); (S4) define a measurable decision variable and monitoring approach for each lever (metering, batch QA/QC, third-party verification); and (S5) connect each lever to a financing and governance mechanism within SBMC (cost items, revenue streams, contractual clauses, service components).

Fig 1 visualises this translation logic by showing how quantified environmental signals generated in the LCA stage are progressively transformed into actionable business model variables and design decisions within the SBMC. Reporting these steps makes the approach repeatable for other crops and residue streams, while allowing the specific levers and responsible actors to vary with local ecosystem conditions.

[**Fig 1.** Integrated LCA-SBM framework.]

3.4 Data quality, uncertainty and boundary limitations

Pilot-scale inventories are subject to uncertainty and may not reflect industrial-scale efficiencies; therefore, numerical results are interpreted as indicative hotspot patterns rather than definitive impact values. Omitted stages (e.g., transport, packaging, end-of-life) may become relevant at scale and will need inclusion in future LCA updates [18,19].

Residue status and allocation are recognised as key sources of uncertainty in valorisation LCAs [39,40]. In the pilot modelling, leek residues are treated as a processing side-stream whose upstream cultivation burdens are not assigned to the extract, consistent with cut-off conventions when the residue has low or negative economic value. However, as markets develop, residues may acquire value and could be considered co-products. Future work should therefore test alternative modelling choices including allocation (mass or economic) and system expansion, and conduct sensitivity analysis to support robust decision-making [39,40].

4. Results and discussion

4.1 LCA results

4.1.1 Environmental impacts of leek extract production (FU: 1 L of extract)

The production of 1 L of leek extract was associated with measurable environmental impacts across all the analysed categories, reflecting the experimental and pilot-scale nature of the process.

Impact values were particularly influenced by energy consumption and processing steps required for extraction and thermal treatment.

Table 1 presents the absolute environmental impacts associated with the production of leek extract, distinguishing between water use and extraction processes across multiple impact categories. The results indicate that the extraction phase contributes to all impact categories, while water use is reported only in terms of resource deprivation. Among the reported values, acidification (0.86 mol H^+ eq x 10^{-4}) and ozone depletion (0.82 kg CFC11 eq x10^{-9}) emerge as the most affected impact categories, suggesting that the extraction process carries a notable environmental burden in these areas. Human toxicity impacts are also relevant, with cancer-related toxicity at 0.15 CTUh x10^{-10} and non-cancer toxicity at 0.67 CTUh x10^{-9}, highlighting potential environmental and health considerations associated with the extraction phase. Eutrophication impacts vary across categories, with freshwater eutrophication (0.70 kg P eq x10^{-5}) presenting the highest burden, while marine (0.13 kg N eq x10^{-4}) and terrestrial eutrophication (0.13 mol N eq x10^{-3}) show relatively lower values. Similarly, particulate matter formation (0.79 disease inc. x10^{-9}) and photochemical ozone formation (0.49 kg NMVOC eq x10^{-4}) indicate moderate contributions from the extraction process. In terms of resource use, the extraction process shows an impact of 0.14 MJ for fossil resources and 0.77 kg Sb eq for mineral and metal resources, indicating a measurable resource demand. Notably, water use (0.449 m^3 depriv.) is exclusively attributed to water consumption rather than extraction, while climate change impact (0.11 kg CO_2 eq x10^{-1}) remains minimal, suggesting a relatively low carbon footprint in absolute terms. Overall, the results highlight that the extraction process drives all impact categories, while water use is limited to direct resource deprivation. Acidification, ozone depletion, and human toxicity are among the most significant impact categories, whereas climate change and fossil resource use appear to have a minor contribution.

Table 1. Absolute impacts for the functional unit (1 L of leek-extract)

Impact Categories	Unit of measure	Impact
Acidification	mol H+ eq x10^{-4}	0.86
Climate change	kg CO2 eq x 10^{-1}	0.11
Ecotoxicity, freshwater	CTUe	0.11
Particulate matter	disease inc. x10^{-9}	0.79
Eutrophication, marine	kg N eq x10^{-4}	0.13
Eutrophication, freshwater	kg P eq x10^{-5}	0.70
Eutrophication, terrestrial	mol N eq x10^{-3}	0.13
Human toxicity, cancer	CTUh x10^{-10}	0.15
Human toxicity, non-cancer	CTUh x10^{-9}	0.67
Ozone depletion	kg CFC11 eq x10^{-9}	0.82
Photochemical ozone formation	kg NMVOC eq x10^{-4}	0.49
Resource use, fossils	MJ	0.14
Resource use, minerals and metals	kg Sb eq x10^{-6}	0.77
Water use	m3 depriv.	0.500

4.1.2 Environmental impacts of lettuce cultivation (FU: 1 kg of lettuce)

When impacts were expressed per kilogram of lettuce, the alternative cultivation system showed lower values than the baseline in several impact categories (Table 2). This Table presents the environmental impact values for the two cultivation scenarios, Alternative and Baseline, across multiple impact categories. The results show variations in environmental burdens between the two approaches, with Alternative generally exhibiting lower impacts in most categories. Climate change impact shows a slight reduction, with Alternative (0.603 kg CO_2 eq x10^{-1}) performing slightly better than Baseline (0.609 kg CO_2 eq x10^{-1}). Similarly, acidification is lower in the alternative scenario (0.954 mol H^+ eq x10^{-2}) compared to baseline (1.089 mol H^+ eq x10^{-2}). For eutrophication, the results indicate a reduction in all subcategories under Alternative, with marine eutrophication decreasing from 0.372 kg N eq x10^{-2} to 0.325 kg N eq x10^{-2}, freshwater eutrophication from 0.456 kg P eq x10^{-4} to 0.428 kg P eq x10^{-4}, and terrestrial eutrophication from 0.482 mol N eq x10^{-1} to 0.421 mol N eq x10^{-1}. Toxicity-related impacts display mixed trends. Human toxicity, cancer is lower in the baseline scenario (0.202 CTUh x10^{-10}) compared to Alternative (0.259 CTUh x10^{-10}), whereas human toxicity, non-cancer is higher in the baseline scenario (0.496 CTUh x10^{-9}) compared to Alternative (0.729 CTUh x10^{-9}). Ecotoxicity in

freshwater follows a similar pattern, with Alternative (0.701 CTUe) showing lower impacts than Baseline (0.817 CTUe). Resource use indicators present marginal differences. Fossil resource use is slightly lower in Alternative (0.744 MJ) compared to Baseline (0.753 MJ), whereas mineral and metal resource use is higher in the alternative scenario (0.663 kg Sb eq x10^{-6} vs. 0.309 kg Sb eq x10^{-6} in the baseline). Finally, water consumption is nearly the same in both scenarios, with Alternative (2.951 m^3 depriv.) and Baseline (2.988 m^3 depriv.) showing minimal variation. The Alternative tends to show lower environmental burdens across most impact categories, with the exception of cancer-related toxicity and mineral resource use, where the baseline scenario performs better. This increase in human toxicity (cancer) and mineral resource use is primarily due to the additional energy-intensive steps and the specific laboratory materials required for the production of the leek extract, which are not required in the traditional baseline scenario. These findings highlight trade-offs between different impact categories, with neither scenario showing a clear overall dominance.

The results of LCA study, for both the considered functional units, cannot be compared with other literature data because no previous LCA studies were carried out on the production of leek extract and its application during lettuce cultivation. Despite this, the achieved results allow the identification of the main contributors to the environmental impact and represent a starting point for a further optimisation of the process.

Table 2. Absolute values related to the functional unit of 1 kg of fresh lettuce.

Impact Categories	Unit of measure	Baseline	Alternative	Δ %
Acidification	mol H+ eq x10^{-2}	1.089	0.954	−12.4%
Climate Change	kg CO2 eq x10^{-1}	0.609	0.603	−1.0%
Ecotoxicity, freshwater	CTUe	0.817	0.701	−14.2%
Particulate Matter	disease inc. x10^{-7}	0.749	0.658	−12.1%
Eutrophication, marine	kg N eq x10^{-2}	0.372	0.325	−12.6%
Eutrophication, freshwater	kg P eq x10^{-4}	0.456	0.428	−6.1%
Eutrophication, terrestrial	mol N eq x10^{-1}	0.482	0.421	−12.7%
Human Toxicity, cancer	CTUh x10^{-10}	0.202	0.259	+28.2%
Human Toxicity, non-cancer	CTUh x10^{-9}	0.496	0.729	+47.0%
Ozone Depletion	kg CFC11 eq x10^{-8}	0.179	0.146	−18.4%
Photochemical Ozone Formation	kg NMVOC eq x10^{-3}	0.291	0.284	−2.4%
Resource Use, fossil	MJ	0.753	0.744	−1.2%
Resource Use, mineral and metal	kg Sb eq x10^{-6}	0.309	0.663	+114.6%
Water Use	m3 depriv.	2.988	2.951	−1.2%

4.1.3 Relative contribution analysis (baseline vs alternative)

Fig 2 presents the relative contribution of different processes to the various impact categories in the LCA study. The results highlight a strong predominance of certain contributions across multiple categories, with some sources overwhelmingly driving specific environmental burdens. Acidification (97.5%), marine eutrophication (97.9%), terrestrial eutrophication (98.3%), and particulate matter formation (98.6%) are almost entirely attributed to emissions from nitrogen and phosphorus compounds (Em. N-P Compounds). Similarly, freshwater eutrophication shows a major contribution from this source (83.5%), although it is more distributed than other eutrophication-related categories. For climate change, the contribution is more distributed, with production factors (48.3%) and electricity (36.3%) being the dominant contributors, while emissions from N-P compounds play a smaller role (11.2%). This contrasts with categories such as human toxicity (cancer and non-cancer), where the contribution is more balanced across different sources. Human toxicity, cancer is primarily influenced by operational activities (35.9%), production factors (34.8%), and electricity use (28.9%), whereas human toxicity, non-cancer is mainly driven by production factors (54.0%), followed by electricity (21.7%) and operational

activities (12.4%). A similar trend is observed in ozone depletion (70.8% from production factors, 27.4% from electricity) and photochemical ozone formation (72.1% from production factors, 22.6% from electricity), indicating a more complex distribution of environmental burdens. Fossil resource use is split between production factors (50.4%) and electricity (45.6%), while mineral and metal resource use follows a similar pattern, with 71.4% from production factors and 12.6% from electricity. Lastly, freshwater ecotoxicity shows a more distributed profile, with production factors (46.4%), emissions from N-P compounds (32.5%), and electricity (10.7%) all playing significant roles. This is one of the few impact categories where emissions from N-P compounds, while still relevant, are not the primary driver. Overall, the results indicate that emissions from nitrogen and phosphorus compounds overwhelmingly drive acidification, particulate matter formation, and eutrophication, while production factors and electricity use dominate climate change, human toxicity, ozone depletion, and resource use categories. The contribution of operational activities remains relevant in specific categories, particularly in human toxicity and resource-related impacts, but its role is generally secondary compared to other factors.

You could have set this instead of all the previous paragraph. this is clearer and avoids confusion.

[**Fig 2.** Baseline scenario related to the functional unit of 1 kg of fresh lettuce.]

In addition, Fig 3 shows the relative contribution of different processes to the environmental impact categories in the LCA study. The results highlight distinct variations in contribution patterns across impact categories, with emissions from nitrogen and phosphorus compounds (Em. N-P compounds), production factors, and electricity being the dominant contributors in most cases, while the leek extract plays a relevant role in selected categories. ~~Acidification (97%), marine eutrophication (98%), terrestrial eutrophication (98%), and particulate matter formation (98%) are overwhelmingly driven by emissions from nitrogen and phosphorus compounds, indicating a strong concentration of environmental burdens in these categories. Freshwater eutrophication, while still largely influenced by N-P emissions (78%), shows a more distributed profile, with electricity~~

(7.1%) and the leek extract (12.4%) contributing to the overall impact. In contrast, climate change impacts are more evenly distributed among sources, with production factors (40.9%) and electricity (32.0%) being the primary contributors, while the leek extract (13.6%) and N-P emissions (10%) also have a notable influence. Similarly, human toxicity (cancer and non-cancer) presents a more complex distribution. Human toxicity, cancer is mainly influenced by the leek extract (44.0%), followed by operational activities (24.5%), electricity (19.7%), and production factors (11.9%). Non-cancer human toxicity exhibits a stronger influence from the leek extract (69.2%), while electricity (12.8%) and production factors (4.95%) contribute to a lesser extent. Ozone depletion (42.5%) and photochemical ozone formation (13.0%) show a notable contribution from the leek extract, while production factors (26.2% and 62.1%, respectively) and electricity (29.4% and 20.2%, respectively) also play significant roles. The use of fossil resources is mainly driven by production factors (42.5%) and electricity (40.3%), while the leek extract (13.7%) contributes a smaller share. Mineral and metal resource use, however, is overwhelmingly dominated by the leek extract (87.8%), making it the most significant factor in this category. Lastly, freshwater ecotoxicity presents a diverse contribution pattern, with production factors (21.9%), N-P emissions (59%), electricity (5.3%), and the leek extract (12.2%) all contributing in varying proportions. Overall, the results indicate that emissions from nitrogen and phosphorus compounds overwhelmingly drive acidification, particulate matter formation, and eutrophication, while production factors and electricity dominate climate change, human toxicity, ozone depletion, and resource use categories. The leek extract plays a significant role in human toxicity (cancer and non-cancer), ozone depletion, and resource use (minerals and metals), highlighting its influence in specific impact categories. These findings suggest that while certain interventions may reduce some environmental burdens, they may also introduce trade-offs in other categories, emphasizing the need for a balanced approach in impact mitigation strategies.

This is Redundant.

Just keep this, and add citations

[**Fig 3.** Alternative scenario related to the functional unit of 1 kg of fresh lettuce.]

In this context, it should be noted that the environmental comparison implicitly assumes a substitution logic. The application of the leek extract is intended to improve nutrient use efficiency, theoretically allowing for a reduction in conventional inputs. The net environmental performance of the alternative scenario is therefore contingent on this substitution effect; without a corresponding gain in system efficiency or a reduction in synthetic fertilizers, the biostimulant would represent an additional environmental load rather than a sustainable transition.

4.2 Sustainable business model results

The integrated SBMC-EBME representation is shown in Fig 3, while Table 3 details the business model configuration and the LCA-informed design levers that operationalise hotspot mitigation. While Table 3 specifies how hotspot mitigation is embedded in the SBMC through revenue streams and dedicated investments, the economic feasibility of this logic benefits from an explicit (albeit indicative) quantitative linkage between environmental priorities and incentives. At the current pilot stage, a full techno-economic assessment would be premature; nevertheless, the mitigation strategy can be framed in operational terms by expressing its main enabling costs as a per-batch and per-litre “verification-and-mitigation” component that is directly tied to the hotspots highlighted by the EF 3.1 results. Concretely, the incremental cost of hotspot mitigation is expected to be dominated by *(i)* batch-level QA/QC and contaminant screening that reduce toxicity-related risks and support regulatory legitimacy, *(ii)* metering/traceability and third-party checks that make sustainability claims financeable and credible, and *(iii)* the energy-related cost differential associated with efficiency improvements and low-carbon electricity procurement. These costs can be allocated transparently by dividing batch-level monitoring and testing expenditures by the litres produced per batch, and by adding an energy term calculated from measured kWh per litre multiplied by the applicable electricity price (or price differential for renewable procurement).

This simple accounting makes the incentive link explicit: the “verified sustainability” tier proposed in Table 3 becomes economically meaningful only if the resulting per-litre surcharge remains small relative to the customer-side value of risk reduction (compliance and safety), agronomic performance, and the willingness to pay for verification-backed claims. In this sense, the business model is designed to convert hotspot mitigation from an unfunded aspiration into a priced capability: the same variables that drive the LCA hotspots (e.g., kWh L^{-1}, recovery rates, QA/QC pass rates, contaminant thresholds, minimum effective dose) also define the cost base and the conditions under which a premium tier, service bundle, or performance-linked contract can be offered. By making these relationships explicit, the analysis strengthens the plausibility of the proposed mitigation logic without overstating economic precision at the pilot stage.

The sustainability aims of the project can be summarised as: (A1) valorise under-utilised leek residues and reduce disposal; (A2) create measurable environmental improvements in downstream horticultural production through a bio-based input; (A3) deliver a financially viable model that sustains extraction, compliance and continuous improvement; (A4) support local circular bioeconomy development via skills, jobs and cooperative governance; and (A5) create a replicable pathway for other Alliaceae chains. These aims align with SBM archetypes such as creating value from waste and substituting with renewables and natural processes [29,60].

The valorisation pathway depends on coordinated roles across the ecosystem. Upstream, farmers and the producer organisation ensure crop and residue supply and coordinate agronomic practices. The fresh-cut processor provides segregation and collection of residues and benefits from reduced waste management burdens. Downstream, the extraction operator and technology provider execute extraction and process optimisation. Analytical laboratories provide bioactive profiling and safety testing, essential for regulatory compliance and market legitimacy [12]. Agronomic partners validate efficacy and provide application protocols, while distributors and advisory networks support market access and adoption. Dependencies include feedstock quality (freshness, contamination), timing and storage, energy and water provisioning, solvent handling (where

applicable), traceability and conformity assessment. Such dependencies are consistent with circular innovation ecosystem frameworks emphasising complementarities, shared infrastructure and coordination costs [35,38,54].

The primary synergy is the conversion of a locally concentrated residue stream into a bio-based input that can reduce several environmental indicators in a downstream crop system. The FU2 LCA results provide multi-indicator evidence supporting this synergy, particularly for eutrophication and acidification indicators that are salient in agriculture [3,21]. Nevertheless, the toxicity and mineral/metal trade-offs represent a salient risk of burden shifting. From a systems perspective, such trade-offs can reflect extraction energy mix, auxiliary materials and equipment choices-categories often not visible in climate-only discussions [9]. They also have governance implications: stakeholders must ensure safe handling, avoid contaminants, and adopt procurement and process design that mitigates critical resource dependencies [37,43]. Additional risks include pilot-to-scale uncertainty (industrial efficiencies could change hotspots), regulatory barriers and compliance costs, and adoption uncertainty among farmers. These risks reinforce the value of an experimentation-oriented approach, where environmental and business model hypotheses are tested iteratively [30,55].

This part is crucial but was not discussed much. Where was this emphasized?

Hotspot translation yields a set of redesign levers prioritised for experimentation and governance. For extraction, priorities include: (i) energy profiling and process intensification (reducing kWh per litre), (ii) renewable electricity procurement, (iii) solvent and/or water loop design to reduce toxicity and water deprivation, and (iv) procurement and equipment strategies to reduce mineral/metal impacts. For the downstream crop system, a key lever is determining the minimum effective dose that achieves agronomic benefit with minimal upstream processing per unit of function [16,24]. Experimentation priorities are therefore: (E1) process optimisation trials; (E2) solvent selection and recovery design (aligned with safer-chemistry principles); (E3) water management and reuse options; (E4) quality assurance and standardisation protocols; and (E5) agronomic field trials and dose-response modelling. These levers are incorporated into SBMC

This sounds repetitive with the description of leek extract before

blocks to specify which actors implement them and how investments are financed through value capture [27,28]. → Note that lettuce was not ever mentioned.

Table 3 presents the SBMC for SOMMELIER as an ecosystem-level business model hypothesis. The table explicitly links LCA hotspot signals to business model design levers, addressing a common critique of SBM work-namely, moving from aspirational value propositions to operational and financeable interventions [26,27]. The SBMC emphasises that environmental performance is a joint outcome of operational decisions, governance and incentive alignment across actors [30]. In particular, the LCA-identified trade-offs motivate a hotspot-mitigation-by-design approach: the business model includes commitments to renewable electricity sourcing, closed-loop water/solvent management, safer chemical handling and materials-aware procurement. The value capture logic is structured to fund these commitments through hybrid revenue mechanisms (product + services, monitoring, verification-backed premium claims), consistent with sustainability business model theory [28,49].

Table 3. Sustainable Business Model Canvas applied to SOMMELIER: case configuration and LCA-informed environmental design levers.

SBMC building block	SOMMELIER configuration (what the BM is)	LCA-informed environmental levers (readable, measurable)
Key stakeholders / partners	Producer organisation + farmers; fresh-cut processor; extraction operator; technology provider; analytical labs; agronomic trial partners; conformity bodies; horticultural farms; logistics; public innovation programme.	Partner selection with minimum sustainability conditions: *(i)* extraction site powered mainly by renewables (high renewable share); *(ii)* residue supply agreements specifying quality and timing to avoid extra processing; *(iii)* annual third-party verification of traceability and environmental claims.
Key activities	Residue segregation; storage/stabilisation; extraction (water/ethanol); filtration/standardisation; QA/QC; agronomic formulation & guidance; compliance; marketing & support.	Focus on the LCA hotspots of extraction: *(i)* reduce energy use per litre of extract vs the pilot baseline; *(ii)* adopt water/solvent recirculation where feasible; *(iii)* introduce a safer-chemistry check for auxiliaries to address the toxicity trade-off; *(iv)* update LCA after major process changes.
Key resources & capabilities	Stable residue stream; extraction equipment; lab for bioactives/contaminants; agronomic expertise; regulatory capability; traceability/data; logistics.	Capability built around evidence: *(i)* batch-level metering for energy and water; *(ii)* traceability to support multi-indicator environmental communication; *(iii)* equipment strategy prioritising long lifetime and shared use (hub model) to reduce resource-related burdens over time.
Customer segments	Leafy vegetable farms; organic/integrated; distributors for bio-based inputs; cooperatives seeking circular inputs.	Target customers valuing verified impacts: offer a "verified sustainability" tier only when LCA shows net improvements across multiple indicators and no significant increase in toxicity/resource use; maintain

		a conservative claim strategy to prevent burden shifting.
Channels	Direct B2B via cooperative; demo farms; agronomic advisory; distributor partnerships; innovation showcases.	Evidence-based go-to-market: provide customers with a short environmental factsheet reporting a small set of indicators (e.g., climate change, eutrophication/acidification, toxicity/resource-use flags) and the date of the latest LCA update.
Customer relationships	Technical support & co-learning; performance monitoring; long-term supply; feedback loops; training for safe handling.	Monitoring to protect environmental performance: *(i)* guidance to keep application within recommended dose ("minimum effective dose" principle); *(ii)* seasonal feedback from farms to update performance assumptions; *(iii)* safe handling training to address concerns linked to toxicity categories.
Value proposition – Planet	Valorise leek residues; reduce disposal; improve lettuce performance across multiple EF categories; reduce reliance on conventional inputs where feasible.	Two-part environmental promise: *(i)* preserve improvements in key categories (e.g., eutrophication/acidification/ecotoxicity) shown by the LCA; *(ii)* explicitly manage trade-offs by committing to reduce the toxicity and mineral/metal resource signals through process redesign (energy source, solvent choice, recovery loops), with annual verification.
Value proposition – People	Jobs/skills; cooperative governance; farmer income diversification; compliance/transparency under EU fertilising products regulation.	Measurable co-benefits: training delivered annually; transparent reporting of compliance steps; stakeholder inclusion and fair value sharing, supported by traceability and third-party checks.
Value proposition – Profit	New revenue stream; lower waste handling cost; differentiated biostimulant; sustainability premium; leverage public funding for early investment.	Profit aligned with mitigation: pricing includes a small "verification and mitigation" component covering monitoring, recovery and testing; premiums are conditional on maintaining verified multi-indicator improvements (avoid greenwashing risk).
Cost structure	Capex extraction/recovery; Opex energy/water/solvents/labour; testing/compliance; logistics; advisory.	Cost priorities guided by hotspots: invest first in options that reduce the main burdens (energy, water/solvent use, toxicity-related auxiliaries); treat monitoring and recovery as enabling costs required for verified claims. To make feasibility transparent, key cost items are tracked in allocable units (e.g., € per batch for QA/QC and third-party verification; kWh per litre and €/kWh for energy; CAPEX amortisation per litre in hub configurations).
Revenue streams / value capture	Product sales; advisory & monitoring services; licensing/replication; shared savings with processor & farms.	Value capture linked to evidence: additional revenues (premium/service fees) depend on verified environmental performance; shared-savings contracts align residue quality and process efficiency incentives across the chain. Operationally, the verification-and-mitigation component can be implemented as a per-litre surcharge equal to (QA/QC + monitoring + verification cost per batch)/(litres per batch) plus an energy term based on measured kWh L^{-1}, and applied only to the 'verified' tier.

4.3 Implementation and scaling logic

Implementing the proposed sustainable business model requires explicit decisions on (i) the location and scale of extraction capacity, (ii) residue stabilisation and quality management, and (iii) the governance arrangements that coordinate processors, producers and downstream customers. These choices affect not only economic feasibility but also the system-wide environmental profile, because the LCA results indicate that benefits at crop level coexist with non-negligible trade-offs driven by the extract supply chain and its auxiliary inputs. To test the sensitivity of the proposed

business model and its environmental hotspots to different logistical and scale configurations, two stylised implementation scenarios are therefore discussed as alternative pathways for operationalising the SBMC hypothesis (Table 3) and for interpreting the ecosystem-level design choices represented in Fig 4.

Scenario S1 (decentralised, near-source extraction): This scenario places small modular units close to the fresh-cut processor, thereby reducing residue transport distances and allowing rapid processing of a time-sensitive biomass stream. This configuration can lower logistics burdens and mitigate quality deterioration, potentially reducing the need for stabilisation steps. However, decentralised systems may show higher unit costs and may struggle to achieve high recovery efficiency and robust monitoring at small scale, particularly when advanced solvent/water recirculation or energy-efficiency upgrades require capital-intensive equipment [35]. From a hotspot-mitigation perspective, S1 performs best when paired with operational controls that prevent burden shifting, including stringent residue segregation protocols, minimum effective dose strategies, and performance monitoring that links application guidance to field conditions.

Scenario S2 (regional extraction hub): This scenario aggregates residues from multiple processors into a shared facility, enabling economies of scale in extraction, quality control and utilities management. A hub can justify investments in best-available recovery systems (e.g., closed-loop water/solvent management) and in contracted low-carbon electricity supply, and it can institutionalise third-party verification and traceability as part of routine operations [38,54]. These features align directly with the LCA-informed design levers in Table 3 and can be particularly relevant for managing toxicity- and mineral/metal-related trade-offs through standardised procurement, safer-chemistry screening and longer-lived equipment strategies. The main drawbacks are coordination complexity and the need for governance mechanisms that define residue quality standards, scheduling rules, and transparent benefit sharing among participating actors [30].

Across both scenarios, the SBMC emphasises that environmental performance is a joint outcome of process design and ecosystem governance. Accordingly, Table 3 and Fig 4 should be interpreted as a modular blueprint: the business model building blocks remain stable, while the configuration of stakeholders, cost structure and value capture mechanisms can be adapted to the chosen scaling pathway. In practical terms, S1 prioritises proximity and flexibility, whereas S2 prioritises infrastructure efficiency and measurable hotspot mitigation. These scenario logics provide a decision frame for implementation and for future empirical testing of the business model hypothesis under real-world operating conditions

[**Fig 4.** SBMC-EBME canvas for SOMMELIER]

5. Discussion and managerial implications

Combined Result with the discussion section. Decrease redundancy and helps to understand the implications

SOMMELIER illustrates an empirically common pattern in circular bioeconomy innovations: the potential for improvements in multiple impact categories downstream coupled with trade-offs driven by upstream upgrading [5,8]. The alternative lettuce scenario indicates reductions in eutrophication and acidification-related indicators-highly salient in agriculture-yet significant increases in toxicity and mineral/metal resource use. These results caution against ‘circular-equals-sustainable’ narratives and reinforce calls for multi-indicator evaluation to avoid burden shifting [9,42]. Transferability beyond the focal case depends primarily on the availability of two key conditions. First, it requires access to a process-based inventory describing the residue upgrading step, since the environmental assessment relies on detailed information about material and energy flows associated with the transformation of waste into a usable input. Second, it requires a clear definition of the downstream function through which environmental and economic benefits are generated, such as reductions in fertiliser or pesticide use, or improvements in yield stability. When these elements are specified, the analytical approach developed in this study can be applied in other contexts following a structured but adaptable logic. In practice, the process involves conducting an

LCA using a harmonised methodology (here the EF 3.1 framework) to identify the environmental hotspots associated with the production pathway. These quantified hotspot drivers can then be systematically translated into specific business model design choices within the Sustainable Business Model Canvas, linking environmental performance to operational decisions, actor responsibilities, and governance arrangements.

Importantly, the approach also emphasises the need to progressively integrate operational data as the pathway moves from experimental to scaled implementation. For this reason, the methodological section clarifies which variables should be systematically monitored in future applications, including energy requirements per unit of output, material recovery rates, the minimum effective agronomic dose, and quality assurance thresholds. Tracking these parameters over time allows both the LCA results and the associated business model assumptions to be updated as empirical evidence accumulates. In this way, the proposed LCA-business model integration is not limited to the specific case analysed here, but offers a replicable framework that can support the design and evaluation of similar residue-to-input valorisation pathways in other agri-food contexts.

For biostimulants specifically, toxicity hotspots are governance-relevant because legitimacy and compliance depend on safety and traceability [12,13]. As such, the business model must not only aim to valorise residues but also embed protocols, monitoring and third-party verification that address these hotspots [47]. For example, this may include batch-level contaminant screening performed by accredited laboratories and digital traceability systems combined with third-party certification of environmental performance claims.

From a theoretical perspective, this study contributes to the emerging interface between environmental assessment and SBM research by demonstrating how quantified LCA outputs can be operationalised within an ecosystem-oriented business model design process. Rather than treating environmental assessment as a separate analytical exercise, the proposed approach embeds LCA hotspot information directly into business model configuration and experimentation. The

SBMC structure ensures that hotspots are mapped to specific activities, resources and cost drivers, and that responsibilities and financing mechanisms are made explicit [30,56].

The EBME lens highlights that the focal SBMC depends on complementary business models-residue segregation at the processor, extraction services, laboratory testing, agronomic advisory networks and farm adoption [38]. In such ecologies, sustainability outcomes emerge from the alignment and co-evolution of multiple models rather than from a single firm-level optimisation. LCA-informed design levers can act as ‘boundary objects’ that different actors can mobilise during experimentation and governance [55,61].

Compared with the TLBMC, which separates economic, environmental and social layers, the SBMC integrates these dimensions and keeps value capture central [56]. This integration matters when environmental improvements require investment: specifying how value is captured to finance hotspot mitigation reduces the risk that sustainability remains aspirational or underfunded [27].

Three managerial implications follow. First, extraction-stage optimisation should be prioritised. Energy efficiency and renewable electricity procurement can influence multiple impact categories and stabilise operating costs, consistent with SBM archetypes focusing on efficiency and renewable [29].

Second, toxicity and critical resource hotspots require proactive management through safer chemistry screening, closed-loop handling, supplier requirements and monitoring. These actions should be embedded as capabilities (resources) rather than treated as compliance overhead, because they enable legitimacy and premium positioning [49].

Third, value capture mechanisms should be designed to fund mitigation. From an implementation standpoint, this implies reporting at least a small set of economic parameters alongside hotspot metrics - such as testing/verification cost per batch, litres per batch, and energy cost per litre - so that the feasibility of mitigation can be assessed as the pathway scales and business model assumptions are updated with operational data.

Hybrid models combining product sales with services (advisory, monitoring) and performance-based contracts can align incentives and finance continuous improvement [27,28]. For cooperatives, residue contracts should specify quality and timing and include incentives for segregation and freshness to reduce processing intensity and losses [54].

Scaling decisions (decentralised vs hub) should consider not only cost but also the capacity to mitigate hotspots. Shared hubs may justify investments in recovery infrastructure, whereas decentralised units may reduce transport and spoilage. These choices reflect the broader circular supply chain challenge of aligning technical efficiency with governance complexity [35,54].

Innovation programmes and policymakers can increase the credibility of circular bioeconomy support by requiring LCA-informed business model experimentation deliverables [24]. Funding criteria could include hotspot mitigation plans, verification strategies and periodic LCA updates using operational data, rather than prototype demonstrations alone [25].

Public support can also target enabling infrastructures (renewable energy access, water services, shared extraction hubs, accredited testing laboratories) that lower the cost of hotspot mitigation for SMEs and cooperatives [7,35]. In parallel, regulatory frameworks such as Regulation (EU) 2019/1009 can reduce market uncertainty but impose compliance costs; targeted support for conformity assessment may accelerate adoption while safeguarding safety and transparency [12].

6. Conclusions

This paper developed and demonstrated an LCA-informed sustainable business model innovation procedure for circular bioeconomy pathways and illustrated it through the SOMMELIER project valorising leek processing residues into bioactive biostimulant extracts. The comparative LCA indicates that the alternative lettuce scenario reduces most EF midpoint indicators, particularly eutrophication- and acidification-related impacts, suggesting environmental value creation potential. At the same time, significant trade-offs emerge for toxicity and mineral/metal resource use, emphasising the risk of burden shifting and the need for targeted mitigation.

By integrating EBME with SBMC, we translated quantified hotspots into explicit business model design levers and clarified how value capture mechanisms can finance mitigation. Practically, the approach supports cooperatives and processors in prioritising extraction optimisation, safer chemistry and recovery loops, and in designing contracts and pricing schemes that support verified sustainability.

For policymakers and innovation programmes, the case illustrates the value of requiring LCA-informed business model experimentation deliverables to increase the probability that pilots lead to scalable and credible circular solutions.

The study has some limitations. First, the LCA is based on pilot-scale modelling with limited inventory detail and truncated system boundaries for the extract; transport, packaging and end-of-life may become non-negligible at scale.

Second, key LCA methodological choices-residue status, allocation, substitution assumptions-can change results. Future work should test alternative allocation and system expansion rules and quantify uncertainty.

Third, agronomic efficacy evidence is embedded in the scenario comparison but not presented as a full agronomic statistical study; scaling will require multi-season, multi-site trials to confirm dose-response relationships and stability across conditions.

Fourth, this paper focuses primarily on environmental aspects; social impacts are discussed conceptually but could be strengthened through complementary assessments and stakeholder research.

Future research should extend the approach along four lines. (1) Scaling scenarios: model industrial-scale extraction with alternative energy mixes and recovery efficiencies to test whether pilot hotspots persist or diminish. (2) Consequential modelling: explore substitution effects and market-mediated impacts, including potential rebounds. (3) Integrated techno-economic and BM-level assessment: combine LCA with cost modelling and business model LCA approaches to compare configurations per unit of economic value. (4) Governance and adoption research: analyse

how cooperatives allocate benefits, how verification influences willingness to pay and adoption, and how policy instruments affect business model choices.

Methodologically, developing standard ‘hotspot-to-canvas’ mapping protocols and testing them across residue-to-input pathways could generate more generalisable design principles for LCA-informed sustainable business model experimentation.

Beyond the specific SOMMELIER case, the study also provides practical insights for managers and cooperative organisations engaged in circular bioeconomy initiatives. The findings suggest that environmental assessment can play a strategic role in guiding business model design rather than being used only as an ex-post evaluation tool. In practice, this implies that actors involved in residue valorisation pathways should integrate environmental hotspot analysis early in the innovation process and use it to prioritise operational improvements, supplier requirements and investment decisions. For instance, extraction-stage optimisation, renewable energy sourcing, and transparent monitoring systems emerge as managerial priorities that can simultaneously reduce environmental burdens and strengthen market credibility.

More broadly, the proposed integration between LCA evidence and sustainable business model design offers a structured decision-support approach for organisations seeking to scale residue-based innovations while avoiding unintended environmental trade-offs. By explicitly linking environmental performance indicators to governance mechanisms and value capture strategies, the framework can help managers design circular business models that remain environmentally credible as they move from pilot experimentation to commercial implementation.

Author Contributions

Conceptualization: Zilia, F.; Orsi, L.

Methodology: Zilia, F., Ferraro, L., Bacenetti, J.

Supervision: Orsi, L., Bacenetti, J.

Writing – original draft: Zilia, F., Ferraro, L.

Writing – review & editing: All authors

Data Availability Statement

The data supporting the findings of this study are available from the corresponding author upon reasonable request.

Conflicts of Interest

Federico Zilia serves as an Academic Editor for PLOS ONE. To avoid any potential conflict of interest, we request that he is not involved in the handling of this submission. This does not alter our adherence to PLOS ONE policies on sharing data and materials.

References

[1] FAO. Global food losses and food waste – Extent, causes and prevention. FAO; 2011.

[2] Foley JA, Ramankutty N, Brauman KA, Cassidy ES, Gerber JS, Johnston M, et al. Solutions for a cultivated planet. Nature 2011;478:337–42. https://doi.org/10.1038/nature10452.

[3] Notarnicola B, Tassielli G, Renzulli PA, Castellani V, Sala S. Environmental impacts of food consumption in Europe. Eco-Effic Agric Food Syst Sel Pap Addressing Glob Chall Food Syst Those Present Conf "LCA Feed Planet Energy Life" 6-8 Oct 2015 Stresa Milan Expo Italy 2017;140:753–65. https://doi.org/10.1016/j.jclepro.2016.06.080.

[4] Girotto F, Alibardi L, Cossu R. Food waste generation and industrial uses: A review. Urban Min 2015;45:32–41. https://doi.org/10.1016/j.wasman.2015.06.008.

[5] Mirabella N, Castellani V, Sala S. Current options for the valorization of food manufacturing waste: a review. J Clean Prod 2014;65:28–41. https://doi.org/10.1016/j.jclepro.2013.10.051.

[6] Haukkala T. The EU Circular Economy Action Plan: Key Assumptions and Strategic Risks. LIEPP Policy Brief 2025;79.

[7] Stephenson PJ, Damerell A. Bioeconomy and Circular Economy Approaches Need to Enhance the Focus on Biodiversity to Achieve Sustainability. Sustainability 2022;14:10643. https://doi.org/10.3390/su141710643.

[8] Zink T, Geyer R. Circular Economy Rebound. J Ind Ecol 2017;21:593–602. https://doi.org/10.1111/jiec.12545.

[9] Kirchherr J, van Santen R. Research on the circular economy: A critique of the field. Resour Conserv Recycl 2019;151:104480. https://doi.org/10.1016/j.resconrec.2019.104480.

[10] Kirchherr J, Reike D, Hekkert M. Conceptualizing the circular economy: An analysis of 114 definitions. Resour Conserv Recycl 2017;127:221–32. https://doi.org/10.1016/j.resconrec.2017.09.005.

[11] Blomsma F, Brennan G. The Emergence of Circular Economy: A New Framing Around Prolonging Resource Productivity. J Ind Ecol 2017;21:603–14. https://doi.org/10.1111/jiec.12603.

[12] European Union. Regulation (EU) 2019/1009 of the European Parliament and of the Council of 5 June 2019 laying down rules on the making available on the market of EU fertilising products … and repealing Regulation (EC) No 2003/2003 2019.

[13] du Jardin P. Plant biostimulants: Definition, concept, main categories and regulation. Biostimulants Hortic 2015;196:3–14. https://doi.org/10.1016/j.scienta.2015.09.021.

[14] Calvo P, Nelson L, Kloepper JW. Agricultural uses of plant biostimulants. Plant Soil 2014;383:3–41. https://doi.org/10.1007/s11104-014-2131-8.

[15] Yakhin OI, Lubyanov AA, Yakhin IA, Brown PH. Biostimulants in Plant Science: A Global Perspective. Front Plant Sci 2017;Volume 7-2016. https://doi.org/10.3389/fpls.2016.02049.

[16] Rouphael Y, Colla G. Editorial: Biostimulants in Agriculture. Front Plant Sci 2020;Volume 11-2020. https://doi.org/10.3389/fpls.2020.00040.

[17] Bulgari R, Franzoni G, Ferrante A. Biostimulants Application in Horticultural Crops under Abiotic Stress Conditions. Agronomy 2019;9:306. https://doi.org/10.3390/agronomy9060306.

[18] ISO T. 207 (2006) ISO 14040: 2006 environmental management—life cycle assessment—principles and framework. ISO Switz 2006.

[19] ISO E. 14044 2018 Environmental management–Life cycle assessment–Requirements and guidelines (ISO 14044: 2006+ Amd 1: 2017). Ger Version EN ISO 14044 2006 A1 2018.

[20] Rebitzer G, Ekvall T, Frischknecht R, Hunkeler D, Norris G, Rydberg T, et al. Life cycle assessment: Part 1: Framework, goal and scope definition, inventory analysis, and applications. Environ Int 2004;30:701–20. https://doi.org/10.1016/j.envint.2003.11.005.

[21] van der Werf HMG, Knudsen MT, Cederberg C. Towards better representation of organic agriculture in life cycle assessment. Nat Sustain 2020;3:419–25. https://doi.org/10.1038/s41893-020-0489-6.

[22] de Backer E, Aertsens J, Vergucht S, Steurbaut W. Assessing the ecological soundness of organic and conventional agriculture by means of life cycle assessment (LCA): A case study of leek production. Br Food J 2009;111:1028–61. https://doi.org/10.1108/00070700910992916.

[23] Zilia F, Andreottola FG, Orsi L, Parolini M, Bacenetti J. Trash or treasure? A circular business model of recycling plasmix. Circ Econ 2024;3:100089. https://doi.org/10.1016/j.cec.2024.100089.

[24] Geissdoerfer M, Pieroni MPP, Pigosso DCA, Soufani K. Circular business models: A review. J Clean Prod 2020;277:123741. https://doi.org/10.1016/j.jclepro.2020.123741.

[25] Böckin D, Goffetti G, Baumann H, Tillman A-M, Zobel T. Business model life cycle assessment: A method for analysing the environmental performance of business. Sustain Prod Consum 2022;32:112–24. https://doi.org/10.1016/j.spc.2022.04.014.

[26] Lüdeke-Freund F, Dembek K. Sustainable business model research and practice: Emerging field or passing fancy? J Clean Prod 2017;168:1668–78. https://doi.org/10.1016/j.jclepro.2017.08.093.

[27] Evans S, Vladimirova D, Holgado M, Van Fossen K, Yang M, Silva EA, et al. Business Model Innovation for Sustainability: Towards a Unified Perspective for Creation of Sustainable Business Models. Bus Strategy Environ 2017;26:597–608. https://doi.org/10.1002/bse.1939.

[28] Schaltegger S, Lüdeke-Freund F, Hansen EG. Business Models for Sustainability: A Co-Evolutionary Analysis of Sustainable Entrepreneurship, Innovation, and Transformation. Organ Environ 2016;29:264–89. https://doi.org/10.1177/1086026616633272.

[29] Bocken NMP, Short SW, Rana P, Evans S. A literature and practice review to develop sustainable business model archetypes. J Clean Prod 2014;65:42–56. https://doi.org/10.1016/j.jclepro.2013.11.039.

[30] Bocken N, Boons F, Baldassarre B. Sustainable business model experimentation by understanding ecologies of business models. J Clean Prod 2019;208:1498–512. https://doi.org/10.1016/j.jclepro.2018.10.159.

[31] Bocken NMP, Geradts THJ. Barriers and drivers to sustainable business model innovation: Organization design and dynamic capabilities. Long Range Plann 2020;53:101950. https://doi.org/10.1016/j.lrp.2019.101950.

[32] Ghisellini P, Cialani C, Ulgiati S. A review on circular economy: the expected transition to a balanced interplay of environmental and economic systems. Post Foss Carbon Soc Regen Prev Eco-Ind Dev 2016;114:11–32. https://doi.org/10.1016/j.jclepro.2015.09.007.

[33] EMF. Towards the Circular Economy: Economic and business rationale for an accelerated transition. 2013.

[34] Schroeder P, Anggraeni K, Weber U. The Relevance of Circular Economy Practices to the Sustainable Development Goals. J Ind Ecol 2019;23:77–95. https://doi.org/10.1111/jiec.12732.

[35] Rizos V, Behrens A, Van der Gaast W, Hofman E, Ioannou A, Kafyeke T, et al. Implementation of Circular Economy Business Models by Small and Medium-Sized Enterprises (SMEs): Barriers and Enablers. Sustainability 2016;8:1212. https://doi.org/10.3390/su8111212.

[36] Antikainen M, Valkokari K. A Framework for Sustainable Circular Business Model Innovation. Technol Innov Manag Rev 2016;6:5–12. https://doi.org/10.22215/timreview/1000.

[37] Manninen K, Koskela S, Antikainen R, Bocken N, Dahlbo H, Aminoff A. Do circular economy business models capture intended environmental value propositions? J Clean Prod 2018;171:413–22. https://doi.org/10.1016/j.jclepro.2017.10.003.

[38] Konietzko J, Bocken N, Hultink EJ. A Tool to Analyze, Ideate and Develop Circular Innovation Ecosystems. Sustainability 2020;12:417. https://doi.org/10.3390/su12010417.

[39] Weidema B. Avoiding Co-Product Allocation in Life-Cycle Assessment. J Ind Ecol 2000;4:11–33. https://doi.org/10.1162/108819800300106366.

[40] Ekvall T, Weidema BP. System boundaries and input data in consequential life cycle inventory analysis. Int J Life Cycle Assess 2004;9:161–71. https://doi.org/10.1007/BF02994190.

[41] Guinee J. Handbook on Life Cycle Assessment. An Operational Guide to the ISO Standards. Int J Life Cycle Assess 2001;7:311–3. https://doi.org/10.1007/BF02978897.

[42] Zampori L, Pant R. Suggestions for updating the Product Environmental Footprint (PEF) method 2019. https://doi.org/10.2760/424613%2520(online),10.2760/265244%2520(print).

[43] Rosenbaum RK, Bachmann TM, Gold LS, Huijbregts MAJ, Jolliet O, Juraske R, et al. USEtox—the UNEP-SETAC toxicity model: recommended characterisation factors for human toxicity and freshwater ecotoxicity in life cycle impact assessment. Int J Life Cycle Assess 2008;13:532–46. https://doi.org/10.1007/s11367-008-0038-4.

[44] Stubbs W, Cocklin C. Conceptualizing a "Sustainability Business Model." Organ Environ 2008;21:103–27. https://doi.org/10.1177/1086026608318042.

[45] Boons F, Lüdeke-Freund F. Business models for sustainable innovation: state-of-the-art and steps towards a research agenda. Sustain Innov Bus Models 2013;45:9–19. https://doi.org/10.1016/j.jclepro.2012.07.007.

[46] Teece DJ. Business Models, Business Strategy and Innovation. Bus Models 2010;43:172–94. https://doi.org/10.1016/j.lrp.2009.07.003.

[47] Zott C, Amit R, Massa L. The Business Model: Recent Developments and Future Research. J Manag 2011;37:1019–42. https://doi.org/10.1177/0149206311406265.

[48] Upward A, Jones P. An Ontology for Strongly Sustainable Business Models: Defining an Enterprise Framework Compatible With Natural and Social Science. Organ Environ 2016;29:97–123. https://doi.org/10.1177/1086026615592933.

[49] Freudenreich B, Lüdeke-Freund F, Schaltegger S. A Stakeholder Theory Perspective on Business Models: Value Creation for Sustainability. J Bus Ethics 2020;166:3–18. https://doi.org/10.1007/s10551-019-04112-z.

[50] Osterwalder A, Pigneur Y. Business Model Generation: A Handbook for Visionaries, Game Changers, and Challengers. 2010.

[51] Joyce A, Paquin RL. The triple layered business model canvas: A tool to design more sustainable business models. J Clean Prod 2016;135:1474–86. https://doi.org/10.1016/j.jclepro.2016.06.067.

[52] Lozano R. Sustainable business models: Providing a more holistic perspective. Bus Strategy Environ 2018;27:1159–66. https://doi.org/10.1002/bse.2059.

[53] Zilia F, Orsi L, Costantini M, Tedesco DEA, Sugni M. Case study of Life Cycle Assessment and sustainable business model for sea urchin waste. Clean Environ Syst 2023;8:100108. https://doi.org/10.1016/j.cesys.2023.100108.

[54] Geissdoerfer M, Morioka SN, de Carvalho MM, Evans S. Business models and supply chains for the circular economy. J Clean Prod 2018;190:712–21. https://doi.org/10.1016/j.jclepro.2018.04.159.

[55] Weissbrod I, Bocken NMP. Developing sustainable business experimentation capability – A case study. J Clean Prod 2017;142:2663–76. https://doi.org/10.1016/j.jclepro.2016.11.009.

[56] Bocken N. Business Models for Sustainability. SSRN Forthcoming.

[57] Cabot MI, Luque A, De Las Heras A, Aguayo F. Aspects of sustainability and design engineering for the production of interconnected smart food packaging. PloS One 2019;14:e0216555.

[58] Ragazzi I, Farley M, Jeffery K, Butnar I. Using life cycle assessments to guide reduction in the carbon footprint of single-use lab consumables. PLOS Sustain Transform 2023;2:e0000080. https://doi.org/10.1371/journal.pstr.0000080.

[59] Fazio S, Biganzoli F, De Laurentiis V, Zampori L, Sala S, Diaconu E. Supporting information to the characterisation factors of recommended EF Life Cycle Impact Assessment methods – Version 2, from ILCD to EF 3.0. Luxembourg: Publications Office of the European Union; 2018. https://doi.org/doi/10.2760/002447.

[60] Lüdeke-Freund F, Gold S, Bocken NMP. A Review and Typology of Circular Economy Business Model Patterns. J Ind Ecol 2019;23:36–61. https://doi.org/10.1111/jiec.12763.

[61] Star SL, Griesemer JR. Institutional Ecology, `Translations' and Boundary Objects: Amateurs and Professionals in Berkeley's Museum of Vertebrate Zoology, 1907-39. Soc Stud Sci 1989;19:387–420. https://doi.org/10.1177/030631289019003001.

Click here to access/download;Figure;Figure_1.png

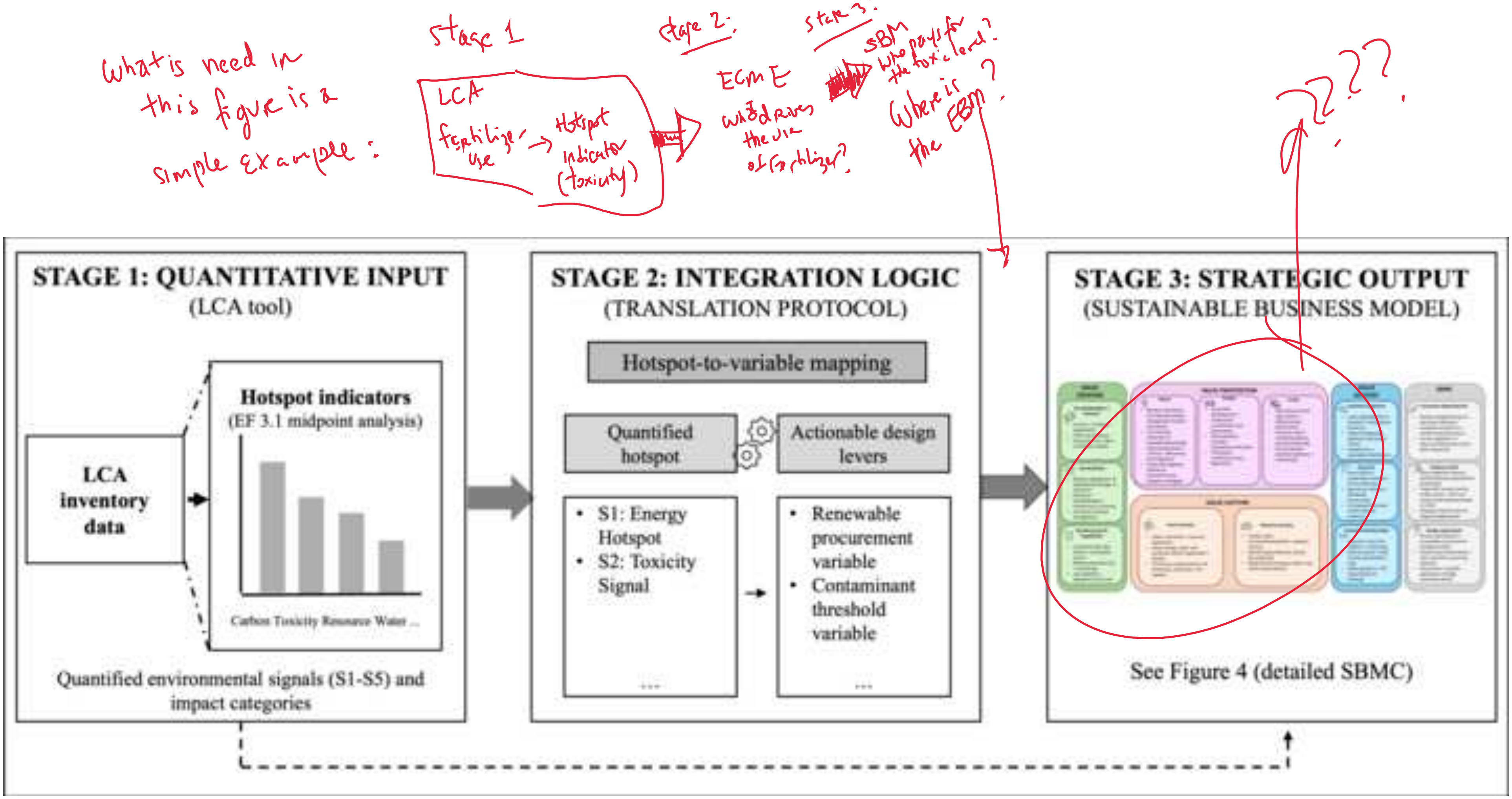

 Click here to access/download;Figure;Figure_2.png

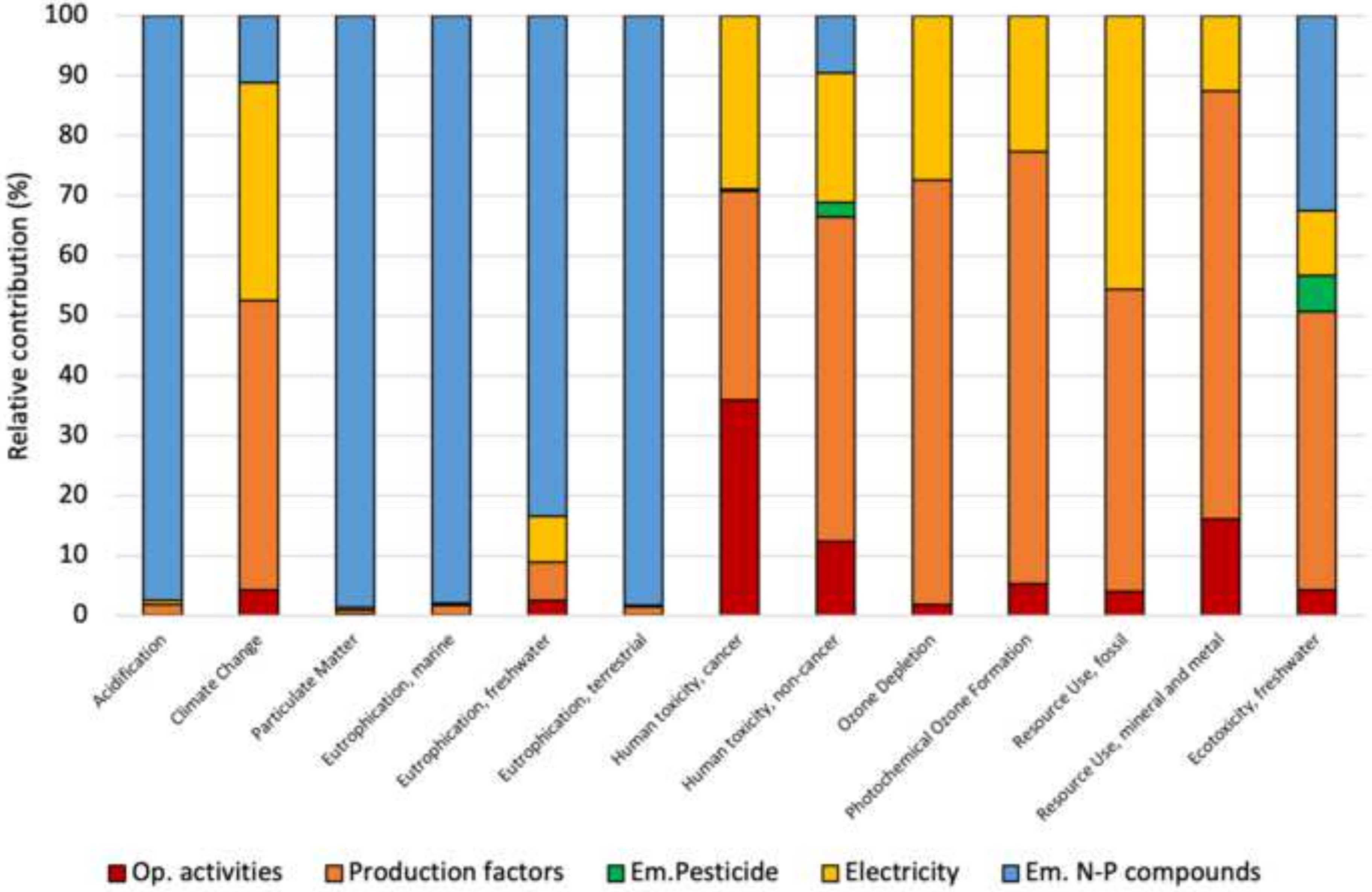

Figure_3

Click here to access/download;Figure;Figure_3.png

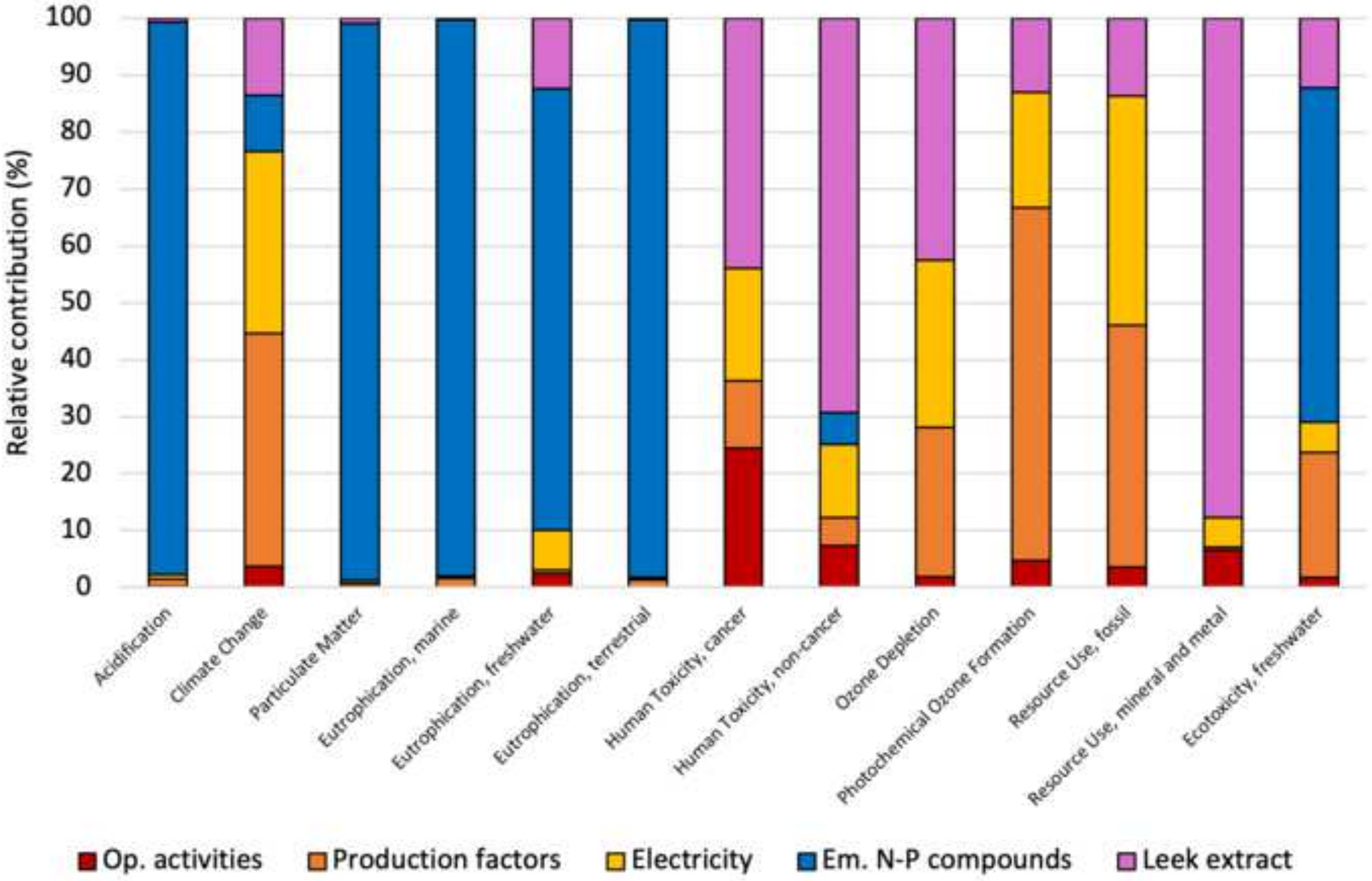

Click here to access/download;Figure;Figure_4.tiff

I finally can see EBME, as a component of SBM?

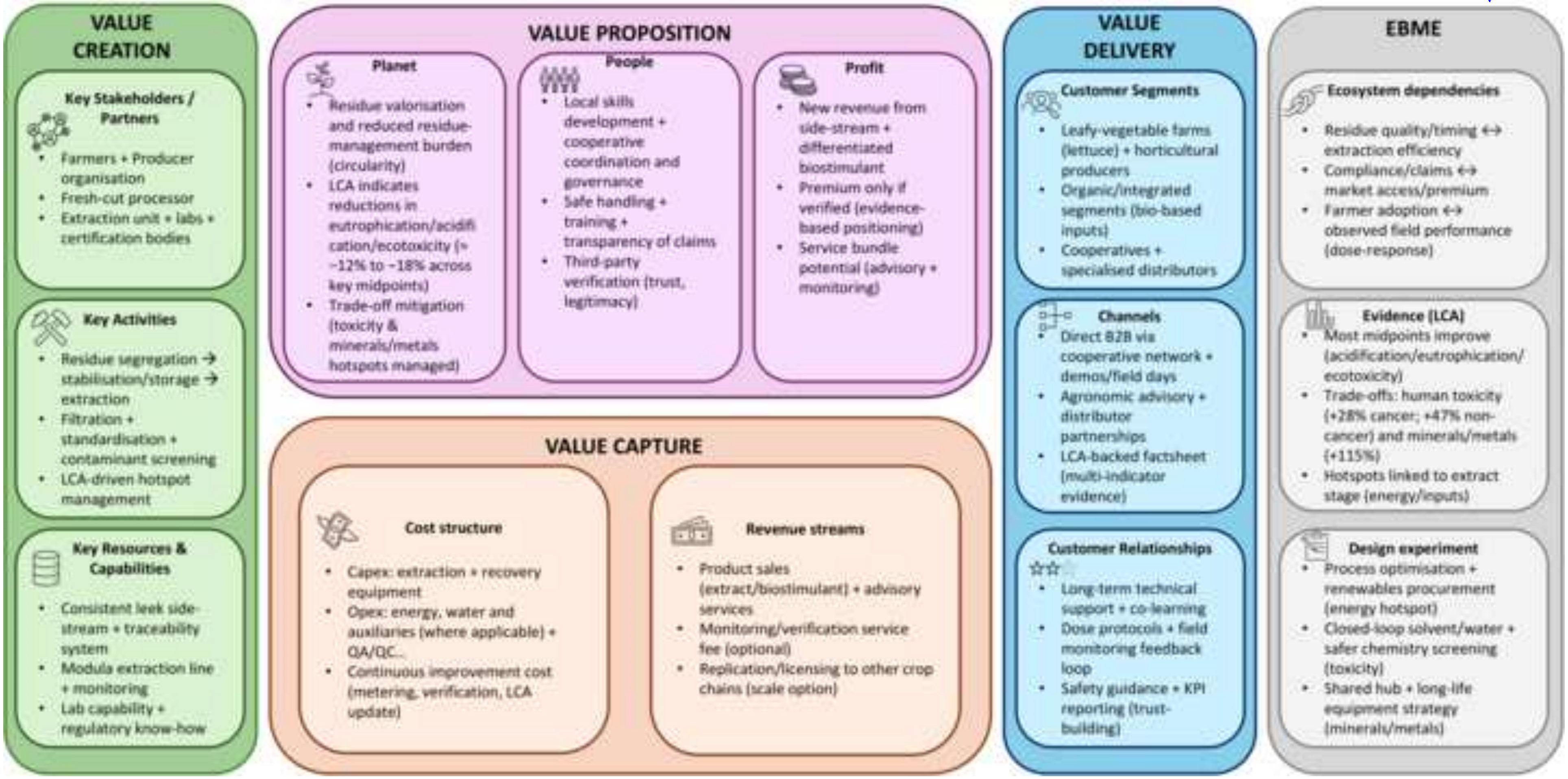

Click here to access/download
Supporting Information
Ethics statement and informed consent.docx

Evidence-based sustainable business model design for agrifood side-stream biostimulants

Federico Zilia[1,*], Luca Ferraro[1], Jacopo Bacenetti[1], Luigi Orsi[1]

[1] Department of Environmental Science and Policy, University of Milan, Milan, Italy

*Corresponding author: federico.zilia@unimi.it

Abstract

Circular bioeconomy initiatives often valorise food-processing residues into bio-based inputs, yet environmental gains depend on how technologies are implemented and governed across multiple actors. This study evaluates a pathway that converts leek processing residues into a bioactive extract used as a plant biostimulant in greenhouse lettuce production and develops an evidence-based procedure to design a sustainable business model around it. We conducted a cradle-to-gate LCA of leek extract production and a comparative LCA of lettuce cultivation for a baseline system and an alternative system applying the extract, using the Environmental Footprint (EF) 3.1 midpoint indicators. The alternative scenario improved most indicators, with consistent reductions in acidification, eutrophication and freshwater ecotoxicity, but trade-offs emerged for toxicity-related indicators and mineral/metal resource use driven by the extract supply chain. We then embedded these multi-impact hotspot signals into an ecosystem-oriented Sustainable Business Model Canvas (SBMC) and an experimentation logic, specifying who must act on each hotspot (processors, extract producers, labs, farmers) and how mitigation and verification can be financed through value capture (product-service bundles, monitoring, and conditional sustainability claims). The approach links LCA evidence to actionable business model choices, supporting credible scaling of residue-based agrifood innovations.

Keywords: food waste; circular bioeconomy; life cycle assessment; sustainable business model.

1. Introduction

Food loss and waste (FLW) is widely recognised as a major sustainability challenge across agri-food systems, with direct implications for climate change, land and water use, eutrophication and biodiversity loss [1–3] At the same time, processing and distribution chains are reconfigured by consumer demand for convenience and safety, expanding fresh-cut and minimally processed 'ready-to-cook' products; these chains concentrate residues spatially and temporally, creating both a waste management burden and a circular bioeconomy opportunity [4,5].

In the European Union, circular economy (CE) policy and bioeconomy strategies increasingly encourage higher-value utilisation of side-streams by applying cascading principles and avoiding low-value downcycling [6,7]. However, scholars warn that circular strategies can generate rebound effects or burden shifting if the energy and material intensity of valorisation outweighs avoided burdens, or if environmental improvements are claimed on narrow indicators such as climate while ignoring toxicity, water scarcity or critical materials [8,9]. These concerns underline the need for multi-impact assessment and transparent accounting when designing circular innovations [10,11].

Plant biostimulants represent a fast-growing category of bio-based agricultural inputs. EU Regulation (EU) 2019/1009 provides a harmonised market framework and a functional definition of biostimulants as products that stimulate plant nutrition processes to improve nutrient use efficiency, stress tolerance, quality traits or nutrient availability, independently of nutrient content [12]. Scientific reviews highlight that biostimulants span diverse categories, including microbial inoculants, humic substances, protein hydrolysates and amino acids, and botanical extracts [13–16]. While biostimulants are often positioned as sustainable alternatives, their net environmental performance is contingent on production pathways and on the extent to which they substitute conventional inputs or stabilise yields under stress [16,17].

Against this background, the SOMMELIER project, led by the University of Milan, develops a circular pathway to valorise leek (*Allium porrum L.*) residues generated in "prima gamma evoluta" processing (an Italian term referring to fresh-cut, minimally processed ready-to-cook vegetables).

In the focal supply chain, leek cultivation covers roughly 25 ha out of 65 ha managed by the producer organisation; processing generates a substantial non-edible fraction (roots, leaves and green bulb parts). While 10-15% of edible fractions are reused, an estimated 30-40% remains under-valorised, corresponding to approximately 225-300 metric tons per year. The project employs pilot-scale Naviglio extraction equipment to obtain bioactive extracts for agricultural use, initially demonstrated in lettuce production.

Life cycle assessment (LCA) is well established for quantifying environmental impacts of products and technologies [18–20]. In circular bioeconomy contexts, LCA is increasingly used to identify hotspots and to compare design options under transparent assumptions [5,21]. Early applications of LCA to horticultural crops have already highlighted the relevance of leek as a case study for assessing environmental performance in agriculture, comparing organic and conventional production systems and identifying fertilisation and energy use as major environmental hotspots [22]. Despite this tool can provides a relevant support for decision making, notably in waste management [23], the LCA alone does not specify how value is created, delivered and captured across multiple actors. Moreover, it does not represent how costs and benefits should be shared, or how environmental hotspots can be financed and governed during scaling [24,25]. Conversely, sustainable business model frameworks provide conceptual tools for designing multi-dimensional value propositions but are often criticised for lacking quantified environmental evidence to guide design priorities [26–28].

This paper primarily targets sustainability and innovation scholars interested in business model design for circular bioeconomy pathways, while using LCA as an enabling decision-support tool rather than as an end in itself.

Despite growing research on circular bioeconomy and sustainable business models, the integration of multi-indicator environmental assessment with business model design remains limited. In particular, existing studies often apply LCA to evaluate environmental impacts, while

sustainable business model frameworks focus on value creation and governance mechanisms, but the methodological link between these two analytical perspectives remains underdeveloped.

Accordingly, this paper responds to this gap by integrating LCA evidence with sustainable business model innovation for circular side-stream valorisation. This study contributes to the literature in three main ways. First, it provides an empirical demonstration of how multi-indicator LCA results can be translated into actionable business model design choices in circular bioeconomy contexts. Second, it proposes a transparent "hotspot-to-canvas" translation protocol that systematically links environmental hotspot drivers to specific Sustainable Business Model Canvas (SBMC) elements, actor responsibilities, and governance mechanisms. Third, it illustrates how environmental assessment can support ecosystem-level business model experimentation, helping actors design financeable mitigation strategies for environmental trade-offs rather than relying on descriptive sustainability claims. Indeed, building on recent work on business model experimentation and Ecologies of Business Models (EBME), we combine this tool with the Sustainable Business Model Canvas (SBMC) to translate quantified LCA hotspots into explicit business model design choices and value capture mechanisms [29–31]. To reduce interpretive inference and make the integration replicable, we apply a transparent "hotspot-to-canvas" translation protocol. Specifically, we *(i)* extract hotspot signals from the EF 3.1 midpoint results and contribution analysis (i.e., impact categories with material differences and their driving processes), *(ii)* assign each hotspot to the responsible SBMC block(s) (activities/resources/costs/partners) and to a concrete intervention hypothesis, *(iii)* specify a measurable implementation variable for each intervention (e.g., kWh L^{-1}, renewable electricity share, solvent/water recirculation rate, contaminant thresholds), and *(iv)* link these variables to governance and value-capture mechanisms (contracts, monitoring services, conditional sustainability claims) so that mitigation is financeable. This protocol is intended to be transferable: any residue-to-input pathway can be analysed by repeating steps once a process-based Life Cycle Inventory (LCI) is available and the downstream use scenario is defined.

The paper addresses three research questions: *(RQ1)* What are the main environmental hotspots and trade-offs of producing leek extract and applying it in lettuce cultivation compared with a baseline scenario? *(RQ2)* How can LCA evidence be operationalised into sustainable business model design using SBMC in a multi-actor ecosystem? *(RQ3)* What managerial, governance and policy implications follow for scaling circular side-stream valorisation credibly without burden shifting?

The manuscript is organized as follows: Section 2 reviews the literature on food waste valorisation, biostimulants, life cycle assessment and sustainable business models for circular bioeconomy pathways. Section 3 describes the case study, the LCA modelling approach and the sustainable business model design procedure. Section 4 reports the LCA and sustainable business model results. Section 5 discusses implications, limitations and future research directions, and Section 6 concludes.

2. Background and related literature

2.1 Circular bioeconomy strategies for agri-food side-streams

FLW valorisation is located at the interface of CE, industrial ecology and the bioeconomy [7,32]. While CE is widely promoted in policy and practice [6,33], academic work emphasises that CE is a contested and multi-interpretation concept with diverse definitions and priorities [9,10]. These conceptual ambiguities matter because they shape what organisations measure and legitimise when implementing circular initiatives [11,34].

In agri-food, cascading and biorefinery perspectives argue that side-streams should be directed to applications with higher functionality and longer value retention, before considering energy recovery [4,5]. Reviews of food manufacturing waste valorisation document options ranging from functional ingredients and biomaterials to composting and anaerobic digestion, with feasibility constrained by contamination, seasonality and logistics [4,5]. Industrial symbiosis scholarship

highlights that the value of side-streams depends on local actor networks and infrastructural proximity, suggesting that circular pathways are ‘place-based’ and governance-intensive [35,36].

However, CE scholars stress the risk of the ‘circular economy rebound’: closing loops can increase total throughput or shift impacts elsewhere, for example through increased processing or transport intensity [8]. Accordingly, circular strategies should be evaluated through multi-impact assessment rather than single-indicator claims [9]. Moreover, governance and contract design are critical because residue quality, segregation and timing strongly influence downstream process efficiency and environmental performance [37,38].

Plant biostimulants are increasingly framed as enabling more resource-efficient and resilient agriculture, potentially contributing to nutrient management, stress tolerance and yield stability [13–15]. The literature emphasises that biostimulants should be defined by their functions rather than solely by nutrient content, recognising that complex mixtures can modulate plant physiological processes [13,16]. Empirical studies report diverse effects across crops and contexts, including improved root development, enhanced nutrient uptake and stress mitigation [16,17].

Yet biostimulants also raise sustainability questions. First, the environmental impacts of producing bio-based inputs depend on extraction pathways, solvents, energy intensity and upstream feedstock logistics [5,21]. Second, agronomic efficacy must be robust across conditions to deliver net system-level benefits; otherwise, additional inputs may add impacts without sufficient yield or substitution gains [16]. Third, regulatory and market legitimacy depend on safety, traceability and claim verification, particularly where complex biological matrices can contain contaminants [12].

Consequently, a sustainability-oriented innovation approach should couple agronomic and technological testing with robust environmental assessment and with business model design that allocates responsibilities, costs and benefits across the actor network [28,29].

2.2 LCA approaches

LCA provides a systematic framework to quantify environmental impacts across life cycle stages, typically in line with ISO 14040/14044 [18–20]. In side-stream valorisation, methodological choices are salient: whether residues are treated as wastes carrying no upstream burdens, or as by-products requiring allocation; how co-products are handled; and whether avoided products should be modelled via system expansion or substitution [39,40]. Because these choices can change results and their interpretation, early-stage studies often adopt attributional approaches with explicit assumptions and sensitivity logic [20,41].

The Environmental Footprint (EF) methods offer a harmonised midpoint indicator set widely used in European contexts [42]. EF indicators enable multi-criteria hotspot analysis, including climate change, acidification, eutrophication, particulate matter, photochemical ozone formation, ozone depletion, human toxicity, freshwater ecotoxicity, resource use (fossil; minerals/metals) and water deprivation [42]. For bio-based extraction processes, toxicity and mineral/metal indicators can be decision-relevant because solvents, catalysts and equipment materials may dominate these categories even when climate performance appears favourable [42,43].

The role of LCA in innovation is increasingly conceptualised as decision support: hotspot identification can guide iterative redesign and reduce the risk of ‘environmental lock-in’ as technologies scale [24]. However, translating hotspots into operational changes requires organisational and inter-organisational mechanisms: who invests, who benefits and how improvements are verified [25].

2.3 Sustainable business models for circularity

SBM research extends conventional business model thinking by integrating environmental and social value creation alongside economic value capture [44,45]. A business model is commonly defined as the system of activities through which an organisation creates and captures value [46,47],

while sustainability-oriented work emphasises that value should be created for a broader set of stakeholders and within ecological limits [28,48,49].

Bocken et al. [29] synthesise SBM archetypes, including ‘create value from waste’, ‘maximise material and energy efficiency’ and ‘substitute with renewables and natural processes’. Circular economy business model pattern research further classifies recurring configurations and highlights barriers for SMEs, including finance, market demand and collaboration challenges [35,40].

Canvases such as the Business Model Canvas proposed by Osterwalder and Pigneur [50] provide an accessible structure for describing business model elements, and sustainability extensions such as the Triple Layered Business Model Canvas (TLBMC) add environmental and social layers [51]. The TLBMC has been valuable for visualising sustainability considerations and supporting ideation, particularly for single organisations or product-service offerings [51–53].

However, circular bioeconomy innovations frequently operate across multiple firms and institutions. Innovation ecosystem research and circular business model studies highlight that successful circularity requires complementary business models, coordination among actors and shared infrastructure [38,54]. The ‘ecologies of business model experimentation’ (EBME) perspective frames business model innovation as experimentation within a system of interacting business models, where synergies and trade-offs must be understood and governed [30,55]. This perspective is particularly suitable for producer-organisation-led projects such as SOMMELIER.

To integrate LCA evidence into business model design, a framework must move beyond listing impacts and instead specify actionable levers, responsibilities and financing mechanisms. While the TLBMC provides a useful layered representation of environmental and social dimensions, applications can remain descriptive and centred on a single focal organisation, with less emphasis on ecosystem coordination and on how environmental improvements are funded through value capture [27,51].

The SBMC proposed by Bocken [56] builds on the logic of value creation, delivery and capture while embedding a Profit-People-Planet value proposition at the core. It is compatible with

stakeholder-oriented sustainability value creation [49] and supports an ecosystem interpretation: the canvas can represent a focal proposition that depends on complementary partner models and governance arrangements [30,38].

Critically, SBMC blocks (key activities, resources, cost structure) provide a direct bridge to LCA hotspots because hotspots are typically associated with specific activities and resource uses [24]. Therefore, in this paper the SBMC is used as the primary business model representation, while the TLBMC is treated as a comparator motivating a move from layered description to ecosystem- and experimentation-oriented design [30,51].

A growing literature seeks to operationalise environmental assessment at the business model level. BM-LCA approaches conceptualise the business model as the object of analysis and compare environmental impacts per unit of economic value, enabling decisions about business configurations [25]. Other strands integrate LCA into early-stage experimentation, emphasising that environmental impacts should be measured and forecasted during pilots to avoid lock-in to high-impact designs [54,55].

Despite these advances, there remains a need for case-based demonstrations that use standard product-focused LCA outputs as inputs to ecosystem-oriented SBM design, producing explicit, financeable redesign levers and governance proposals [26,27]. This work addresses this gap by linking EF midpoint hotspot analysis to SBMC blocks and by articulating how value capture can fund mitigation of trade-offs.

3. Materials and methods

3.1 Study design and system modelling

The environmental performance of a leek-derived biostimulant extract and its application in greenhouse lettuce cultivation was assessed through Life Cycle Assessment (LCA) in accordance with ISO 14040 and ISO 14044 standards [57]. The study was designed as a comparative

assessment of two lettuce production systems: (i) a conventional cultivation scenario (baseline) and (ii) an alternative scenario where the leek-based extract was applied during crop management.

System boundaries were defined according to a cradle-to-gate perspective (resource extraction to factory gate [58]). For lettuce cultivation, the model included upstream production of agricultural inputs (fertilisers, plant protection products, seeds, electricity and fuels), crop establishment, fertilisation, irrigation, crop management, harvesting and on-farm transport, as well as direct emissions to air, soil and water associated with agricultural practices. For the leek extract, the system included collection of leek residues, washing, extraction and thermal treatment steps (including hydrodistillation), and the generation of solid and liquid co-products. Infrastructure, packaging and end-of-life stages were excluded from the analysis.

The environmental burdens associated with extract production were integrated into the alternative lettuce scenario using the application rate adopted in the experimental trials. Impacts related to the production of 1 L of extract were linked to lettuce cultivation by accounting for cultivated area and corresponding crop yield, allowing results to be expressed consistently per kilogram of fresh lettuce.

3.2 Functional units, inventory data and impact assessment

Two functional units (FUs) were defined to reflect the dual objective of the study. The first FU (FU1) was defined as 1 L of leek extract to quantify impacts associated with the production of the biostimulant. The second FU (FU2) was defined as 1 kg of fresh lettuce at the farm gate and represents the reference unit for comparing baseline and alternative cultivation systems.

The LCI was developed using a combination of primary and secondary data. Primary data were collected through direct interviews with farmers using specifically designed questionnaires and from experimental activities, capturing farming operations and the production factors used. The extract application rate was 1 L m^{-2}, and lettuce yields were 1.16 kg m^{-2} for the baseline scenario and 1.33 kg m^{-2} for the alternative scenario with leek extract. The application rate of 1 L m^{-2} was

defined to ensure a uniform distribution of the extract across the cultivated area, following standard agronomic practices for soil-applied liquid biostimulants in greenhouse conditions. This volume was calibrated to achieve sufficient soil moisture for bioactive compound uptake without causing leaching or runoff.

Secondary data were used to model upstream production of agricultural inputs, energy supply, and emissions related to fertiliser use and nutrient cycles, based on established modelling approaches and available databases (e.g., Ecoinvent).

Life Cycle Impact Assessment (LCIA) was performed using the characterisation factors provided by the Environmental Footprint (EF) 3.1 method, the impact assessment method endorsed by the European Commission [59]. The impact following impact categories were considered: climate change, acidification, particulate matter formation, freshwater ecotoxicity, freshwater, terrestrial and marine eutrophication, human toxicity (cancer and non-cancer), ozone depletion, photochemical ozone formation, fossil resource use, and mineral and metal resource use. LCA modelling was carried out using SimaPro software.

3.3 Sustainable business model design

We combine the Ecologies of Business Model Experimentation perspective with Sustainable Business Model Canvas. EBME frames business model innovation as experimentation within a system of interacting business models and is structured here as: (1) sustainability aims; (2) key dependencies and actor mapping; (3) synergies, trade-offs and risks; and (4) design and experimentation priorities.

The SBMC is then used to represent the business model hypothesis in a structured way, integrating value creation, delivery and capture with a Profit-People-Planet value proposition [29,56]. The SBMC is selected over the Triple Layered Business Model Canvas (TLBMC) because *(i)* it supports an ecosystem interpretation suitable for circular supply chains, *(ii)* it ties

environmental levers to activities, resources and value capture (i.e., financing), and *(iii)* it aligns with stakeholder-oriented sustainability value creation [38,49,51].

To clarify the methodological architecture and the interaction between the analytical components used in this study, Fig 1 presents the integrated modelling framework adopted in the research. The framework links environmental modelling and business model design through a structured translation protocol. In this approach, environmental hotspot signals generated by the LCA analysis are translated into operational design variables that inform sustainable business model configuration.

To operationalise integration, we map LCA hotspots to SBMC blocks using a transparent translation logic: each hotspot is associated with one or more intervention hypotheses (e.g., renewable electricity procurement to reduce fossil-related impacts; safer chemistry and closed-loop handling to mitigate toxicity; procurement and equipment durability strategies to address mineral/metal depletion). These hypotheses are incorporated into SBMC blocks and linked to value capture mechanisms that can fund mitigation, consistent with calls to align sustainability performance with economic incentives [27,28].

Operationally, the mapping was performed in five steps: (S1) select a hotspot set by screening EF categories for (a) relevant absolute magnitude for the functional unit and/or (b) meaningful baseline-alternative differences, and confirm drivers via contribution analysis; (S2) trace each hotspot back to the life-cycle stage and inventory items/processes responsible (e.g., electricity, auxiliary materials, field emissions); (S3) translate each driver into one or more business model design levers, and allocate responsibility to the actor(s) controlling that driver (processor, extractor, lab, farm, distributor); (S4) define a measurable decision variable and monitoring approach for each lever (metering, batch QA/QC, third-party verification); and (S5) connect each lever to a financing and governance mechanism within SBMC (cost items, revenue streams, contractual clauses, service components).

Fig 1 visualises this translation logic by showing how quantified environmental signals generated in the LCA stage are progressively transformed into actionable business model variables and design decisions within the SBMC. Reporting these steps makes the approach repeatable for other crops and residue streams, while allowing the specific levers and responsible actors to vary with local ecosystem conditions.

[**Fig 1.** Integrated LCA-SBM framework.]

3.4 Data quality, uncertainty and boundary limitations

Pilot-scale inventories are subject to uncertainty and may not reflect industrial-scale efficiencies; therefore, numerical results are interpreted as indicative hotspot patterns rather than definitive impact values. Omitted stages (e.g., transport, packaging, end-of-life) may become relevant at scale and will need inclusion in future LCA updates [18,19].

Residue status and allocation are recognised as key sources of uncertainty in valorisation LCAs [39,40]. In the pilot modelling, leek residues are treated as a processing side-stream whose upstream cultivation burdens are not assigned to the extract, consistent with cut-off conventions when the residue has low or negative economic value. However, as markets develop, residues may acquire value and could be considered co-products. Future work should therefore test alternative modelling choices including allocation (mass or economic) and system expansion, and conduct sensitivity analysis to support robust decision-making [39,40].

4. Results and discussion

4.1 LCA results

4.1.1 Environmental impacts of leek extract production (FU: 1 L of extract)

The production of 1 L of leek extract was associated with measurable environmental impacts across all the analysed categories, reflecting the experimental and pilot-scale nature of the process.

Impact values were particularly influenced by energy consumption and processing steps required for extraction and thermal treatment.

Table 1 presents the absolute environmental impacts associated with the production of leek extract, distinguishing between water use and extraction processes across multiple impact categories. The results indicate that the extraction phase contributes to all impact categories, while water use is reported only in terms of resource deprivation. Among the reported values, acidification (0.86 mol H^+ eq x 10^{-4}) and ozone depletion (0.82 kg CFC11 eq x10^{-9}) emerge as the most affected impact categories, suggesting that the extraction process carries a notable environmental burden in these areas. Human toxicity impacts are also relevant, with cancer-related toxicity at 0.15 CTUh x10^{-10} and non-cancer toxicity at 0.67 CTUh x10^{-9}, highlighting potential environmental and health considerations associated with the extraction phase. Eutrophication impacts vary across categories, with freshwater eutrophication (0.70 kg P eq x10^{-5}) presenting the highest burden, while marine (0.13 kg N eq x10^{-4}) and terrestrial eutrophication (0.13 mol N eq x10^{-3}) show relatively lower values. Similarly, particulate matter formation (0.79 disease inc. x10^{-9}) and photochemical ozone formation (0.49 kg NMVOC eq x10^{-4}) indicate moderate contributions from the extraction process. In terms of resource use, the extraction process shows an impact of 0.14 MJ for fossil resources and 0.77 kg Sb eq for mineral and metal resources, indicating a measurable resource demand. Notably, water use (0.449 m^3 depriv.) is exclusively attributed to water consumption rather than extraction, while climate change impact (0.11 kg CO_2 eq x10^{-1}) remains minimal, suggesting a relatively low carbon footprint in absolute terms. Overall, the results highlight that the extraction process drives all impact categories, while water use is limited to direct resource deprivation. Acidification, ozone depletion, and human toxicity are among the most significant impact categories, whereas climate change and fossil resource use appear to have a minor contribution.

Table 1. Absolute impacts for the functional unit (1 L of leek-extract)

Impact Categories	Unit of measure	Impact
Acidification	mol H+ eq $x10^{-4}$	0.86
Climate change	kg CO2 eq x 10^{-1}	0.11
Ecotoxicity, freshwater	CTUe	0.11
Particulate matter	disease inc. $x10^{-9}$	0.79
Eutrophication, marine	kg N eq $x10^{-4}$	0.13
Eutrophication, freshwater	kg P eq $x10^{-5}$	0.70
Eutrophication, terrestrial	mol N eq $x10^{-3}$	0.13
Human toxicity, cancer	CTUh $x10^{-10}$	0.15
Human toxicity, non-cancer	CTUh $x10^{-9}$	0.67
Ozone depletion	kg CFC11 eq $x10^{-9}$	0.82
Photochemical ozone formation	kg NMVOC eq $x10^{-4}$	0.49
Resource use, fossils	MJ	0.14
Resource use, minerals and metals	kg Sb eq $x10^{-6}$	0.77
Water use	m3 depriv.	0.500

4.1.2 Environmental impacts of lettuce cultivation (FU: 1 kg of lettuce)

When impacts were expressed per kilogram of lettuce, the alternative cultivation system showed lower values than the baseline in several impact categories (Table 2).

This Table presents the environmental impact values for the two cultivation scenarios, Alternative and Baseline, across multiple impact categories. The results show variations in environmental burdens between the two approaches, with Alternative generally exhibiting lower impacts in most categories. Climate change impact shows a slight reduction, with Alternative (0.603 kg CO_2 eq $x10^{-1}$) performing slightly better than Baseline (0.609 kg CO_2 eq $x10^{-1}$). Similarly, acidification is lower in the alternative scenario (0.954 mol H^+ eq $x10^{-2}$) compared to baseline (1.089 mol H^+ eq $x10^{-2}$). For eutrophication, the results indicate a reduction in all subcategories under Alternative, with marine eutrophication decreasing from 0.372 kg N eq $x10^{-2}$ to 0.325 kg N eq $x10^{-2}$, freshwater eutrophication from 0.456 kg P eq $x10^{-4}$ to 0.428 kg P eq $x10^{-4}$, and terrestrial eutrophication from 0.482 mol N eq $x10^{-1}$ to 0.421 mol N eq $x10^{-1}$. Toxicity-related impacts display mixed trends. Human toxicity, cancer is lower in the baseline scenario (0.202 CTUh $x10^{-10}$) compared to Alternative (0.259 CTUh $x10^{-10}$), whereas human toxicity, non-cancer is higher in the baseline scenario (0.496 CTUh $x10^{-9}$) compared to Alternative (0.729 CTUh $x10^{-9}$). Ecotoxicity in

freshwater follows a similar pattern, with Alternative (0.701 CTUe) showing lower impacts than Baseline (0.817 CTUe). Resource use indicators present marginal differences. Fossil resource use is slightly lower in Alternative (0.744 MJ) compared to Baseline (0.753 MJ), whereas mineral and metal resource use is higher in the alternative scenario (0.663 kg Sb eq $x10^{-6}$ vs. 0.309 kg Sb eq $x10^{-6}$ in the baseline). Finally, water consumption is nearly the same in both scenarios, with Alternative (2.951 m^3 depriv.) and Baseline (2.988 m^3 depriv.) showing minimal variation. The Alternative tends to show lower environmental burdens across most impact categories, with the exception of cancer-related toxicity and mineral resource use, where the baseline scenario performs better. This increase in human toxicity (cancer) and mineral resource use is primarily due to the additional energy-intensive steps and the specific laboratory materials required for the production of the leek extract, which are not required in the traditional baseline scenario. These findings highlight trade-offs between different impact categories, with neither scenario showing a clear overall dominance.

The results of LCA study, for both the considered functional units, cannot be compared with other literature data because no previous LCA studies were carried out on the production of leek extract and its application during lettuce cultivation. Despite this, the achieved results allow the identification of the main contributors to the environmental impact and represent a starting point for a further optimisation of the process.

Table 2. Absolute values related to the functional unit of 1 kg of fresh lettuce.

Impact Categories	Unit of measure	Baseline	Alternative	Δ %
Acidification	mol H+ eq x10^{-2}	1.089	0.954	−12.4%
Climate Change	kg CO2 eq x10^{-1}	0.609	0.603	−1.0%
Ecotoxicity, freshwater	CTUe	0.817	0.701	−14.2%
Particulate Matter	disease inc. x10^{-7}	0.749	0.658	−12.1%
Eutrophication, marine	kg N eq x10^{-2}	0.372	0.325	−12.6%
Eutrophication, freshwater	kg P eq x10^{-4}	0.456	0.428	−6.1%
Eutrophication, terrestrial	mol N eq x10^{-1}	0.482	0.421	−12.7%
Human Toxicity, cancer	CTUh x10^{-10}	0.202	0.259	+28.2%
Human Toxicity, non-cancer	CTUh x10^{-9}	0.496	0.729	+47.0%
Ozone Depletion	kg CFC11 eq x10^{-8}	0.179	0.146	−18.4%
Photochemical Ozone Formation	kg NMVOC eq x10^{-3}	0.291	0.284	−2.4%
Resource Use, fossil	MJ	0.753	0.744	−1.2%
Resource Use, mineral and metal	kg Sb eq x10^{-6}	0.309	0.663	+114.6%
Water Use	m3 depriv.	2.988	2.951	−1.2%

4.1.3 Relative contribution analysis (baseline vs alternative)

Fig 2 presents the relative contribution of different processes to the various impact categories in the LCA study. The results highlight a strong predominance of certain contributions across multiple categories, with some sources overwhelmingly driving specific environmental burdens. Acidification (97.5%), marine eutrophication (97.9%), terrestrial eutrophication (98.3%), and particulate matter formation (98.6%) are almost entirely attributed to emissions from nitrogen and phosphorus compounds (Em. N-P Compounds). Similarly, freshwater eutrophication shows a major contribution from this source (83.5%), although it is more distributed than other eutrophication-related categories. For climate change, the contribution is more distributed, with production factors (48.3%) and electricity (36.3%) being the dominant contributors, while emissions from N-P compounds play a smaller role (11.2%). This contrasts with categories such as human toxicity (cancer and non-cancer), where the contribution is more balanced across different sources. Human toxicity, cancer is primarily influenced by operational activities (35.9%), production factors (34.8%), and electricity use (28.9%), whereas human toxicity, non-cancer is mainly driven by production factors (54.0%), followed by electricity (21.7%) and operational

activities (12.4%). A similar trend is observed in ozone depletion (70.8% from production factors, 27.4% from electricity) and photochemical ozone formation (72.1% from production factors, 22.6% from electricity), indicating a more complex distribution of environmental burdens. Fossil resource use is split between production factors (50.4%) and electricity (45.6%), while mineral and metal resource use follows a similar pattern, with 71.4% from production factors and 12.6% from electricity. Lastly, freshwater ecotoxicity shows a more distributed profile, with production factors (46.4%), emissions from N-P compounds (32.5%), and electricity (10.7%) all playing significant roles. This is one of the few impact categories where emissions from N-P compounds, while still relevant, are not the primary driver. Overall, the results indicate that emissions from nitrogen and phosphorus compounds overwhelmingly drive acidification, particulate matter formation, and eutrophication, while production factors and electricity use dominate climate change, human toxicity, ozone depletion, and resource use categories. The contribution of operational activities remains relevant in specific categories, particularly in human toxicity and resource-related impacts, but its role is generally secondary compared to other factors.

[**Fig 2.** Baseline scenario related to the functional unit of 1 kg of fresh lettuce.]

In addition, Fig 3 shows the relative contribution of different processes to the environmental impact categories in the LCA study. The results highlight distinct variations in contribution patterns across impact categories, with emissions from nitrogen and phosphorus compounds (Em. N-P compounds), production factors, and electricity being the dominant contributors in most cases, while the leek extract plays a relevant role in selected categories. Acidification (97%), marine eutrophication (98%), terrestrial eutrophication (98%), and particulate matter formation (98%) are overwhelmingly driven by emissions from nitrogen and phosphorus compounds, indicating a strong concentration of environmental burdens in these categories. Freshwater eutrophication, while still largely influenced by N-P emissions (78%), shows a more distributed profile, with electricity

(7.1%) and the leek extract (12.4%) contributing to the overall impact. In contrast, climate change impacts are more evenly distributed among sources, with production factors (40.9%) and electricity (32.0%) being the primary contributors, while the leek extract (13.6%) and N-P emissions (10%) also have a notable influence. Similarly, human toxicity (cancer and non-cancer) presents a more complex distribution. Human toxicity, cancer is mainly influenced by the leek extract (44.0%), followed by operational activities (24.5%), electricity (19.7%), and production factors (11.9%). Non-cancer human toxicity exhibits a stronger influence from the leek extract (69.2%), while electricity (12.8%) and production factors (4.95%) contribute to a lesser extent. Ozone depletion (42.5%) and photochemical ozone formation (13.0%) show a notable contribution from the leek extract, while production factors (26.2% and 62.1%, respectively) and electricity (29.4% and 20.2%, respectively) also play significant roles. The use of fossil resources is mainly driven by production factors (42.5%) and electricity (40.3%), while the leek extract (13.7%) contributes a smaller share. Mineral and metal resource use, however, is overwhelmingly dominated by the leek extract (87.8%), making it the most significant factor in this category. Lastly, freshwater ecotoxicity presents a diverse contribution pattern, with production factors (21.9%), N-P emissions (59%), electricity (5.3%), and the leek extract (12.2%) all contributing in varying proportions. Overall, the results indicate that emissions from nitrogen and phosphorus compounds overwhelmingly drive acidification, particulate matter formation, and eutrophication, while production factors and electricity dominate climate change, human toxicity, ozone depletion, and resource use categories. The leek extract plays a significant role in human toxicity (cancer and non-cancer), ozone depletion, and resource use (minerals and metals), highlighting its influence in specific impact categories. These findings suggest that while certain interventions may reduce some environmental burdens, they may also introduce trade-offs in other categories, emphasizing the need for a balanced approach in impact mitigation strategies.

[**Fig 3.** Alternative scenario related to the functional unit of 1 kg of fresh lettuce.]

In this context, it should be noted that the environmental comparison implicitly assumes a substitution logic. The application of the leek extract is intended to improve nutrient use efficiency, theoretically allowing for a reduction in conventional inputs. The net environmental performance of the alternative scenario is therefore contingent on this substitution effect; without a corresponding gain in system efficiency or a reduction in synthetic fertilizers, the biostimulant would represent an additional environmental load rather than a sustainable transition.

4.2 Sustainable business model results

The integrated SBMC-EBME representation is shown in Fig 3, while Table 3 details the business model configuration and the LCA-informed design levers that operationalise hotspot mitigation. While Table 3 specifies how hotspot mitigation is embedded in the SBMC through revenue streams and dedicated investments, the economic feasibility of this logic benefits from an explicit (albeit indicative) quantitative linkage between environmental priorities and incentives. At the current pilot stage, a full techno-economic assessment would be premature; nevertheless, the mitigation strategy can be framed in operational terms by expressing its main enabling costs as a per-batch and per-litre "verification-and-mitigation" component that is directly tied to the hotspots highlighted by the EF 3.1 results. Concretely, the incremental cost of hotspot mitigation is expected to be dominated by *(i)* batch-level QA/QC and contaminant screening that reduce toxicity-related risks and support regulatory legitimacy, *(ii)* metering/traceability and third-party checks that make sustainability claims financeable and credible, and *(iii)* the energy-related cost differential associated with efficiency improvements and low-carbon electricity procurement. These costs can be allocated transparently by dividing batch-level monitoring and testing expenditures by the litres produced per batch, and by adding an energy term calculated from measured kWh per litre multiplied by the applicable electricity price (or price differential for renewable procurement).

This simple accounting makes the incentive link explicit: the “verified sustainability” tier proposed in Table 3 becomes economically meaningful only if the resulting per-litre surcharge remains small relative to the customer-side value of risk reduction (compliance and safety), agronomic performance, and the willingness to pay for verification-backed claims. In this sense, the business model is designed to convert hotspot mitigation from an unfunded aspiration into a priced capability: the same variables that drive the LCA hotspots (e.g., kWh L^{-1}, recovery rates, QA/QC pass rates, contaminant thresholds, minimum effective dose) also define the cost base and the conditions under which a premium tier, service bundle, or performance-linked contract can be offered. By making these relationships explicit, the analysis strengthens the plausibility of the proposed mitigation logic without overstating economic precision at the pilot stage.

The sustainability aims of the project can be summarised as: (A1) valorise under-utilised leek residues and reduce disposal; (A2) create measurable environmental improvements in downstream horticultural production through a bio-based input; (A3) deliver a financially viable model that sustains extraction, compliance and continuous improvement; (A4) support local circular bioeconomy development via skills, jobs and cooperative governance; and (A5) create a replicable pathway for other Alliaceae chains. These aims align with SBM archetypes such as creating value from waste and substituting with renewables and natural processes [29,60].

The valorisation pathway depends on coordinated roles across the ecosystem. Upstream, farmers and the producer organisation ensure crop and residue supply and coordinate agronomic practices. The fresh-cut processor provides segregation and collection of residues and benefits from reduced waste management burdens. Downstream, the extraction operator and technology provider execute extraction and process optimisation. Analytical laboratories provide bioactive profiling and safety testing, essential for regulatory compliance and market legitimacy [12]. Agronomic partners validate efficacy and provide application protocols, while distributors and advisory networks support market access and adoption. Dependencies include feedstock quality (freshness, contamination), timing and storage, energy and water provisioning, solvent handling (where

applicable), traceability and conformity assessment. Such dependencies are consistent with circular innovation ecosystem frameworks emphasising complementarities, shared infrastructure and coordination costs [35,38,54].

The primary synergy is the conversion of a locally concentrated residue stream into a bio-based input that can reduce several environmental indicators in a downstream crop system. The FU2 LCA results provide multi-indicator evidence supporting this synergy, particularly for eutrophication and acidification indicators that are salient in agriculture [3,21]. Nevertheless, the toxicity and mineral/metal trade-offs represent a salient risk of burden shifting. From a systems perspective, such trade-offs can reflect extraction energy mix, auxiliary materials and equipment choices-categories often not visible in climate-only discussions [9]. They also have governance implications: stakeholders must ensure safe handling, avoid contaminants, and adopt procurement and process design that mitigates critical resource dependencies [37,43]. Additional risks include pilot-to-scale uncertainty (industrial efficiencies could change hotspots), regulatory barriers and compliance costs, and adoption uncertainty among farmers. These risks reinforce the value of an experimentation-oriented approach, where environmental and business model hypotheses are tested iteratively [30,55].

Hotspot translation yields a set of redesign levers prioritised for experimentation and governance. For extraction, priorities include: (i) energy profiling and process intensification (reducing kWh per litre), (ii) renewable electricity procurement, (iii) solvent and/or water loop design to reduce toxicity and water deprivation, and (iv) procurement and equipment strategies to reduce mineral/metal impacts. For the downstream crop system, a key lever is determining the minimum effective dose that achieves agronomic benefit with minimal upstream processing per unit of function [16,24]. Experimentation priorities are therefore: (E1) process optimisation trials; (E2) solvent selection and recovery design (aligned with safer-chemistry principles); (E3) water management and reuse options; (E4) quality assurance and standardisation protocols; and (E5) agronomic field trials and dose-response modelling. These levers are incorporated into SBMC

blocks to specify which actors implement them and how investments are financed through value capture [27,28].

Table 3 presents the SBMC for SOMMELIER as an ecosystem-level business model hypothesis. The table explicitly links LCA hotspot signals to business model design levers, addressing a common critique of SBM work-namely, moving from aspirational value propositions to operational and financeable interventions [26,27]. The SBMC emphasises that environmental performance is a joint outcome of operational decisions, governance and incentive alignment across actors [30]. In particular, the LCA-identified trade-offs motivate a hotspot-mitigation-by-design approach: the business model includes commitments to renewable electricity sourcing, closed-loop water/solvent management, safer chemical handling and materials-aware procurement. The value capture logic is structured to fund these commitments through hybrid revenue mechanisms (product + services, monitoring, verification-backed premium claims), consistent with sustainability business model theory [28,49].

Table 3. Sustainable Business Model Canvas applied to SOMMELIER: case configuration and LCA-informed environmental design levers.

**SBMC building block**	**SOMMELIER configuration (what the BM is)**	**LCA-informed environmental levers (readable, measurable)**
Key stakeholders / partners	Producer organisation + farmers; fresh-cut processor; extraction operator; technology provider; analytical labs; agronomic trial partners; conformity bodies; horticultural farms; logistics; public innovation programme.	Partner selection with minimum sustainability conditions: *(i)* extraction site powered mainly by renewables (high renewable share); *(ii)* residue supply agreements specifying quality and timing to avoid extra processing; *(iii)* annual third-party verification of traceability and environmental claims.
Key activities	Residue segregation; storage/stabilisation; extraction (water/ethanol); filtration/standardisation; QA/QC; agronomic formulation & guidance; compliance; marketing & support.	Focus on the LCA hotspots of extraction: *(i)* reduce energy use per litre of extract vs the pilot baseline; *(ii)* adopt water/solvent recirculation where feasible; *(iii)* introduce a safer-chemistry check for auxiliaries to address the toxicity trade-off; *(iv)* update LCA after major process changes.
Key resources & capabilities	Stable residue stream; extraction equipment; lab for bioactives/contaminants; agronomic expertise; regulatory capability; traceability/data; logistics.	Capability built around evidence: *(i)* batch-level metering for energy and water; *(ii)* traceability to support multi-indicator environmental communication; *(iii)* equipment strategy prioritising long lifetime and shared use (hub model) to reduce resource-related burdens over time.
Customer segments	Leafy vegetable farms; organic/integrated; distributors for bio-based inputs; cooperatives seeking circular inputs.	Target customers valuing verified impacts: offer a "verified sustainability" tier only when LCA shows net improvements across multiple indicators and no significant increase in toxicity/resource use; maintain

		a conservative claim strategy to prevent burden shifting.
Channels	Direct B2B via cooperative; demo farms; agronomic advisory; distributor partnerships; innovation showcases.	Evidence-based go-to-market: provide customers with a short environmental factsheet reporting a small set of indicators (e.g., climate change, eutrophication/acidification, toxicity/resource-use flags) and the date of the latest LCA update.
Customer relationships	Technical support & co-learning; performance monitoring; long-term supply; feedback loops; training for safe handling.	Monitoring to protect environmental performance: *(i)* guidance to keep application within recommended dose ("minimum effective dose" principle); *(ii)* seasonal feedback from farms to update performance assumptions; *(iii)* safe handling training to address concerns linked to toxicity categories.
Value proposition – Planet	Valorise leek residues; reduce disposal; improve lettuce performance across multiple EF categories; reduce reliance on conventional inputs where feasible.	Two-part environmental promise: *(i)* preserve improvements in key categories (e.g., eutrophication/acidification/ecotoxicity) shown by the LCA; *(ii)* explicitly manage trade-offs by committing to reduce the toxicity and mineral/metal resource signals through process redesign (energy source, solvent choice, recovery loops), with annual verification.
Value proposition – People	Jobs/skills; cooperative governance; farmer income diversification; compliance/transparency under EU fertilising products regulation.	Measurable co-benefits: training delivered annually; transparent reporting of compliance steps; stakeholder inclusion and fair value sharing, supported by traceability and third-party checks.
Value proposition – Profit	New revenue stream; lower waste handling cost; differentiated biostimulant; sustainability premium; leverage public funding for early investment.	Profit aligned with mitigation: pricing includes a small "verification and mitigation" component covering monitoring, recovery and testing; premiums are conditional on maintaining verified multi-indicator improvements (avoid greenwashing risk).
Cost structure	Capex extraction/recovery; Opex energy/water/solvents/labour; testing/compliance; logistics; advisory.	Cost priorities guided by hotspots: invest first in options that reduce the main burdens (energy, water/solvent use, toxicity-related auxiliaries); treat monitoring and recovery as enabling costs required for verified claims. To make feasibility transparent, key cost items are tracked in allocable units (e.g., € per batch for QA/QC and third-party verification; kWh per litre and €/kWh for energy; CAPEX amortisation per litre in hub configurations).
Revenue streams / value capture	Product sales; advisory & monitoring services; licensing/replication; shared savings with processor & farms.	Value capture linked to evidence: additional revenues (premium/service fees) depend on verified environmental performance; shared-savings contracts align residue quality and process efficiency incentives across the chain. Operationally, the verification-and-mitigation component can be implemented as a per-litre surcharge equal to (QA/QC + monitoring + verification cost per batch)/(litres per batch) plus an energy term based on measured kWh L^{-1}, and applied only to the 'verified' tier.

4.3 Implementation and scaling logic

Implementing the proposed sustainable business model requires explicit decisions on (i) the location and scale of extraction capacity, (ii) residue stabilisation and quality management, and (iii) the governance arrangements that coordinate processors, producers and downstream customers. These choices affect not only economic feasibility but also the system-wide environmental profile, because the LCA results indicate that benefits at crop level coexist with non-negligible trade-offs driven by the extract supply chain and its auxiliary inputs. To test the sensitivity of the proposed

business model and its environmental hotspots to different logistical and scale configurations, two stylised implementation scenarios are therefore discussed as alternative pathways for operationalising the SBMC hypothesis (Table 3) and for interpreting the ecosystem-level design choices represented in Fig 4.

Scenario S1 (decentralised, near-source extraction): This scenario places small modular units close to the fresh-cut processor, thereby reducing residue transport distances and allowing rapid processing of a time-sensitive biomass stream. This configuration can lower logistics burdens and mitigate quality deterioration, potentially reducing the need for stabilisation steps. However, decentralised systems may show higher unit costs and may struggle to achieve high recovery efficiency and robust monitoring at small scale, particularly when advanced solvent/water recirculation or energy-efficiency upgrades require capital-intensive equipment [35]. From a hotspot-mitigation perspective, S1 performs best when paired with operational controls that prevent burden shifting, including stringent residue segregation protocols, minimum effective dose strategies, and performance monitoring that links application guidance to field conditions.

Scenario S2 (regional extraction hub): This scenario aggregates residues from multiple processors into a shared facility, enabling economies of scale in extraction, quality control and utilities management. A hub can justify investments in best-available recovery systems (e.g., closed-loop water/solvent management) and in contracted low-carbon electricity supply, and it can institutionalise third-party verification and traceability as part of routine operations [38,54]. These features align directly with the LCA-informed design levers in Table 3 and can be particularly relevant for managing toxicity- and mineral/metal-related trade-offs through standardised procurement, safer-chemistry screening and longer-lived equipment strategies. The main drawbacks are coordination complexity and the need for governance mechanisms that define residue quality standards, scheduling rules, and transparent benefit sharing among participating actors [30].

Across both scenarios, the SBMC emphasises that environmental performance is a joint outcome of process design and ecosystem governance. Accordingly, Table 3 and Fig 4 should be interpreted as a modular blueprint: the business model building blocks remain stable, while the configuration of stakeholders, cost structure and value capture mechanisms can be adapted to the chosen scaling pathway. In practical terms, S1 prioritises proximity and flexibility, whereas S2 prioritises infrastructure efficiency and measurable hotspot mitigation. These scenario logics provide a decision frame for implementation and for future empirical testing of the business model hypothesis under real-world operating conditions

[**Fig 4.** SBMC-EBME canvas for SOMMELIER]

5. Discussion and managerial implications

SOMMELIER illustrates an empirically common pattern in circular bioeconomy innovations: the potential for improvements in multiple impact categories downstream coupled with trade-offs driven by upstream upgrading [5,8]. The alternative lettuce scenario indicates reductions in eutrophication and acidification-related indicators-highly salient in agriculture-yet significant increases in toxicity and mineral/metal resource use. These results caution against 'circular-equals-sustainable' narratives and reinforce calls for multi-indicator evaluation to avoid burden shifting [9,42]. Transferability beyond the focal case depends primarily on the availability of two key conditions. First, it requires access to a process-based inventory describing the residue upgrading step, since the environmental assessment relies on detailed information about material and energy flows associated with the transformation of waste into a usable input. Second, it requires a clear definition of the downstream function through which environmental and economic benefits are generated, such as reductions in fertiliser or pesticide use, or improvements in yield stability. When these elements are specified, the analytical approach developed in this study can be applied in other contexts following a structured but adaptable logic. In practice, the process involves conducting an

LCA using a harmonised methodology (here the EF 3.1 framework) to identify the environmental hotspots associated with the production pathway. These quantified hotspot drivers can then be systematically translated into specific business model design choices within the Sustainable Business Model Canvas, linking environmental performance to operational decisions, actor responsibilities, and governance arrangements.

Importantly, the approach also emphasises the need to progressively integrate operational data as the pathway moves from experimental to scaled implementation. For this reason, the methodological section clarifies which variables should be systematically monitored in future applications, including energy requirements per unit of output, material recovery rates, the minimum effective agronomic dose, and quality assurance thresholds. Tracking these parameters over time allows both the LCA results and the associated business model assumptions to be updated as empirical evidence accumulates. In this way, the proposed LCA-business model integration is not limited to the specific case analysed here, but offers a replicable framework that can support the design and evaluation of similar residue-to-input valorisation pathways in other agri-food contexts.

For biostimulants specifically, toxicity hotspots are governance-relevant because legitimacy and compliance depend on safety and traceability [12,13]. As such, the business model must not only aim to valorise residues but also embed protocols, monitoring and third-party verification that address these hotspots [47]. For example, this may include batch-level contaminant screening performed by accredited laboratories and digital traceability systems combined with third-party certification of environmental performance claims.

From a theoretical perspective, this study contributes to the emerging interface between environmental assessment and SBM research by demonstrating how quantified LCA outputs can be operationalised within an ecosystem-oriented business model design process. Rather than treating environmental assessment as a separate analytical exercise, the proposed approach embeds LCA hotspot information directly into business model configuration and experimentation. The

SBMC structure ensures that hotspots are mapped to specific activities, resources and cost drivers, and that responsibilities and financing mechanisms are made explicit [30,56].

The EBME lens highlights that the focal SBMC depends on complementary business models- residue segregation at the processor, extraction services, laboratory testing, agronomic advisory networks and farm adoption [38]. In such ecologies, sustainability outcomes emerge from the alignment and co-evolution of multiple models rather than from a single firm-level optimisation. LCA-informed design levers can act as 'boundary objects' that different actors can mobilise during experimentation and governance [55,61].

Compared with the TLBMC, which separates economic, environmental and social layers, the SBMC integrates these dimensions and keeps value capture central [56]. This integration matters when environmental improvements require investment: specifying how value is captured to finance hotspot mitigation reduces the risk that sustainability remains aspirational or underfunded [27].

Three managerial implications follow. First, extraction-stage optimisation should be prioritised. Energy efficiency and renewable electricity procurement can influence multiple impact categories and stabilise operating costs, consistent with SBM archetypes focusing on efficiency and renewable [29].

Second, toxicity and critical resource hotspots require proactive management through safer chemistry screening, closed-loop handling, supplier requirements and monitoring. These actions should be embedded as capabilities (resources) rather than treated as compliance overhead, because they enable legitimacy and premium positioning [49].

Third, value capture mechanisms should be designed to fund mitigation. From an implementation standpoint, this implies reporting at least a small set of economic parameters alongside hotspot metrics - such as testing/verification cost per batch, litres per batch, and energy cost per litre - so that the feasibility of mitigation can be assessed as the pathway scales and business model assumptions are updated with operational data.

Hybrid models combining product sales with services (advisory, monitoring) and performance-based contracts can align incentives and finance continuous improvement [27,28]. For cooperatives, residue contracts should specify quality and timing and include incentives for segregation and freshness to reduce processing intensity and losses [54].

Scaling decisions (decentralised vs hub) should consider not only cost but also the capacity to mitigate hotspots. Shared hubs may justify investments in recovery infrastructure, whereas decentralised units may reduce transport and spoilage. These choices reflect the broader circular supply chain challenge of aligning technical efficiency with governance complexity [35,54].

Innovation programmes and policymakers can increase the credibility of circular bioeconomy support by requiring LCA-informed business model experimentation deliverables [24]. Funding criteria could include hotspot mitigation plans, verification strategies and periodic LCA updates using operational data, rather than prototype demonstrations alone [25].

Public support can also target enabling infrastructures (renewable energy access, water services, shared extraction hubs, accredited testing laboratories) that lower the cost of hotspot mitigation for SMEs and cooperatives [7,35]. In parallel, regulatory frameworks such as Regulation (EU) 2019/1009 can reduce market uncertainty but impose compliance costs; targeted support for conformity assessment may accelerate adoption while safeguarding safety and transparency [12].

6. Conclusions

This paper developed and demonstrated an LCA-informed sustainable business model innovation procedure for circular bioeconomy pathways and illustrated it through the SOMMELIER project valorising leek processing residues into bioactive biostimulant extracts. The comparative LCA indicates that the alternative lettuce scenario reduces most EF midpoint indicators, particularly eutrophication- and acidification-related impacts, suggesting environmental value creation potential. At the same time, significant trade-offs emerge for toxicity and mineral/metal resource use, emphasising the risk of burden shifting and the need for targeted mitigation.

By integrating EBME with SBMC, we translated quantified hotspots into explicit business model design levers and clarified how value capture mechanisms can finance mitigation. Practically, the approach supports cooperatives and processors in prioritising extraction optimisation, safer chemistry and recovery loops, and in designing contracts and pricing schemes that support verified sustainability.

For policymakers and innovation programmes, the case illustrates the value of requiring LCA-informed business model experimentation deliverables to increase the probability that pilots lead to scalable and credible circular solutions.

The study has some limitations. First, the LCA is based on pilot-scale modelling with limited inventory detail and truncated system boundaries for the extract; transport, packaging and end-of-life may become non-negligible at scale.

Second, key LCA methodological choices-residue status, allocation, substitution assumptions-can change results. Future work should test alternative allocation and system expansion rules and quantify uncertainty.

Third, agronomic efficacy evidence is embedded in the scenario comparison but not presented as a full agronomic statistical study; scaling will require multi-season, multi-site trials to confirm dose-response relationships and stability across conditions.

Fourth, this paper focuses primarily on environmental aspects; social impacts are discussed conceptually but could be strengthened through complementary assessments and stakeholder research.

Future research should extend the approach along four lines. (1) Scaling scenarios: model industrial-scale extraction with alternative energy mixes and recovery efficiencies to test whether pilot hotspots persist or diminish. (2) Consequential modelling: explore substitution effects and market-mediated impacts, including potential rebounds. (3) Integrated techno-economic and BM-level assessment: combine LCA with cost modelling and business model LCA approaches to compare configurations per unit of economic value. (4) Governance and adoption research: analyse

how cooperatives allocate benefits, how verification influences willingness to pay and adoption, and how policy instruments affect business model choices.

Methodologically, developing standard ‘hotspot-to-canvas’ mapping protocols and testing them across residue-to-input pathways could generate more generalisable design principles for LCA-informed sustainable business model experimentation.

Beyond the specific SOMMELIER case, the study also provides practical insights for managers and cooperative organisations engaged in circular bioeconomy initiatives. The findings suggest that environmental assessment can play a strategic role in guiding business model design rather than being used only as an ex-post evaluation tool. In practice, this implies that actors involved in residue valorisation pathways should integrate environmental hotspot analysis early in the innovation process and use it to prioritise operational improvements, supplier requirements and investment decisions. For instance, extraction-stage optimisation, renewable energy sourcing, and transparent monitoring systems emerge as managerial priorities that can simultaneously reduce environmental burdens and strengthen market credibility.

More broadly, the proposed integration between LCA evidence and sustainable business model design offers a structured decision-support approach for organisations seeking to scale residue-based innovations while avoiding unintended environmental trade-offs. By explicitly linking environmental performance indicators to governance mechanisms and value capture strategies, the framework can help managers design circular business models that remain environmentally credible as they move from pilot experimentation to commercial implementation.

Author Contributions

Conceptualization: Zilia, F.; Orsi, L.

Methodology: Zilia, F., Ferraro, L., Bacenetti, J.

Supervision: Orsi, L., Bacenetti, J.

Writing – original draft: Zilia, F., Ferraro, L.

Writing – review & editing: All authors

Data Availability Statement

The data supporting the findings of this study are available from the corresponding author upon reasonable request.

Conflicts of Interest

Federico Zilia serves as an Academic Editor for PLOS ONE. To avoid any potential conflict of interest, we request that he is not involved in the handling of this submission. This does not alter our adherence to PLOS ONE policies on sharing data and materials.

References

[1] FAO. Global food losses and food waste – Extent, causes and prevention. FAO; 2011.

[2] Foley JA, Ramankutty N, Brauman KA, Cassidy ES, Gerber JS, Johnston M, et al. Solutions for a cultivated planet. Nature 2011;478:337–42. https://doi.org/10.1038/nature10452.

[3] Notarnicola B, Tassielli G, Renzulli PA, Castellani V, Sala S. Environmental impacts of food consumption in Europe. Eco-Effic Agric Food Syst Sel Pap Addressing Glob Chall Food Syst Those Present Conf "LCA Feed Planet Energy Life" 6-8 Oct 2015 Stresa Milan Expo Italy 2017;140:753–65. https://doi.org/10.1016/j.jclepro.2016.06.080.

[4] Girotto F, Alibardi L, Cossu R. Food waste generation and industrial uses: A review. Urban Min 2015;45:32–41. https://doi.org/10.1016/j.wasman.2015.06.008.

[5] Mirabella N, Castellani V, Sala S. Current options for the valorization of food manufacturing waste: a review. J Clean Prod 2014;65:28–41. https://doi.org/10.1016/j.jclepro.2013.10.051.

[6] Haukkala T. The EU Circular Economy Action Plan: Key Assumptions and Strategic Risks. LIEPP Policy Brief 2025;79.

[7] Stephenson PJ, Damerell A. Bioeconomy and Circular Economy Approaches Need to Enhance the Focus on Biodiversity to Achieve Sustainability. Sustainability 2022;14:10643. https://doi.org/10.3390/su141710643.

[8] Zink T, Geyer R. Circular Economy Rebound. J Ind Ecol 2017;21:593–602. https://doi.org/10.1111/jiec.12545.

[9] Kirchherr J, van Santen R. Research on the circular economy: A critique of the field. Resour Conserv Recycl 2019;151:104480. https://doi.org/10.1016/j.resconrec.2019.104480.

[10] Kirchherr J, Reike D, Hekkert M. Conceptualizing the circular economy: An analysis of 114 definitions. Resour Conserv Recycl 2017;127:221–32. https://doi.org/10.1016/j.resconrec.2017.09.005.

[11] Blomsma F, Brennan G. The Emergence of Circular Economy: A New Framing Around Prolonging Resource Productivity. J Ind Ecol 2017;21:603–14. https://doi.org/10.1111/jiec.12603.

[12] European Union. Regulation (EU) 2019/1009 of the European Parliament and of the Council of 5 June 2019 laying down rules on the making available on the market of EU fertilising products … and repealing Regulation (EC) No 2003/2003 2019.

[13] du Jardin P. Plant biostimulants: Definition, concept, main categories and regulation. Biostimulants Hortic 2015;196:3–14. https://doi.org/10.1016/j.scienta.2015.09.021.

[14] Calvo P, Nelson L, Kloepper JW. Agricultural uses of plant biostimulants. Plant Soil 2014;383:3–41. https://doi.org/10.1007/s11104-014-2131-8.

[15] Yakhin OI, Lubyanov AA, Yakhin IA, Brown PH. Biostimulants in Plant Science: A Global Perspective. Front Plant Sci 2017;Volume 7-2016. https://doi.org/10.3389/fpls.2016.02049.

[16] Rouphael Y, Colla G. Editorial: Biostimulants in Agriculture. Front Plant Sci 2020;Volume 11-2020. https://doi.org/10.3389/fpls.2020.00040.

[17] Bulgari R, Franzoni G, Ferrante A. Biostimulants Application in Horticultural Crops under Abiotic Stress Conditions. Agronomy 2019;9:306. https://doi.org/10.3390/agronomy9060306.

[18] ISO T. 207 (2006) ISO 14040: 2006 environmental management—life cycle assessment—principles and framework. ISO Switz 2006.

[19] ISO E. 14044 2018 Environmental management–Life cycle assessment–Requirements and guidelines (ISO 14044: 2006+ Amd 1: 2017). Ger Version EN ISO 14044 2006 A1 2018.

[20] Rebitzer G, Ekvall T, Frischknecht R, Hunkeler D, Norris G, Rydberg T, et al. Life cycle assessment: Part 1: Framework, goal and scope definition, inventory analysis, and applications. Environ Int 2004;30:701–20. https://doi.org/10.1016/j.envint.2003.11.005.

[21] van der Werf HMG, Knudsen MT, Cederberg C. Towards better representation of organic agriculture in life cycle assessment. Nat Sustain 2020;3:419–25. https://doi.org/10.1038/s41893-020-0489-6.

[22] de Backer E, Aertsens J, Vergucht S, Steurbaut W. Assessing the ecological soundness of organic and conventional agriculture by means of life cycle assessment (LCA): A case study of leek production. Br Food J 2009;111:1028–61. https://doi.org/10.1108/00070700910992916.

[23] Zilia F, Andreottola FG, Orsi L, Parolini M, Bacenetti J. Trash or treasure? A circular business model of recycling plasmix. Circ Econ 2024;3:100089. https://doi.org/10.1016/j.cec.2024.100089.

[24] Geissdoerfer M, Pieroni MPP, Pigosso DCA, Soufani K. Circular business models: A review. J Clean Prod 2020;277:123741. https://doi.org/10.1016/j.jclepro.2020.123741.

[25] Böckin D, Goffetti G, Baumann H, Tillman A-M, Zobel T. Business model life cycle assessment: A method for analysing the environmental performance of business. Sustain Prod Consum 2022;32:112–24. https://doi.org/10.1016/j.spc.2022.04.014.

[26] Lüdeke-Freund F, Dembek K. Sustainable business model research and practice: Emerging field or passing fancy? J Clean Prod 2017;168:1668–78. https://doi.org/10.1016/j.jclepro.2017.08.093.

[27] Evans S, Vladimirova D, Holgado M, Van Fossen K, Yang M, Silva EA, et al. Business Model Innovation for Sustainability: Towards a Unified Perspective for Creation of Sustainable Business Models. Bus Strategy Environ 2017;26:597–608. https://doi.org/10.1002/bse.1939.

[28] Schaltegger S, Lüdeke-Freund F, Hansen EG. Business Models for Sustainability: A Co-Evolutionary Analysis of Sustainable Entrepreneurship, Innovation, and Transformation. Organ Environ 2016;29:264–89. https://doi.org/10.1177/1086026616633272.

[29] Bocken NMP, Short SW, Rana P, Evans S. A literature and practice review to develop sustainable business model archetypes. J Clean Prod 2014;65:42–56. https://doi.org/10.1016/j.jclepro.2013.11.039.

[30] Bocken N, Boons F, Baldassarre B. Sustainable business model experimentation by understanding ecologies of business models. J Clean Prod 2019;208:1498–512. https://doi.org/10.1016/j.jclepro.2018.10.159.

[31] Bocken NMP, Geradts THJ. Barriers and drivers to sustainable business model innovation: Organization design and dynamic capabilities. Long Range Plann 2020;53:101950. https://doi.org/10.1016/j.lrp.2019.101950.

[32] Ghisellini P, Cialani C, Ulgiati S. A review on circular economy: the expected transition to a balanced interplay of environmental and economic systems. Post Foss Carbon Soc Regen Prev Eco-Ind Dev 2016;114:11–32. https://doi.org/10.1016/j.jclepro.2015.09.007.

[33] EMF. Towards the Circular Economy: Economic and business rationale for an accelerated transition. 2013.

[34] Schroeder P, Anggraeni K, Weber U. The Relevance of Circular Economy Practices to the Sustainable Development Goals. J Ind Ecol 2019;23:77–95. https://doi.org/10.1111/jiec.12732.

[35] Rizos V, Behrens A, Van der Gaast W, Hofman E, Ioannou A, Kafyeke T, et al. Implementation of Circular Economy Business Models by Small and Medium-Sized Enterprises (SMEs): Barriers and Enablers. Sustainability 2016;8:1212. https://doi.org/10.3390/su8111212.

[36] Antikainen M, Valkokari K. A Framework for Sustainable Circular Business Model Innovation. Technol Innov Manag Rev 2016;6:5–12. https://doi.org/10.22215/timreview/1000.

[37] Manninen K, Koskela S, Antikainen R, Bocken N, Dahlbo H, Aminoff A. Do circular economy business models capture intended environmental value propositions? J Clean Prod 2018;171:413–22. https://doi.org/10.1016/j.jclepro.2017.10.003.

[38] Konietzko J, Bocken N, Hultink EJ. A Tool to Analyze, Ideate and Develop Circular Innovation Ecosystems. Sustainability 2020;12:417. https://doi.org/10.3390/su12010417.

[39] Weidema B. Avoiding Co-Product Allocation in Life-Cycle Assessment. J Ind Ecol 2000;4:11–33. https://doi.org/10.1162/108819800300106366.

[40] Ekvall T, Weidema BP. System boundaries and input data in consequential life cycle inventory analysis. Int J Life Cycle Assess 2004;9:161–71. https://doi.org/10.1007/BF02994190.

[41] Guinee J. Handbook on Life Cycle Assessment. An Operational Guide to the ISO Standards. Int J Life Cycle Assess 2001;7:311–3. https://doi.org/10.1007/BF02978897.

[42] Zampori L, Pant R. Suggestions for updating the Product Environmental Footprint (PEF) method 2019. https://doi.org/10.2760/424613%2520(online),10.2760/265244%2520(print).

[43] Rosenbaum RK, Bachmann TM, Gold LS, Huijbregts MAJ, Jolliet O, Juraske R, et al. USEtox—the UNEP-SETAC toxicity model: recommended characterisation factors for human toxicity and freshwater ecotoxicity in life cycle impact assessment. Int J Life Cycle Assess 2008;13:532–46. https://doi.org/10.1007/s11367-008-0038-4.

[44] Stubbs W, Cocklin C. Conceptualizing a "Sustainability Business Model." Organ Environ 2008;21:103–27. https://doi.org/10.1177/1086026608318042.

[45] Boons F, Lüdeke-Freund F. Business models for sustainable innovation: state-of-the-art and steps towards a research agenda. Sustain Innov Bus Models 2013;45:9–19. https://doi.org/10.1016/j.jclepro.2012.07.007.

[46] Teece DJ. Business Models, Business Strategy and Innovation. Bus Models 2010;43:172–94. https://doi.org/10.1016/j.lrp.2009.07.003.

[47] Zott C, Amit R, Massa L. The Business Model: Recent Developments and Future Research. J Manag 2011;37:1019–42. https://doi.org/10.1177/0149206311406265.

[48] Upward A, Jones P. An Ontology for Strongly Sustainable Business Models: Defining an Enterprise Framework Compatible With Natural and Social Science. Organ Environ 2016;29:97–123. https://doi.org/10.1177/1086026615592933.

[49] Freudenreich B, Lüdeke-Freund F, Schaltegger S. A Stakeholder Theory Perspective on Business Models: Value Creation for Sustainability. J Bus Ethics 2020;166:3–18. https://doi.org/10.1007/s10551-019-04112-z.

[50] Osterwalder A, Pigneur Y. Business Model Generation: A Handbook for Visionaries, Game Changers, and Challengers. 2010.

[51] Joyce A, Paquin RL. The triple layered business model canvas: A tool to design more sustainable business models. J Clean Prod 2016;135:1474–86. https://doi.org/10.1016/j.jclepro.2016.06.067.

[52] Lozano R. Sustainable business models: Providing a more holistic perspective. Bus Strategy Environ 2018;27:1159–66. https://doi.org/10.1002/bse.2059.

[53] Zilia F, Orsi L, Costantini M, Tedesco DEA, Sugni M. Case study of Life Cycle Assessment and sustainable business model for sea urchin waste. Clean Environ Syst 2023;8:100108. https://doi.org/10.1016/j.cesys.2023.100108.

[54] Geissdoerfer M, Morioka SN, de Carvalho MM, Evans S. Business models and supply chains for the circular economy. J Clean Prod 2018;190:712–21. https://doi.org/10.1016/j.jclepro.2018.04.159.

[55] Weissbrod I, Bocken NMP. Developing sustainable business experimentation capability – A case study. J Clean Prod 2017;142:2663–76. https://doi.org/10.1016/j.jclepro.2016.11.009.

[56] Bocken N. Business Models for Sustainability. SSRN Forthcoming.

[57] Cabot MI, Luque A, De Las Heras A, Aguayo F. Aspects of sustainability and design engineering for the production of interconnected smart food packaging. PloS One 2019;14:e0216555.

[58] Ragazzi I, Farley M, Jeffery K, Butnar I. Using life cycle assessments to guide reduction in the carbon footprint of single-use lab consumables. PLOS Sustain Transform 2023;2:e0000080. https://doi.org/10.1371/journal.pstr.0000080.

[59] Fazio S, Biganzoli F, De Laurentiis V, Zampori L, Sala S, Diaconu E. Supporting information to the characterisation factors of recommended EF Life Cycle Impact Assessment methods – Version 2, from ILCD to EF 3.0. Luxembourg: Publications Office of the European Union; 2018. https://doi.org/doi/10.2760/002447.

[60] Lüdeke-Freund F, Gold S, Bocken NMP. A Review and Typology of Circular Economy Business Model Patterns. J Ind Ecol 2019;23:36–61. https://doi.org/10.1111/jiec.12763.

[61] Star SL, Griesemer JR. Institutional Ecology, `Translations' and Boundary Objects: Amateurs and Professionals in Berkeley's Museum of Vertebrate Zoology, 1907-39. Soc Stud Sci 1989;19:387–420. https://doi.org/10.1177/030631289019003001.

Dear Editor, Dear Reviewers,

We would like to express our sincere gratitude for the time and effort you dedicated to reviewing our manuscript. Your insightful comments and constructive suggestions have been extremely valuable in improving the quality, clarity, and methodological rigor of our work. We have carefully addressed all the points raised by the Reviewers. To facilitate the reading of our revisions, we have adopted the following formatting:

- Our detailed responses to each comment are written in blue.
- The modified or added parts within the manuscript are highlighted in red (and are also clearly indicated in the “tracked changes” version of the revised manuscript).

We hope that these improvements meet your expectations and that the manuscript is now suitable for publication in your prestigious journal.

Best regards.

Reviewer 1

Dear Editor and Authors,

I found the manuscript timely and well aligned with PLOS ONE, especially in how it links LCA evidence to an ecosystem-oriented sustainable business model design logic. In general, the contribution is clear and well structured, but I recommend some revisions to improve transparency and the quality of the work in general.

More precisely, I suggest to:

- Clarify and justify the extract application rate (reported as 1 L m^{-2})

Dear reviewer, thank you for your suggestion. We have updated Section 3.2 to clarify the rationale behind the chosen application rate. The dose of 1 L m^{-2} was selected as it represents a standard volume for soil-drench applications in greenhouse lettuce, ensuring an even distribution of the biostimulant within the root zone while maintaining optimal soil moisture levels for nutrient uptake.

The application rate of 1 L m^{-2} was defined to ensure a uniform distribution of the extract across the cultivated area, following standard agronomic practices for soil-applied liquid biostimulants in greenhouse conditions. This volume was calibrated to achieve sufficient soil moisture for bioactive compound uptake without causing leaching or runoff.

- Add 2 to 3 sentences specifying key background modelling choices (for example, the database version, electricity mix, main emission models) to support reproducibility.

Dear reviewer, thank you for your suggestions. We better specified these aspects in Section 3.2 “Functional units, inventory data and impact assessment”

Life Cycle Impact Assessment (LCIA) was performed using the characterisation factors provided by the Environmental Footprint (EF) 3.1 method, the impact assessment method endorsed by the European Commission [59]. The impact following impact categories were considered: climate change, acidification, particulate matter formation, freshwater ecotoxicity, freshwater, terrestrial and marine eutrophication, human toxicity (cancer and non-cancer), ozone depletion, photochemical ozone formation, fossil resource use, and mineral and metal resource use. LCA modelling was carried out using SimaPro software.

The added reference [59] is the following: Fazio S, Biganzoli F, De Laurentiis V, Zampori L, Sala S, Diaconu E. Supporting information to the characterisation factors of recommended EF Life Cycle Impact Assessment methods – Version 2, from ILCD to EF 3.0. Luxembourg: Publications Office of the European Union; 2018. https://doi.org/doi/10.2760/002447.

- In the comparative results, toxicity and mineral/metal resource use increase in the alternative scenario. You should add a short explicit explanation on it.

We agree with your point. The increase in these specific categories is linked to the “upstream” impacts of the biostimulant production (energy for extraction and lab consumables), which are absent in the baseline. We have added an explicit explanation in Section 4.1.2 to clarify this trade-off.

This increase in human toxicity (cancer) and mineral resource use is primarily due to the additional energy-intensive steps and the specific laboratory materials required for the production of the leek extract, which are not required in the traditional baseline scenario.

- Tables would benefit from quick consistency edits. I suggest you harmonise decimal separators (e.g., 0.11 vs 0,11) and scientific notation formatting across Table 1 and Table 2.

We apologize for these inconsistencies, which resulted from a formatting error during the final assembly of the document. We have now fully revised Table 1 and Table 2 to ensure that decimal separators (using the point “.” as the standard) and scientific notation formatting are consistent throughout the manuscript.

- I suggest you to better explain the sentence in lines 515-517 when you said: “model should “embed protocols, monitoring and third-party verification”. Please add one sentence that names two concrete instruments (so readers immediately see what you mean in practice).

Thank you for the suggestion. We have clarified the sentence by adding concrete examples of operational instruments, such as batch-level contaminant screening conducted by accredited laboratories and digital traceability protocols with third-party certification of environmental claims.

- I recommend you a careful spell-check of the manuscript. Indeed, there are some minor typos and grammatical phrasing that could be smoothed out.

Dear reviewer, thank you for this reminder. We corrected some minor typos.

- I would suggest expanding the Conclusions to more explicitly address future perspectives, particularly regarding the managerial implications derived from the study.

Dear reviewer, thank you for your comment. Following your suggestion, we expanded the Conclusions section to more clearly articulate the managerial implications and future perspectives of the proposed LCA-business model integration framework, particularly regarding its role as a decision-support tool for scaling circular bioeconomy innovations.

- With regard to LCA the selection of the LCIA method should be justified and, if possible, a comparison with previously carried out studies should be introduced into the discussion. If the comparison is not possible due to the lack of studies this aspect should be better underline.

Dear reviewer, thanks for the suggestion. Regarding the LCIA method we have specified in the manuscript that it was selected because it is the one endorsed at European level by the European Commission. About the comparison with previously carried out studies, following your suggestion we have specified that a comparison cannot be drawn because no other LCA studies were carried out on the same topic. In detail, the following sentences were introduced:

The results of LCA study, for both the considered functional units, cannot be compared with other literature data because no previous LCA studies were carried out on the production of leek extract and its application during lettuce cultivation. Despite this, the achieved results allow the identification of the main contributors to the environmental impact and represent a starting point for a further optimisation of the process.

With these moderate revisions and formatting fixes, the paper will be stronger and easier to interpret.

Thank you!

Reviewer 2

- Writing style is acceptable.

Dear reviewer, thank you.

In any case, we have proofread the English to make stylistic improvements, minor corrections, and to fix some typos.

- However, there must be a clearer link between LCA and the Business model. Currently, the relation is interpretive. The article is presented as a practice management reflection. It requires more discussion on broader areas (how is this method transferable? Is this replicable beyond this case?).

Dear reviewer, we agree with your comments and have strengthened both the methodological bridge and the generalisability discussion. In the revised manuscript we now formalise a transparent "hotspot-to-canvas" protocol that translates quantified EF 3.1 LCA hotspot drivers into specific SBMC design levers, actor responsibilities, measurable decision variables, and value-capture/governance mechanisms. Moreover, we explicitly described the step-by-step procedure in Section 3.3 (S1-S5) to reduce interpretive inference and we added a dedicated paragraph in the Discussion clarifying transferability and replicability conditions (what data are needed, which variables should be measured and updated when scaling, and how the protocol can be repeated for other residue-to-input pathways). These additions make the LCA-business model linkage operational rather than reflective and clarify how the approach can be replicated beyond this single case.

- The contribution must be also clearer stated. Be sure to mention what any foreign phrase means, there were a couple of Italian words without their equivalent translation in English.

Dear reviewer, thank you for this helpful suggestion. We have strengthened the contribution statement in the Introduction and clarified the theoretical contribution of the study. In particular, we now explicitly articulate three contributions: (i) demonstrating how multi-indicator LCA results can inform sustainable business model design in circular bioeconomy contexts; (ii) proposing a transparent "hotspot-to-canvas" protocol linking LCA hotspot drivers to SBMC elements and actor responsibilities; and (iii) illustrating how environmental evidence can support ecosystem-level business model experimentation and governance.

In addition, we revised the manuscript to ensure that all foreign expressions are clearly explained. Specifically, the Italian expression "prima gamma evoluta" is now translated as "fresh-cut, minimally processed ready-to-cook vegetables". These changes improve clarity and accessibility for an international readership.

- Explain further the sensitivity analysis in Section 4.

We appreciate the comment. In Section 4.3, we performed a scenario-based sensitivity analysis by comparing two different implementation pathways: Scenario S1 (decentralized, near-source extraction) and Scenario S2 (centralized, shared-hub model). These scenarios serve to test how the environmental hotspots and the business model's viability are sensitive to changes in scale, logistics, and infrastructure efficiency. We have now added a sentence at the beginning of Section 4.3 to explicitly define this comparison as a scenario-based sensitivity assessment.

- Report Toxicities using a benchmark and use other metrics.

Thank you for the comment and suggestion. Although the alternative scenario increases toxicity indicators, due to the additional process of the system, the absolute values remain in the range usually reported for greenhouse vegetable productions, also the contribution analysis indicates that the contribution of these impacts represents a small fraction of the environmental burden per kg of lettuce. The Alternative tends to show lower environmental burdens across most impact categories, with the exception of cancer-related toxicity and mineral resource use, where the baseline scenario performs better. This increase in human toxicity (cancer) and mineral resource use is primarily due to the additional energy-intensive steps and the specific laboratory materials required for the production of the leek extract, which are not required in the traditional baseline scenario. These findings highlight trade-offs between different impact categories, with neither scenario showing a clear overall dominance.

- If they claim to bridge a gap or address a need, they need to make what they are doing more formally stated. Or tone down the claims.

Thank you for this suggestion. We have revised the Introduction to more clearly and formally articulate the research gap addressed by the study. In particular, we clarify that while Life Cycle Assessment is widely used to evaluate environmental impacts of circular bioeconomy innovations, and sustainable business model frameworks provide tools to analyse value creation and governance mechanisms, the methodological integration between these two perspectives remains underdeveloped. The revised manuscript now explicitly states this gap and clarifies how the proposed "hotspot-to-canvas" protocol contributes to bridging it. In addition, we refined the contribution statement in the Discussion section to more clearly position the study within the literature on sustainable business model innovation and environmental assessment.

- The environmental comparison implicitly assumes substitution effects, but the manuscript does not quantify changes in input use. Note: Net Welfare and Environmental performance depend on substitution rather than addition.

We agree with your observation. The environmental benefit of any bio-based input is indeed dependent on its ability to substitute conventional burdens. We have added a clarifying statement at the end of Section 4.1.3 to explicitly address this. We highlight that the “Alternative Scenario” is modelled on a nutrient-efficiency logic where the biostimulant acts as a substitute for potential environmental impacts of traditional intensive practices, and that a net gain is only achieved if this substitution effect is realized.

In this context, it should be noted that the environmental comparison implicitly assumes a substitution logic. The application of the leek extract is intended to improve nutrient use efficiency, theoretically allowing for a reduction in conventional inputs. The net environmental performance of the alternative scenario is therefore contingent on this substitution effect; without a corresponding gain in system efficiency or a reduction in synthetic fertilizers, the biostimulant would represent an additional environmental load rather than a sustainable transition.

- The business model section mentions revenue streams and mitigation investments but doesn’t include price estimates. Without a basic economic analysis it is difficult to asses whether the proposed mitigation strategy is economically feasible. Some quantitative linkage between environmental hotspots and economic incentives would strengthen the analysis.

We agree that the previous version discussed mitigation investments and revenue streams without providing a sufficiently explicit economic linkage. In the revised manuscript we add a short, narrative “economic feasibility check” in the business model results section, clarifying how hotspot mitigation can be operationalised through allocable cost units (e.g., € per batch for QA/QC and verification; kWh per litre for energy) and how these can be transparently translated into a per-litre verification-and-mitigation component for the verified sustainability tier. We also refine Table 3 to include this operational pricing logic and to clarify which measurable economic parameters should be tracked during scaling. Because the SOMMELIER pathway is currently implemented at a pilot stage, providing precise price estimates or a full techno-economic assessment would be premature. For this reason, the revised manuscript focuses on defining the cost drivers and the accounting logic through which mitigation efforts could be financed, rather than presenting definitive market prices. While this is not a full techno-economic assessment, it provides a transparent quantitative bridge between environmental hotspots and economic incentives.

- Very importantly, for a modeling paper: there is no model. I would prefer to have a visualization of what the multiple models being employed look like. And more importantly how the outputs and variables of one section are influencing the others as well as the interaction between them.

Dear reviewer, thank you for this important observation. We agree that, in the previous version, the modelling logic underlying the paper was described textually but not sufficiently visualised, which made the interaction between the analytical components less explicit than intended. To address this point, we have added a new figure in the Methods section (Figure 1) that presents the integrated modelling framework adopted in the study.

The new figure makes explicit the sequential and translational logic connecting the different components of the paper. In particular, it shows how the LCA stage provides quantified environmental outputs in the form of EF 3.1 hotspot indicators and contribution signals; how these outputs are then processed through a hotspot-to-variable translation protocol; and how the resulting decision-relevant variables are subsequently incorporated into the sustainable business model design stage through the SBMC. In this way, the figure clarifies not only what the different modelling components are, but also how the outputs of one stage become inputs for the next one.

We have also revised Section 3.3 to explain this logic more explicitly in the text. The revised manuscript now clarifies that the study combines two complementary modelling perspectives - environmental modelling through LCA and strategic design through the SBMC - and that their interaction is operationalised through a structured five-step translation procedure. This addition was intended precisely to address the reviewer’s concern regarding how variables and outputs from one section influence the others, and to make the overall architecture of the paper more transparent and easier to interpret.

Importantly, our intention is not to claim that the LCA and the business model framework are merged into a single pre-existing formal model. Rather, the contribution of the paper lies in proposing and visualising an integrated modelling framework in which quantified LCA hotspot signals are systematically translated into business model design variables, actor responsibilities, and value capture mechanisms. We believe that this addition substantially strengthens the methodological clarity of the paper.

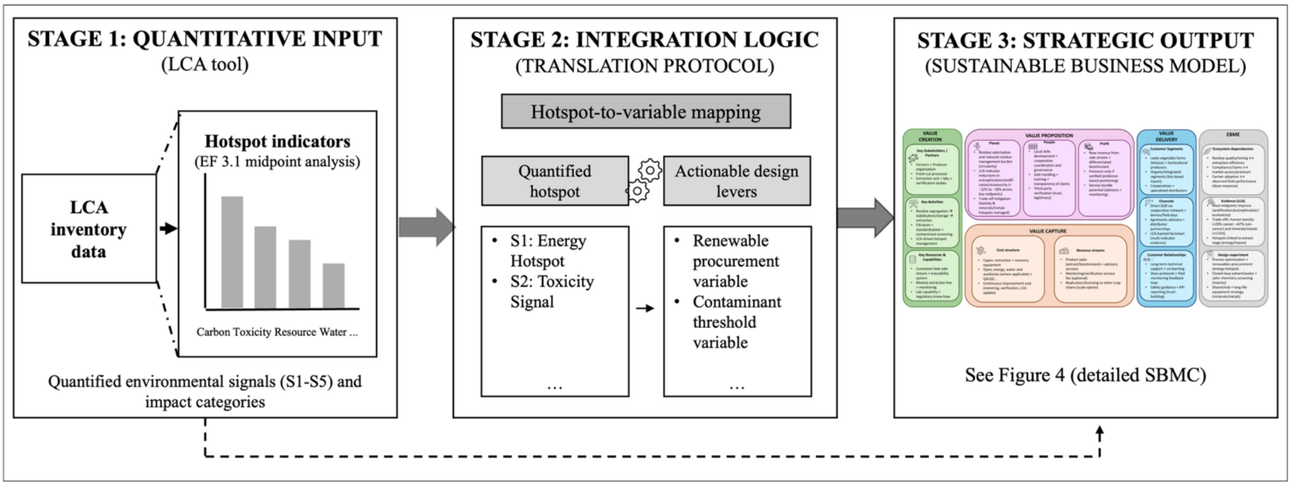

Figure 1. Integrated LCA-SBM framework.
