# Supplementary material for: Evidence-based sustainable business model design for agrifood side-stream biostimulants
Source: PLoS One. 2026 Jul 8;21(7):e0343143. doi: 10.1371/journal.pone.0343143 (PMC13345246; doi:10.1371/journal.pone.0343143)
Supplement: Attachment — Submitted filename: Revision_letter_authors.docx [file pone.0343143.s003.docx]

Dear Editor, Dear Reviewers,

We would like to express our sincere gratitude for the time and effort you dedicated to reviewing our manuscript. Your insightful comments and constructive suggestions have been extremely valuable in improving the quality, clarity, and methodological rigor of our work. We have carefully addressed all the points raised by the Reviewers. To facilitate the reading of our revisions, we have adopted the following formatting:

- Our detailed responses to each comment are written in blue.
- The modified or added parts within the manuscript are highlighted in red (and are also clearly indicated in the “tracked changes” version of the revised manuscript).

We hope that these improvements meet your expectations and that the manuscript is now suitable for publication in your prestigious journal.

Best regards.

**Reviewer 1**

Dear Editor and Authors,

I found the manuscript timely and well aligned with PLOS ONE, especially in how it links LCA evidence to an ecosystem-oriented sustainable business model design logic. In general, the contribution is clear and well structured, but I recommend some revisions to improve transparency and the quality of the work in general.

More precisely, I suggest to:

- Clarify and justify the extract application rate (reported as 1 L m⁻²)

Dear reviewer, thank you for your suggestion. We have updated Section 3.2 to clarify the rationale behind the chosen application rate. The dose of 1 L m⁻² was selected as it represents a standard volume for soil-drench applications in greenhouse lettuce, ensuring an even distribution of the biostimulant within the root zone while maintaining optimal soil moisture levels for nutrient uptake.

The application rate of 1 L m⁻² was defined to ensure a uniform distribution of the extract across the cultivated area, following standard agronomic practices for soil-applied liquid biostimulants in greenhouse conditions. This volume was calibrated to achieve sufficient soil moisture for bioactive compound uptake without causing leaching or runoff.

- Add 2 to 3 sentences specifying key background modelling choices (for example, the database version, electricity mix, main emission models) to support reproducibility.

Dear reviewer, thank you for your suggestions. We better specified these aspects in Section 3.2 “Functional units, inventory data and impact assessment”

Life Cycle Impact Assessment (LCIA) was performed using the characterisation factors provided by the Environmental Footprint (EF) 3.1 method, the impact assessment method endorsed by the European Commission [59]. The impact following impact categories were considered: climate change, acidification, particulate matter formation, freshwater ecotoxicity, freshwater, terrestrial and marine eutrophication, human toxicity (cancer and non-cancer), ozone depletion, photochemical ozone formation, fossil resource use, and mineral and metal resource use. LCA modelling was carried out using SimaPro software.

The added reference [59] is the following: Fazio S, Biganzoli F, De Laurentiis V, Zampori L, Sala S, Diaconu E. Supporting information to the characterisation factors of recommended EF Life Cycle Impact Assessment methods – Version 2, from ILCD to EF 3.0. Luxembourg: Publications Office of the European Union; 2018. https://doi.org/doi/10.2760/002447.

- In the comparative results, toxicity and mineral/metal resource use increase in the alternative scenario. You should add a short explicit explanation on it.

We agree with your point. The increase in these specific categories is linked to the “upstream” impacts of the biostimulant production (energy for extraction and lab consumables), which are absent in the baseline. We have added an explicit explanation in Section 4.1.2 to clarify this trade-off.

This increase in human toxicity (cancer) and mineral resource use is primarily due to the additional energy-intensive steps and the specific laboratory materials required for the production of the leek extract, which are not required in the traditional baseline scenario.

- Tables would benefit from quick consistency edits. I suggest you harmonise decimal separators (e.g., 0.11 vs 0,11) and scientific notation formatting across Table 1 and Table 2.

We apologize for these inconsistencies, which resulted from a formatting error during the final assembly of the document. We have now fully revised Table 1 and Table 2 to ensure that decimal separators (using the point “.” as the standard) and scientific notation formatting are consistent throughout the manuscript.

- I suggest you to better explain the sentence in lines 515-517 when you said: “model should “embed protocols, monitoring and third-party verification”. Please add one sentence that names two concrete instruments (so readers immediately see what you mean in practice).

Thank you for the suggestion. We have clarified the sentence by adding concrete examples of operational instruments, such as batch-level contaminant screening conducted by accredited laboratories and digital traceability protocols with third-party certification of environmental claims.

- I recommend you a careful spell-check of the manuscript. Indeed, there are some minor typos and grammatical phrasing that could be smoothed out.

Dear reviewer, thank you for this reminder. We corrected some minor typos.

- I would suggest expanding the Conclusions to more explicitly address future perspectives, particularly regarding the managerial implications derived from the study.

Dear reviewer, thank you for your comment. Following your suggestion, we expanded the Conclusions section to more clearly articulate the managerial implications and future perspectives of the proposed LCA-business model integration framework, particularly regarding its role as a decision-support tool for scaling circular bioeconomy innovations.

- With regard to LCA the selection of the LCIA method should be justified and, if possible, a comparison with previously carried out studies should be introduced into the discussion. If the comparison is not possible due to the lack of studies this aspect should be better underline.

Dear reviewer, thanks for the suggestion. Regarding the LCIA method we have specified in the manuscript that it was selected because it is the one endorsed at European level by the European Commission. About the comparison with previously carried out studies, following your suggestion we have specified that a comparison cannot be drawn because no other LCA studies were carried out on the same topic. In detail, the following sentences were introduced:

The results of LCA study, for both the considered functional units, cannot be compared with other literature data because no previous LCA studies were carried out on the production of leek extract and its application during lettuce cultivation. Despite this, the achieved results allow the identification of the main contributors to the environmental impact and represent a starting point for a further optimisation of the process.

With these moderate revisions and formatting fixes, the paper will be stronger and easier to interpret.

Thank you!

**Reviewer 2**

- Writing style is acceptable.

Dear reviewer, thank you.

In any case, we have proofread the English to make stylistic improvements, minor corrections, and to fix some typos.

- However, there must be a clearer link between LCA and the Business model. Currently, the relation is interpretive. The article is presented as a practice management reflection. It requires more discussion on broader areas (how is this method transferable? Is this replicable beyond this case?).

Dear reviewer, we agree with your comments and have strengthened both the methodological bridge and the generalisability discussion. In the revised manuscript we now formalise a transparent “hotspot-to-canvas” protocol that translates quantified EF 3.1 LCA hotspot drivers into specific SBMC design levers, actor responsibilities, measurable decision variables, and value-capture/governance mechanisms. Moreover, we explicitly described the step-by-step procedure in Section 3.3 (S1-S5) to reduce interpretive inference and we added a dedicated paragraph in the Discussion clarifying transferability and replicability conditions (what data are needed, which variables should be measured and updated when scaling, and how the protocol can be repeated for other residue-to-input pathways). These additions make the LCA-business model linkage operational rather than reflective and clarify how the approach can be replicated beyond this single case.

- The contribution must be also clearer stated. Be sure to mention what any foreign phrase means, there were a couple of Italian words without their equivalent translation in English.

Dear reviewer, thank you for this helpful suggestion. We have strengthened the contribution statement in the Introduction and clarified the theoretical contribution of the study. In particular, we now explicitly articulate three contributions: (i) demonstrating how multi-indicator LCA results can inform sustainable business model design in circular bioeconomy contexts; (ii) proposing a transparent “hotspot-to-canvas” protocol linking LCA hotspot drivers to SBMC elements and actor responsibilities; and (iii) illustrating how environmental evidence can support ecosystem-level business model experimentation and governance.

In addition, we revised the manuscript to ensure that all foreign expressions are clearly explained. Specifically, the Italian expression “prima gamma evoluta” is now translated as “fresh-cut, minimally processed ready-to-cook vegetables”. These changes improve clarity and accessibility for an international readership.

- Explain further the sensitivity analysis in Section 4.

We appreciate the comment. In Section 4.3, we performed a scenario-based sensitivity analysis by comparing two different implementation pathways: Scenario S1 (decentralized, near-source extraction) and Scenario S2 (centralized, shared-hub model). These scenarios serve to test how the environmental hotspots and the business model’s viability are sensitive to changes in scale, logistics, and infrastructure efficiency. We have now added a sentence at the beginning of Section 4.3 to explicitly define this comparison as a scenario-based sensitivity assessment.

- Report Toxicities using a benchmark and use other metrics.

Thank you for the comment and suggestion. Although the alternative scenario increases toxicity indicators, due to the additional process of the system, the absolute values remain in the range usually reported for greenhouse vegetable productions, also the contribution analysis indicates that the contribution of these impacts represents a small fraction of the environmental burden per kg of lettuce.

The Alternative tends to show lower environmental burdens across most impact categories, with the exception of cancer-related toxicity and mineral resource use, where the baseline scenario performs better. This increase in human toxicity (cancer) and mineral resource use is primarily due to the additional energy-intensive steps and the specific laboratory materials required for the production of the leek extract, which are not required in the traditional baseline scenario. These findings highlight trade-offs between different impact categories, with neither scenario showing a clear overall dominance.

- If they claim to bridge a gap or address a need, they need to make what they are doing more formally stated. Or tone down the claims.

Thank you for this suggestion. We have revised the Introduction to more clearly and formally articulate the research gap addressed by the study. In particular, we clarify that while Life Cycle Assessment is widely used to evaluate environmental impacts of circular bioeconomy innovations, and sustainable business model frameworks provide tools to analyse value creation and governance mechanisms, the methodological integration between these two perspectives remains underdeveloped. The revised manuscript now explicitly states this gap and clarifies how the proposed “hotspot-to-canvas” protocol contributes to bridging it. In addition, we refined the contribution statement in the Discussion section to more clearly position the study within the literature on sustainable business model innovation and environmental assessment.

- The environmental comparison implicitly assumes substitution effects, but the manuscript does not quantify changes in input use. Note: Net Welfare and Environmental performance depend on substitution rather than addition.

We agree with your observation. The environmental benefit of any bio-based input is indeed dependent on its ability to substitute conventional burdens. We have added a clarifying statement at the end of Section 4.1.3 to explicitly address this. We highlight that the “Alternative Scenario” is modelled on a nutrient-efficiency logic where the biostimulant acts as a substitute for potential environmental impacts of traditional intensive practices, and that a net gain is only achieved if this substitution effect is realized.

In this context, it should be noted that the environmental comparison implicitly assumes a substitution logic. The application of the leek extract is intended to improve nutrient use efficiency, theoretically allowing for a reduction in conventional inputs. The net environmental performance of the alternative scenario is therefore contingent on this substitution effect; without a corresponding gain in system efficiency or a reduction in synthetic fertilizers, the biostimulant would represent an additional environmental load rather than a sustainable transition.

- The business model section mentions revenue streams and mitigation investments but doesn’t include price estimates. Without a basic economic analysis it is difficult to asses whether the proposed mitigation strategy is economically feasible. Some quantitative linkage between environmental hotspots and economic incentives would strengthen the analysis.

We agree that the previous version discussed mitigation investments and revenue streams without providing a sufficiently explicit economic linkage. In the revised manuscript we add a short, narrative “economic feasibility check” in the business model results section, clarifying how hotspot mitigation can be operationalised through allocable cost units (e.g., € per batch for QA/QC and verification; kWh per litre for energy) and how these can be transparently translated into a per-litre verification-and-mitigation component for the verified sustainability tier. We also refine Table 3 to include this operational pricing logic and to clarify which measurable economic parameters should be tracked during scaling. Because the SOMMELIER pathway is currently implemented at a pilot stage, providing precise price estimates or a full techno-economic assessment would be premature. For this reason, the revised manuscript focuses on defining the cost drivers and the accounting logic through which mitigation efforts could be financed, rather than presenting definitive market prices. While this is not a full techno-economic assessment, it provides a transparent quantitative bridge between environmental hotspots and economic incentives.

- Very importantly, for a modeling paper: there is no model. I would prefer to have a visualization of what the multiple models being employed look like. And more importantly how the outputs and variables of one section are influencing the others as well as the interaction between them.

Dear reviewer, thank you for this important observation. We agree that, in the previous version, the modelling logic underlying the paper was described textually but not sufficiently visualised, which made the interaction between the analytical components less explicit than intended. To address this point, we have added a new figure in the Methods section (**Figure 1**) that presents the **integrated modelling framework** adopted in the study.

The new figure makes explicit the sequential and translational logic connecting the different components of the paper. In particular, it shows how the **LCA stage** provides quantified environmental outputs in the form of EF 3.1 hotspot indicators and contribution signals; how these outputs are then processed through a **hotspot-to-variable translation protocol**; and how the resulting decision-relevant variables are subsequently incorporated into the **sustainable business model design stage** through the SBMC. In this way, the figure clarifies not only what the different modelling components are, but also how the outputs of one stage become inputs for the next one.

We have also revised Section 3.3 to explain this logic more explicitly in the text. The revised manuscript now clarifies that the study combines two complementary modelling perspectives - environmental modelling through LCA and strategic design through the SBMC - and that their interaction is operationalised through a structured five-step translation procedure. This addition was intended precisely to address the reviewer’s concern regarding how variables and outputs from one section influence the others, and to make the overall architecture of the paper more transparent and easier to interpret.

Importantly, our intention is not to claim that the LCA and the business model framework are merged into a single pre-existing formal model. Rather, the contribution of the paper lies in proposing and visualising an **integrated modelling framework** in which quantified LCA hotspot signals are systematically translated into business model design variables, actor responsibilities, and value capture mechanisms. We believe that this addition substantially strengthens the methodological clarity of the paper.

**Figure 1.** Integrated LCA-SBM framework.
