# Supplementary material for: Evidence-based sustainable business model design for agrifood side-stream biostimulants
Source: PLoS One. 2026 Jul 8;21(7):e0343143. doi: 10.1371/journal.pone.0343143 (PMC13345246; doi:10.1371/journal.pone.0343143)
Supplement: Attachment — Submitted filename: Rebuttal_letter_r2.docx [file pone.0343143.s004.docx]

Dear Editor and Reviewers,

We thank the Academic Editor and the Reviewers for the constructive evaluation of our revised manuscript. We are grateful that both reviewers considered the previous comments addressed and that Reviewer #2 found the manuscript substantially improved and acceptable. In response to the remaining technical suggestions, we have further revised the manuscript to improve concision, practical clarity and alignment between results and implications. We also simplified the integrated LCA-SBM framework figure to make the modelling logic clearer.

# Response to Reviewer #2

- Please reduce redundancy. The paper has too many sections that are repetitive.

We agree and have substantially streamlined the manuscript. Repetitive passages in the Introduction, LCA results, sustainable business model results, Discussion and Conclusions were shortened or removed. In particular, Sections 4.1.1, 4.1.2 and 4.1.3 were rewritten to avoid repeated category-by-category reporting, while Section 4.2 and the Conclusions were condensed to focus on the central LCA-to-business-model contribution.

- Delete sections that do not have connection with the main focus of the paper, i.e., comparison of other methods and why they were not used.

We removed or substantially reduced text that compared alternative business model tools where this was not necessary for the main argument. The revised version keeps only the minimum theoretical justification needed to explain why the SBMC and EBME are appropriate for an ecosystem-oriented, LCA-informed business model design approach.

- Combine results with implications.

We revised the Results and Discussion so that the empirical findings are immediately linked to their implications. The relative contribution analysis now explains how Fig. 2 and Fig. 3 identify the actors and activities responsible for mitigation. Section 4.2 then uses these hotspots to specify operational levers, monitoring needs, value capture mechanisms and scaling choices.

- Eliminate results that only serve as description and instead discuss them. The description of numbers is good for a report, but this is a journal article.

We rewrote the LCA result sections to shift from numerical reporting to interpretation. Values are now reported only when they support a key comparison. The revised text emphasises patterns, trade-offs and hotspot logic, such as the dominance of nutrient-related field emissions in the baseline and the emergence of upstream extract-related burdens in selected categories in the alternative scenario.

- Ecological Business models were not explained well and must be stated what is for.

We clarified the role of EBME in both the Methods and the Discussion. The manuscript now explains that EBME is used to interpret SOMMELIER as a system of interdependent business model roles rather than as a single firm-level model. In practical terms, EBME helps identify how residue segregation by the processor, extract production, laboratory verification, agronomic advisory services and farmer adoption must be aligned for the pathway to function.

- Many of the explanations are abstract, only citing literature. The paper must be more practical. If mentioning an implication in eutrophication, then explain if this is valid in the literature.

We made the discussion more concrete by linking the LCA patterns to practical agricultural and managerial decisions. The text now explains that reductions in acidification and eutrophication are relevant because these categories are closely associated with nutrient-related field emissions in agricultural systems. We also added operational variables that managers can monitor during scaling, including energy use per litre of extract, minimum effective dose, batch-level testing, traceability and verification costs.

- Conclusions are too strong for qualitative analysis: It mentions cancer-related factors as causal. This must be carefully reviewed because many people from medicine can criticize it.

We carefully revised the Abstract, Results, Discussion and Conclusions to avoid any wording that could be interpreted as a medical or causal claim. The manuscript now refers to EF toxicity-related indicators and selected trade-offs rather than cancer-related causal effects. The Conclusions were also softened and shortened and now emphasise the pilot-scale nature of the evidence, methodological limitations and the need for further agronomic and techno-economic validation.

Thank you!
