# Supplementary material for: Evidence-based sustainable business model design for agrifood side-stream biostimulants
Source: PLoS One. 2026 Jul 8;21(7):e0343143. doi: 10.1371/journal.pone.0343143 (PMC13345246; doi:10.1371/journal.pone.0343143)
Supplement: Peer review history

# Peer review: Decision Letter 0

**DOI:** 10.1371/journal.pone.0343143.r001

**Authors:** Alberto Barbaresi

3 Mar 2026

PONE-D-26-03978

Evidence-based sustainable business model design for agrifood side-stream biostimulants

PLOS One

Dear Dr. Zilia,

Thank you for submitting your manuscript to PLOS ONE. After careful consideration, we feel that it has merit but does not fully meet PLOS ONE’s publication criteria as it currently stands. Therefore, we invite you to submit a revised version of the manuscript that addresses the points raised during the review process.

==============================

Both reviewers recognize the relevance and timeliness of the topic, as well as the potential contribution of linking LCA results to sustainable business model design. However, they also highlight several issues that require a careful revision before the manuscript can be considered for publication.

In particular, the connection between the LCA and the business model remains largely interpretative and needs to be more clearly formalized. The paper would benefit from a stronger clarification of its theoretical contribution, a sounder explanation of the modelling approach (including sensitivity analysis and substitution effects), and a clearer representation of how the different components of the framework interact. Moreover, the economic dimension of the proposed business model is currently underdeveloped, which makes it difficult to assess feasibility. Addressing these aspects is essential, especially for a modelling-oriented study.

Reviewer 1 also suggests improvements concerning methodological transparency, justification of choices, clarity of discussion, and language refinement.

Overall, while the manuscript shows promise, substantial revisions are needed to strengthen its conceptual clarity, methodological rigor, and overall coherence. I therefore invite you to submit a thoroughly revised version that carefully addresses all the reviewers’ comments.

==============================

Please submit your revised manuscript by Apr 17 2026 11:59PM. If you will need more time than this to complete your revisions, please reply to this message or contact the journal office at plosone@plos.org. When you're ready to submit your revision, log on to https://www.editorialmanager.com/pone/ and select the 'Submissions Needing Revision' folder to locate your manuscript file.

Please include the following items when submitting your revised manuscript:

- A letter that responds to each point raised by the academic editor and reviewer(s). You should upload this letter as a separate file labeled 'Response to Reviewers'.
- A marked-up copy of your manuscript that highlights changes made to the original version. You should upload this as a separate file labeled 'Revised Manuscript with Track Changes'.
- An unmarked version of your revised paper without tracked changes. You should upload this as a separate file labeled 'Manuscript'.

If you would like to make changes to your financial disclosure, please include your updated statement in your cover letter. Guidelines for resubmitting your figure files are available below the reviewer comments at the end of this letter.

If applicable, we recommend that you deposit your laboratory protocols in protocols.io to enhance the reproducibility of your results. Protocols.io assigns your protocol its own identifier (DOI) so that it can be cited independently in the future. For instructions see: https://journals.plos.org/plosone/s/submission-guidelines#loc-laboratory-protocols. Additionally, PLOS ONE offers an option for publishing peer-reviewed Lab Protocol articles, which describe protocols hosted on protocols.io. Read more information on sharing protocols at https://plos.org/protocols?utm_medium=editorial-email&utm_source=authorletters&utm_campaign=protocols.

We look forward to receiving your revised manuscript.

Kind regards,

Alberto Barbaresi

Academic Editor

PLOS One

Journal Requirements:

When submitting your revision, we need you to address these additional requirements.

1. Please ensure that your manuscript meets PLOS ONE's style requirements, including those for file naming. The PLOS ONE style templates can be found at

https://journals.plos.org/plosone/s/file?id=wjVg/PLOSOne_formatting_sample_main_body.pdf and

https://journals.plos.org/plosone/s/file?id=ba62/PLOSOne_formatting_sample_title_authors_affiliations.pdf

2. Thank you for stating the following in the Acknowledgments Section of your manuscript:

“This work was carried out within the SOMMELIER (Scarti pOrro priMa gaMma EvoLuta bIoattivi pEr l’agRicoltura) project, co-funded by the European Agricultural Fund for Rural Development (EAFRD/FEASR) under Lombardy Region Rural Development Programme (PSR) 2014-2020, Operation 16.2.01 “Pilot projects and development of innovation”. The project was coordinated by AOP UNOLOMBARDIA, in collaboration with the University of Milan and Ortonatura S.A.C.A. R.L., and supported by Regione Lombardia (DG Agriculture).”

We note that you have provided funding information that is not currently declared in your Funding Statement. However, funding information should not appear in the Acknowledgments section or other areas of your manuscript. We will only publish funding information present in the Funding Statement section of the online submission form.

Please remove any funding-related text from the manuscript and let us know how you would like to update your Funding Statement. Currently, your Funding Statement reads as follows:

“This work was carried out within the SOMMELIER (Scarti pOrro priMa gaMma EvoLuta bIoattivi pEr l’agRicoltura) project, co-funded by the European Agricultural Fund for Rural Development (EAFRD/FEASR) under Lombardy Region Rural Development Programme (PSR) 2014-2020, Operation 16.2.01 “Pilot projects and development of innovation”. The project was coordinated by AOP UNOLOMBARDIA, in collaboration with the University of Milan and Ortonatura S.A.C.A. R.L., and supported by Regione Lombardia (DG Agriculture).”

Please include your amended statements within your cover letter; we will change the online submission form on your behalf.

3. We note that the grant information you provided in the ‘Funding Information’ and ‘Financial Disclosure’ sections do not match.

When you resubmit, please ensure that you provide the correct grant numbers for the awards you received for your study in the ‘Funding Information’ section.

4. Thank you for stating the following financial disclosure:

“This work was carried out within the SOMMELIER (Scarti pOrro priMa gaMma EvoLuta bIoattivi pEr l’agRicoltura) project, co-funded by the European Agricultural Fund for Rural Development (EAFRD/FEASR) under Lombardy Region Rural Development Programme (PSR) 2014-2020, Operation 16.2.01 “Pilot projects and development of innovation”. The project was coordinated by AOP UNOLOMBARDIA, in collaboration with the University of Milan and Ortonatura S.A.C.A. R.L., and supported by Regione Lombardia (DG Agriculture).”

Please state what role the funders took in the study.  If the funders had no role, please state: "The funders had no role in study design, data collection and analysis, decision to publish, or preparation of the manuscript."

If this statement is not correct you must amend it as needed.

Please include this amended Role of Funder statement in your cover letter; we will change the online submission form on your behalf.

5. In the online submission form you indicate that your data is not available for proprietary reasons and have provided a contact point for accessing this data. Please note that your current contact point is a co-author on this manuscript. According to our Data Policy, the contact point must not be an author on the manuscript and must be an institutional contact, ideally not an individual. Please revise your data statement to a non-author institutional point of contact, such as a data access or ethics committee, and send this to us via return email. Please also include contact information for the third party organization, and please include the full citation of where the data can be found.

6. Please include your full ethics statement in the ‘Methods’ section of your manuscript file. In your statement, please include the full name of the IRB or ethics committee who approved or waived your study, as well as whether or not you obtained informed written or verbal consent. If consent was waived for your study, please include this information in your statement as well.

7. If the reviewer comments include a recommendation to cite specific previously published works, please review and evaluate these publications to determine whether they are relevant and should be cited. There is no requirement to cite these works unless the editor has indicated otherwise.

[Note: HTML markup is below. Please do not edit.]

Reviewers' comments:

Reviewer's Responses to Questions

**Comments to the Author**

1. Is the manuscript technically sound, and do the data support the conclusions?

The manuscript must describe a technically sound piece of scientific research with data that supports the conclusions. Experiments must have been conducted rigorously, with appropriate controls, replication, and sample sizes. The conclusions must be drawn appropriately based on the data presented.

Reviewer #1: Yes

Reviewer #2: Partly

**********

2. Has the statistical analysis been performed appropriately and rigorously?

Reviewer #1: Yes

Reviewer #2: No

**********

3. Have the authors made all data underlying the findings in their manuscript fully available?

The PLOS Data policy requires authors to make all data underlying the findings described in their manuscript fully available without restriction, with rare exception (please refer to the Data Availability Statement in the manuscript PDF file). The data should be provided as part of the manuscript or its supporting information, or deposited to a public repository. For example, in addition to summary statistics, the data points behind means, medians and variance measures should be available. If there are restrictions on publicly sharing data—e.g. participant privacy or use of data from a third party—those must be specified.

Reviewer #1: Yes

Reviewer #2: No

**********

4. Is the manuscript presented in an intelligible fashion and written in standard English?

PLOS ONE does not copyedit accepted manuscripts, so the language in submitted articles must be clear, correct, and unambiguous. Any typographical or grammatical errors should be corrected at revision, so please note any specific errors here.

Reviewer #1: Yes

Reviewer #2: Yes

**********

5. Review Comments to the Author

Please use the space provided to explain your answers to the questions above. You may also include additional comments for the author, including concerns about dual publication, research ethics, or publication ethics. (Please upload your review as an attachment if it exceeds 20,000 characters)

Reviewer #1: Dear Editor and Authors,

I found the manuscript timely and well aligned with PLOS ONE, especially in how it links LCA evidence to an ecosystem-oriented sustainable business model design logic.

In general, the contribution is clear and well structured, but I recommend some revisions to improve transparency and the quality of the work in general.

More precisely, I suggest to:

Clarify and justify the extract application rate (reported as 1 L m⁻²)

Add 2 to 3 sentences specifying key background modelling choices (for example, the database version, electricity mix, main emission models) to support reproducibility.

In the comparative results, toxicity and mineral/metal resource use increase in the alternative scenario. You should add a short explicit explanation on it.

Tables would benefit from quick consistency edits. I suggest you harmonise decimal separators (e.g., 0.11 vs 0,11) and scientific notation formatting across Table 1 and Table 2.

I suggest you to better explain the sentence in lines 515-517 when you said: “model should “embed protocols, monitoring and third-party verification”. Please add one sentence that names two concrete instruments (so readers immediately see what you mean in practice).

I recommend you a careful spell-check of the manuscript. Indeed, there are some minor typos and grammatical phrasing that could be smoothed out.

I would suggest expanding the Conclusions to more explicitly address future perspectives, particularly regarding the managerial implications derived from the study.

With regard to LCA the selection of the LCIA method should be justified and, if possible, a comparison with previously carried out studies should be introduced into the discussion. If the comparison is not possible due to the lack of studies t8his aspect should be better underline.

With these moderate revisions and formatting fixes, the paper will be stronger and easier to interpret.

Reviewer #2: 1. Writing style is acceptable.

2. However, there must be a clearer link between LCA and the Business model. Currently, the relation is interpretive. The article is presented as a practice management reflection. It requires more discussion on broader areas (how is this method transferable? Is this replicable beyond this case?).

3. The contribution must be also clearer stated. Be sure to mention what any foreign phrase means, there were a couple of Italian words without their equivalent translation in English.

4. Explain further the sensitivity analysis in Section 4.

5. Report Toxicities using a benchmark and use other metrics.

6. If they claim to bridge a gap or address a need, they need to make what they are doing more formally stated. Or tone down the claims.

7. The environmental comparison implicitly assumes substitution effects, but the manuscript does not quantify changes in input use. Note: Net Welfare and Environmental performance depend on substitution rather than addition.

8. The business model section mentions revenue streams and mitigation investments but doesn’t include price estimates. Without a basic economic analysis it is difficult to asses whether the proposed mitigation strategy is economically feasible. Some quantitative linkage between environmental hotspots and economic incentives would strengthen the analysis.

9. Very importantly, for a modeling paper: there is no model. I would prefer to have a visualization of what the multiple models being employed look like. And more importantly how the outputs and variables of one section are influencing the others as well as the interaction between them.

**********

6. PLOS authors have the option to publish the peer review history of their article (what does this mean?). If published, this will include your full peer review and any attached files.

If you choose “no”, your identity will remain anonymous but your review may still be made public.

**Do you want your identity to be public for this peer review?**  For information about this choice, including consent withdrawal, please see our Privacy Policy.

Reviewer #1: No

Reviewer #2: No

**********

[NOTE: If reviewer comments were submitted as an attachment file, they will be attached to this email and accessible via the submission site. Please log into your account, locate the manuscript record, and check for the action link "View Attachments". If this link does not appear, there are no attachment files.]

To ensure your figures meet our technical requirements, please review our figure guidelines: https://journals.plos.org/plosone/s/figures

You may also use PLOS’s free figure tool, NAAS, to help you prepare publication quality figures: https://journals.plos.org/plosone/s/figures#loc-tools-for-figure-preparation.

NAAS will assess whether your figures meet our technical requirements by comparing each figure against our figure specifications.

# Author comment: Author response to Decision Letter 1

**DOI:** 10.1371/journal.pone.0343143.r002

16 Mar 2026

Dear Editor, Dear Reviewers,

We would like to express our sincere gratitude for the time and effort you dedicated to reviewing our manuscript. Your insightful comments and constructive suggestions have been extremely valuable in improving the quality, clarity, and methodological rigor of our work. We have carefully addressed all the points raised by the Reviewers. To facilitate the reading of our revisions, we have adopted the following formatting:

• Our detailed responses to each comment are written in blue.

• The modified or added parts within the manuscript are highlighted in red (and are also clearly indicated in the “tracked changes” version of the revised manuscript).

We hope that these improvements meet your expectations and that the manuscript is now suitable for publication in your prestigious journal.

Best regards.

Reviewer 1

Dear Editor and Authors,

I found the manuscript timely and well aligned with PLOS ONE, especially in how it links LCA evidence to an ecosystem-oriented sustainable business model design logic. In general, the contribution is clear and well structured, but I recommend some revisions to improve transparency and the quality of the work in general.

More precisely, I suggest to:

• Clarify and justify the extract application rate (reported as 1 L m⁻²)

Dear reviewer, thank you for your suggestion. We have updated Section 3.2 to clarify the rationale behind the chosen application rate. The dose of 1 L m⁻² was selected as it represents a standard volume for soil-drench applications in greenhouse lettuce, ensuring an even distribution of the biostimulant within the root zone while maintaining optimal soil moisture levels for nutrient uptake.

The application rate of 1 L m⁻² was defined to ensure a uniform distribution of the extract across the cultivated area, following standard agronomic practices for soil-applied liquid biostimulants in greenhouse conditions. This volume was calibrated to achieve sufficient soil moisture for bioactive compound uptake without causing leaching or runoff.

• Add 2 to 3 sentences specifying key background modelling choices (for example, the database version, electricity mix, main emission models) to support reproducibility.

Dear reviewer, thank you for your suggestions. We better specified these aspects in Section 3.2 “Functional units, inventory data and impact assessment”

Life Cycle Impact Assessment (LCIA) was performed using the characterisation factors provided by the Environmental Footprint (EF) 3.1 method, the impact assessment method endorsed by the European Commission [59]. The impact following impact categories were considered: climate change, acidification, particulate matter formation, freshwater ecotoxicity, freshwater, terrestrial and marine eutrophication, human toxicity (cancer and non-cancer), ozone depletion, photochemical ozone formation, fossil resource use, and mineral and metal resource use. LCA modelling was carried out using SimaPro software.

The added reference [59] is the following: Fazio S, Biganzoli F, De Laurentiis V, Zampori L, Sala S, Diaconu E. Supporting information to the characterisation factors of recommended EF Life Cycle Impact Assessment methods – Version 2, from ILCD to EF 3.0. Luxembourg: Publications Office of the European Union; 2018. https://doi.org/doi/10.2760/002447.

• In the comparative results, toxicity and mineral/metal resource use increase in the alternative scenario. You should add a short explicit explanation on it.

We agree with your point. The increase in these specific categories is linked to the “upstream” impacts of the biostimulant production (energy for extraction and lab consumables), which are absent in the baseline. We have added an explicit explanation in Section 4.1.2 to clarify this trade-off.

This increase in human toxicity (cancer) and mineral resource use is primarily due to the additional energy-intensive steps and the specific laboratory materials required for the production of the leek extract, which are not required in the traditional baseline scenario.

• Tables would benefit from quick consistency edits. I suggest you harmonise decimal separators (e.g., 0.11 vs 0,11) and scientific notation formatting across Table 1 and Table 2.

We apologize for these inconsistencies, which resulted from a formatting error during the final assembly of the document. We have now fully revised Table 1 and Table 2 to ensure that decimal separators (using the point “.” as the standard) and scientific notation formatting are consistent throughout the manuscript.

• I suggest you to better explain the sentence in lines 515-517 when you said: “model should “embed protocols, monitoring and third-party verification”. Please add one sentence that names two concrete instruments (so readers immediately see what you mean in practice).

Thank you for the suggestion. We have clarified the sentence by adding concrete examples of operational instruments, such as batch-level contaminant screening conducted by accredited laboratories and digital traceability protocols with third-party certification of environmental claims.

• I recommend you a careful spell-check of the manuscript. Indeed, there are some minor typos and grammatical phrasing that could be smoothed out.

Dear reviewer, thank you for this reminder. We corrected some minor typos.

• I would suggest expanding the Conclusions to more explicitly address future perspectives, particularly regarding the managerial implications derived from the study.

Dear reviewer, thank you for your comment. Following your suggestion, we expanded the Conclusions section to more clearly articulate the managerial implications and future perspectives of the proposed LCA-business model integration framework, particularly regarding its role as a decision-support tool for scaling circular bioeconomy innovations.

• With regard to LCA the selection of the LCIA method should be justified and, if possible, a comparison with previously carried out studies should be introduced into the discussion. If the comparison is not possible due to the lack of studies this aspect should be better underline.

Dear reviewer, thanks for the suggestion. Regarding the LCIA method we have specified in the manuscript that it was selected because it is the one endorsed at European level by the European Commission. About the comparison with previously carried out studies, following your suggestion we have specified that a comparison cannot be drawn because no other LCA studies were carried out on the same topic. In detail, the following sentences were introduced:

The results of LCA study, for both the considered functional units, cannot be compared with other literature data because no previous LCA studies were carried out on the production of leek extract and its application during lettuce cultivation. Despite this, the achieved results allow the identification of the main contributors to the environmental impact and represent a starting point for a further optimisation of the process.

With these moderate revisions and formatting fixes, the paper will be stronger and easier to interpret.

Thank you!

Reviewer 2

• Writing style is acceptable.

Dear reviewer, thank you.

In any case, we have proofread the English to make stylistic improvements, minor corrections, and to fix some typos.

• However, there must be a clearer link between LCA and the Business model. Currently, the relation is interpretive. The article is presented as a practice management reflection. It requires more discussion on broader areas (how is this method transferable? Is this replicable beyond this case?).

Dear reviewer, we agree with your comments and have strengthened both the methodological bridge and the generalisability discussion. In the revised manuscript we now formalise a transparent “hotspot-to-canvas” protocol that translates quantified EF 3.1 LCA hotspot drivers into specific SBMC design levers, actor responsibilities, measurable decision variables, and value-capture/governance mechanisms. Moreover, we explicitly described the step-by-step procedure in Section 3.3 (S1-S5) to reduce interpretive inference and we added a dedicated paragraph in the Discussion clarifying transferability and replicability conditions (what data are needed, which variables should be measured and updated when scaling, and how the protocol can be repeated for other residue-to-input pathways). These additions make the LCA-business model linkage operational rather than reflective and clarify how the approach can be replicated beyond this single case.

• The contribution must be also clearer stated. Be sure to mention what any foreign phrase means, there were a couple of Italian words without their equivalent translation in English.

Dear reviewer, thank you for this helpful suggestion. We have strengthened the contribution statement in the Introduction and clarified the theoretical contribution of the study. In particular, we now explicitly articulate three contributions: (i) demonstrating how multi-indicator LCA results can inform sustainable business model design in circular bioeconomy contexts; (ii) proposing a transparent “hotspot-to-canvas” protocol linking LCA hotspot drivers to SBMC elements and actor responsibilities; and (iii) illustrating how environmental evidence can support ecosystem-level business model experimentation and governance.

In addition, we revised the manuscript to ensure that all foreign expressions are clearly explained. Specifically, the Italian expression “prima gamma evoluta” is now translated as “fresh-cut, minimally processed ready-to-cook vegetables”. These changes improve clarity and accessibility for an international readership.

• Explain further the sensitivity analysis in Section 4.

We appreciate the comment. In Section 4.3, we performed a scenario-based sensitivity analysis by comparing two different implementation pathways: Scenario S1 (decentralized, near-source extraction) and Scenario S2 (centralized, shared-hub model). These scenarios serve to test how the environmental hotspots and the business model’s viability are sensitive to changes in scale, logistics, and infrastructure efficiency. We have now added a sentence at the beginning of Section 4.3 to explicitly define this comparison as a scenario-based sensitivity assessment.

• Report Toxicities using a benchmark and use other metrics.

Thank you for the comment and suggestion. Although the alternative scenario increases toxicity indicators, due to the additional process of the system, the absolute values remain in the range usually reported for greenhouse vegetable productions, also the contribution analysis indicates that the contribution of these impacts represents a small fraction of the environmental burden per kg of lettuce.

The Alternative tends to show lower environmental burdens across most impact categories, with the exception of cancer-related toxicity and mineral resource use, where the baseline scenario performs better. This increase in human toxicity (cancer) and mineral resource use is primarily due to the additional energy-intensive steps and the specific laboratory materials required for the production of the leek extract, which are not required in the traditional baseline scenario. These findings highlight trade-offs between different impact categories, with neither scenario showing a clear overall dominance.

• If they claim to bridge a gap or address a need, they need to make what they are doing more formally stated. Or tone down the claims.

Thank you for this suggestion. We have revised the Introduction to more clearly and formally articulate the research gap addressed by the study. In particular, we clarify that while Life Cycle Assessment is widely used to evaluate environmental impacts of circular bioeconomy innovations, and sustainable business model frameworks provide tools to analyse value creation and governance mechanisms, the methodological integration between these two perspectives remains underdeveloped. The revised manuscript now explicitly states this gap and clarifies how the proposed “hotspot-to-canvas” protocol contributes to bridging it. In addition, we refined the contribution statement in the Discussion section to more clearly position the study within the literature on sustainable business model innovation and environmental assessment.

• The environmental comparison implicitly assumes substitution effects, but the manuscript does not quantify changes in input use. Note: Net Welfare and Environmental performance depend on substitution rather than addition.

We agree with your observation. The environmental benefit of any bio-based input is indeed dependent on its ability to substitute conventional burdens. We have added a clarifying statement at the end of Section 4.1.3 to explicitly address this. We highlight that the “Alternative Scenario” is modelled on a nutrient-efficiency logic where the biostimulant acts as a substitute for potential environmental impacts of traditional intensive practices, and that a net gain is only achieved if this substitution effect is realized.

In this context, it should be noted that the environmental comparison implicitly assumes a substitution logic. The application of the leek extract is intended to improve nutrient use efficiency, theoretically allowing for a reduction in conventional inputs. The net environmental performance of the alternative scenario is therefore contingent on this substitution effect; without a corresponding gain in system efficiency or a reduction in synthetic fertilizers, the biostimulant would represent an additional environmental load rather than a sustainable transition.

• The business model section mentions revenue streams and mitigation investments but doesn’t include price estimates. Without a basic economic analysis it is difficult to asses whether the proposed mitigation strategy is economically feasible. Some quantitative linkage between environmental hotspots and economic incentives would strengthen the analysis.

We agree that the previous version discussed mitigation investments and revenue streams without providing a sufficiently explicit economic linkage. In the revised manuscript we add a short, narrative “economic feasibility check” in the business model results section, clarifying how hotspot mitigation can be operationalised through allocable cost units (e.g., € per batch for QA/QC and verification; kWh per litre for energy) and how these can be transparently translated into a per-litre verification-and-mitigation component for the verified sustainability tier. We also refine Table 3 to include this operational pricing logic and to clarify which measurable economic parameters should be tracked during scaling. Because the SOMMELIER pathway is currently implemented at a pilot stage, providing precise price estimates or a full techno-economic assessment would be premature. For this reason, the revised manuscript focuses on defining the cost drivers and the accounting logic through which mitigation efforts could be financed, rather than presenting definitive market prices. While this is not a full techno-economic assessment, it provides a transparent quantitative bridge between environmental hotspots and economic incentives.

• Very importantly, for a modeling paper: there is no model. I would prefer to have a visualization of what the multiple models being employed look like. And more importantly how the outputs and variables of one section are influencing the others as well as the interaction between them.

Dear reviewer, thank you for this important observation. We agree that, in the previous version, the modelling logic underlying the paper was described textually but not sufficiently visualised, which made the interaction between the analytical components less explicit than intended. To address this point, we have added a new figure in the Methods section (new Figure 1) that presents the integrated modelling framework adopted in the study.

The new figure makes explicit the sequential and translational logic connecting the different components of the paper. In particular, it shows how the LCA stage provides quantified environmental outputs in the form of EF 3.1 hotspot indicators and contribu

Attachment Submitted filename: Revision_letter_authors.docx

# Peer review: Decision Letter 1

**DOI:** 10.1371/journal.pone.0343143.r003

**Authors:** Alberto Barbaresi, Alberto Barbaresi

15 Apr 2026

PONE-D-26-03978R1

Evidence-based sustainable business model design for agrifood side-stream biostimulants

PLOS One

Dear Dr. Zilia,

Thank you for submitting your manuscript to PLOS ONE. After careful consideration, we feel that it has merit but does not fully meet PLOS ONE’s publication criteria as it currently stands. Therefore, we invite you to submit a revised version of the manuscript that addresses the points raised during the review process.

==============================

The paper is substantially improved. However, before publishing, please address the comments risen but the Reviewer #2

===========

Please submit your revised manuscript by May 30 2026 11:59PM. If you will need more time than this to complete your revisions, please reply to this message or contact the journal office at plosone@plos.org. When you're ready to submit your revision, log on to https://www.editorialmanager.com/pone/ and select the 'Submissions Needing Revision' folder to locate your manuscript file.

Please include the following items when submitting your revised manuscript:

- A letter that responds to each point raised by the academic editor and reviewer(s). You should upload this letter as a separate file labeled 'Response to Reviewers'.
- A marked-up copy of your manuscript that highlights changes made to the original version. You should upload this as a separate file labeled 'Revised Manuscript with Track Changes'.
- An unmarked version of your revised paper without tracked changes. You should upload this as a separate file labeled 'Manuscript'.

If you would like to make changes to your financial disclosure, please include your updated statement in your cover letter. Guidelines for resubmitting your figure files are available below the reviewer comments at the end of this letter.

If applicable, we recommend that you deposit your laboratory protocols in protocols.io to enhance the reproducibility of your results. Protocols.io assigns your protocol its own identifier (DOI) so that it can be cited independently in the future. For instructions see: https://journals.plos.org/plosone/s/submission-guidelines#loc-laboratory-protocols. Additionally, PLOS ONE offers an option for publishing peer-reviewed Lab Protocol articles, which describe protocols hosted on protocols.io. Read more information on sharing protocols at https://plos.org/protocols?utm_medium=editorial-email&utm_source=authorletters&utm_campaign=protocols.

As the corresponding author, your ORCID iD is verified in the submission system and will appear in the published article. PLOS supports the use of ORCID, and we encourage all coauthors to register for an ORCID iD and use it as well. Please encourage your coauthors to verify their ORCID iD within the submission system before final acceptance, as unverified ORCID iDs will not appear in the published article. *Only*  the individual author can complete the verification step; PLOS staff *cannot*  verify ORCID iDs on behalf of authors.

We look forward to receiving your revised manuscript.

Kind regards,

Alberto Barbaresi

Academic Editor

PLOS One

Journal Requirements:

If the reviewer comments include a recommendation to cite specific previously published works, please review and evaluate these publications to determine whether they are relevant and should be cited. There is no requirement to cite these works unless the editor has indicated otherwise.

Please review your reference list to ensure that it is complete and correct. If you have cited papers that have been retracted, please include the rationale for doing so in the manuscript text, or remove these references and replace them with relevant current references. Any changes to the reference list should be mentioned in the rebuttal letter that accompanies your revised manuscript. If you need to cite a retracted article, indicate the article’s retracted status in the References list and also include a citation and full reference for the retraction notice.

[Note: HTML markup is below. Please do not edit.]

Reviewers' comments:

Reviewer's Responses to Questions

**Comments to the Author**

1. If the authors have adequately addressed your comments raised in a previous round of review and you feel that this manuscript is now acceptable for publication, you may indicate that here to bypass the “Comments to the Author” section, enter your conflict of interest statement in the “Confidential to Editor” section, and submit your "Accept" recommendation.

Reviewer #1: All comments have been addressed

Reviewer #2: All comments have been addressed

**********

2. Is the manuscript technically sound, and do the data support the conclusions?

The manuscript must describe a technically sound piece of scientific research with data that supports the conclusions. Experiments must have been conducted rigorously, with appropriate controls, replication, and sample sizes. The conclusions must be drawn appropriately based on the data presented.

Reviewer #1: Yes

Reviewer #2: Yes

**********

3. Has the statistical analysis been performed appropriately and rigorously?

Reviewer #1: Yes

Reviewer #2: N/A

**********

4. Have the authors made all data underlying the findings in their manuscript fully available?

The PLOS Data policy requires authors to make all data underlying the findings described in their manuscript fully available without restriction, with rare exception (please refer to the Data Availability Statement in the manuscript PDF file). The data should be provided as part of the manuscript or its supporting information, or deposited to a public repository. For example, in addition to summary statistics, the data points behind means, medians and variance measures should be available. If there are restrictions on publicly sharing data—e.g. participant privacy or use of data from a third party—those must be specified.

Reviewer #1: (No Response)

Reviewer #2: No

**********

5. Is the manuscript presented in an intelligible fashion and written in standard English?

PLOS ONE does not copyedit accepted manuscripts, so the language in submitted articles must be clear, correct, and unambiguous. Any typographical or grammatical errors should be corrected at revision, so please note any specific errors here.

Reviewer #1: Yes

Reviewer #2: Yes

**********

6. Review Comments to the Author

Please use the space provided to explain your answers to the questions above. You may also include additional comments for the author, including concerns about dual publication, research ethics, or publication ethics. (Please upload your review as an attachment if it exceeds 20,000 characters)

Reviewer #1: The authors addressed all the suggested revisions, and the paper has now been significantly improved. I recommend publication in its current form.

Reviewer #2: This version is substantially improved in terms of the study methodology and implications, and for that reason I accept this manuscript.

However, there are many technical improvements needed:

1) Please reduce redundancy. The paper has too many sections that are repetitive.

2) Delete sections that do not have connection with the main focus of the paper, i.e., comparison of other methods (and why they were not used).

3) Combine results with implications.

4) Eliminate results that only serve as description, and instead discuss them. The description of numbers is good for a report, but this is a journal article.

5) Ecological Business models were not explained well, and must be stated what is for.

6) Many of the explanations are abstract, only citing literature. The paper must be more practical. If mentioning an implication in eutrophication, then explain if this is valid in the literature.

7) Conclusions are too strong for qualitative analysis: It mentions cancer-related factors as causal. This must be carefully reviewed because many people from medicine can criticize it.

**********

7. PLOS authors have the option to publish the peer review history of their article (what does this mean?). If published, this will include your full peer review and any attached files.

If you choose “no”, your identity will remain anonymous but your review may still be made public.

**Do you want your identity to be public for this peer review?**  For information about this choice, including consent withdrawal, please see our Privacy Policy.

Reviewer #1: No

Reviewer #2: No

**********

[NOTE: If reviewer comments were submitted as an attachment file, they will be attached to this email and accessible via the submission site. Please log into your account, locate the manuscript record, and check for the action link "View Attachments". If this link does not appear, there are no attachment files.]

To ensure your figures meet our technical requirements, please review our figure guidelines: https://journals.plos.org/plosone/s/figures

You may also use PLOS’s free figure tool, NAAS, to help you prepare publication quality figures: https://journals.plos.org/plosone/s/figures#loc-tools-for-figure-preparation.

NAAS will assess whether your figures meet our technical requirements by comparing each figure against our figure specifications.

Attachment Submitted filename: Plus ONE - Business model of Biostimulants R1.pdf

# Author comment: Author response to Decision Letter 2

**DOI:** 10.1371/journal.pone.0343143.r004

10 Jun 2026

Dear Editor and Reviewers,

We thank the Academic Editor and the Reviewers for the constructive evaluation of our revised manuscript. We are grateful that both reviewers considered the previous comments addressed and that Reviewer #2 found the manuscript substantially improved and acceptable. In response to the remaining technical suggestions, we have further revised the manuscript to improve concision, practical clarity and alignment between results and implications. We also simplified the integrated LCA-SBM framework figure to make the modelling logic clearer.

Response to Reviewer #2

• Please reduce redundancy. The paper has too many sections that are repetitive.

We agree and have substantially streamlined the manuscript. Repetitive passages in the Introduction, LCA results, sustainable business model results, Discussion and Conclusions were shortened or removed. In particular, Sections 4.1.1, 4.1.2 and 4.1.3 were rewritten to avoid repeated category-by-category reporting, while Section 4.2 and the Conclusions were condensed to focus on the central LCA-to-business-model contribution.

• Delete sections that do not have connection with the main focus of the paper, i.e., comparison of other methods and why they were not used.

We removed or substantially reduced text that compared alternative business model tools where this was not necessary for the main argument. The revised version keeps only the minimum theoretical justification needed to explain why the SBMC and EBME are appropriate for an ecosystem-oriented, LCA-informed business model design approach.

• Combine results with implications.

We revised the Results and Discussion so that the empirical findings are immediately linked to their implications. The relative contribution analysis now explains how Fig. 2 and Fig. 3 identify the actors and activities responsible for mitigation. Section 4.2 then uses these hotspots to specify operational levers, monitoring needs, value capture mechanisms and scaling choices.

• Eliminate results that only serve as description and instead discuss them. The description of numbers is good for a report, but this is a journal article.

We rewrote the LCA result sections to shift from numerical reporting to interpretation. Values are now reported only when they support a key comparison. The revised text emphasises patterns, trade-offs and hotspot logic, such as the dominance of nutrient-related field emissions in the baseline and the emergence of upstream extract-related burdens in selected categories in the alternative scenario.

• Ecological Business models were not explained well and must be stated what is for.

We clarified the role of EBME in both the Methods and the Discussion. The manuscript now explains that EBME is used to interpret SOMMELIER as a system of interdependent business model roles rather than as a single firm-level model. In practical terms, EBME helps identify how residue segregation by the processor, extract production, laboratory verification, agronomic advisory services and farmer adoption must be aligned for the pathway to function.

• Many of the explanations are abstract, only citing literature. The paper must be more practical. If mentioning an implication in eutrophication, then explain if this is valid in the literature.

We made the discussion more concrete by linking the LCA patterns to practical agricultural and managerial decisions. The text now explains that reductions in acidification and eutrophication are relevant because these categories are closely associated with nutrient-related field emissions in agricultural systems. We also added operational variables that managers can monitor during scaling, including energy use per litre of extract, minimum effective dose, batch-level testing, traceability and verification costs.

• Conclusions are too strong for qualitative analysis: It mentions cancer-related factors as causal. This must be carefully reviewed because many people from medicine can criticize it.

We carefully revised the Abstract, Results, Discussion and Conclusions to avoid any wording that could be interpreted as a medical or causal claim. The manuscript now refers to EF toxicity-related indicators and selected trade-offs rather than cancer-related causal effects. The Conclusions were also softened and shortened and now emphasise the pilot-scale nature of the evidence, methodological limitations and the need for further agronomic and techno-economic validation.

Thank you!

Attachment Submitted filename: Rebuttal_letter_r2.docx

# Editor report: Decision Letter 2

**DOI:** 10.1371/journal.pone.0343143.r005

**Authors:** Alberto Barbaresi, Alberto Barbaresi, Alberto Barbaresi

21 Jun 2026

Evidence-based sustainable business model design for agrifood side-stream biostimulants

PONE-D-26-03978R2

Dear Dr. Zilia,

We’re pleased to inform you that your manuscript has been judged scientifically suitable for publication and will be formally accepted for publication once it meets all outstanding technical requirements.

Within one week, you’ll receive an e-mail detailing the required amendments. When these have been addressed, you’ll receive a formal acceptance letter and your manuscript will be scheduled for publication.

An invoice will be generated when your article is formally accepted. Please note, if your institution has a publishing partnership with PLOS and your article meets the relevant criteria, all or part of your publication costs will be covered. Please make sure your user information is up-to-date by logging into Editorial Manager at Editorial Manager® and clicking the ‘Update My Information' link at the top of the page. For questions related to billing, please contact billing support.

If your institution or institutions have a press office, please notify them about your upcoming paper to help maximize its impact. If they’ll be preparing press materials, please inform our press team as soon as possible -- no later than 48 hours after receiving the formal acceptance. Your manuscript will remain under strict press embargo until 2 pm Eastern Time on the date of publication. For more information, please contact onepress@plos.org.

Kind regards,

Alberto Barbaresi

Academic Editor

PLOS One

Additional Editor Comments (optional):

Reviewers' comments:

# Editor report: Acceptance letter

**DOI:** 10.1371/journal.pone.0343143.r006

**Authors:** Alberto Barbaresi, Alberto Barbaresi, Alberto Barbaresi

PONE-D-26-03978R2

PLOS One

Dear Dr. Zilia,

I'm pleased to inform you that your manuscript has been deemed suitable for publication in PLOS One. Congratulations! Your manuscript is now being handed over to our production team.

At this stage, our production department will prepare your paper for publication. This includes ensuring the following:

* All references, tables, and figures are properly cited

* All relevant supporting information is included in the manuscript submission,

* There are no issues that prevent the paper from being properly typeset

You will receive further instructions from the production team, including instructions on how to review your proof when it is ready. Please keep in mind that we are working through a large volume of accepted articles, so please give us a few days to review your paper and let you know the next and final steps.

Lastly, if your institution or institutions have a press office, please let them know about your upcoming paper now to help maximize its impact. If they'll be preparing press materials, please inform our press team within the next 48 hours. Your manuscript will remain under strict press embargo until 2 pm Eastern Time on the date of publication. For more information, please contact onepress@plos.org.

You will receive an invoice from PLOS for your publication fee after your manuscript has reached the completed accept phase. If you receive an email requesting payment before acceptance or for any other service, this may be a phishing scheme. Learn how to identify phishing emails and protect your accounts at https://explore.plos.org/phishing.

If we can help with anything else, please email us at customercare@plos.org.

Thank you for submitting your work to PLOS ONE and supporting open access.

Kind regards,

PLOS ONE Editorial Office Staff

on behalf of

Dr. Alberto Barbaresi

Academic Editor

PLOS One
